# Interactions Between *Bacillus atrophaeus* 100 MTN1 and *Fusarium oxysporum* f. sp. *lycopersici* Reprogram the Transcriptomic and Metabolomic Profile to Combat Tomato (cv. Kalyan) Wilt

**DOI:** 10.3390/microorganisms14071488

**Published:** 2026-07-07

**Authors:** Ramachandran Muthulakshmi Vijaya Ramakrishnan, Perumal Renukadevi, Rangasamy Anandham, Mathiyazhagan Kavino, Anbu Kokila, Shafat Ahmad Ahanger, Mareyam Mukhtar, Khalid E. Hamed, Mohammad Mahamood, Suhail Ashraf, Mona Saleh Al Tami, Sevugapperumal Nakkeeran

**Affiliations:** 1 Department of Plant Pathology, Tamil Nadu Agricultural University, Coimbatore 641003, India; vijayaramakrishnanrm@gmail.com (R.M.V.R.); renukadevi.p@tnau.ac.in (P.R.); 2 Department of Microbiology, Tamil Nadu Agricultural University, Coimbatore 641003, India; anandhamranga@gmail.com; 3 Department of Fruit Science, Horticultural College and Research Institute, Tamil Nadu Agricultural University, Coimbatore 641003, India; kavino.m@tnau.ac.in; 4 Department of Plant Biotechnology, Centre for Plant Molecular Biology & Biotechnology, Tamil Nadu Agricultural University, Coimbatore 641003, India; kokila95.anbalagan@gmail.com (A.K.); mareyam.mukhtar@niab.com (M.M.); suhailashraf9906@gmail.com (S.A.); 5 College of Plant Protection, Northwest A&F University, Yangling 712100, China; shafatahanger99@nwafu.edu.cn; 6 Division of Plant Pathology, Sher-e-Kashmir University of Agricultural Sciences and Technology of Kashmir, Jammu & Kashmir, Srinagar 190025, India; 7 National Institute of Agricultural Botany (NIAB), Park Farm Campus, Villa Road, Histon, Cambridge CB24 9AT, UK; 8 Department of Plant Protection, College of Agriculture and Food, Qassim University, P.O. Box 6622, Buraidah 51452, Qassim, Saudi Arabia; kh.mohammed@qu.edu.sa (K.E.H.); m.mahamood@qu.edu.sa (M.M.); 9 Department of Biology, College of Science, Qassim University, P.O. Box 6622, Buraidah 51452, Qassim, Saudi Arabia

**Keywords:** sugarcane bagasse, biocontrol, antifungal biomolecules, transcriptome analysis, fusarium wilt, tomato, molecular docking

## Abstract

This research evaluated the biocontrol potential of the bacterial flora from cured sugarcane bagasse (SCB) against *Fusarium oxysporum* f. sp. *lycopersici* (*Fol*), the causal agent of tomato Fusarium wilt. Screenings of twenty SCB-derived isolates revealed consistent antagonistic activity, inhibiting mycelial growth from 32.08% to 55.00%. The most effective isolate, 100MTN1, was identified via 16S rRNA sequencing (GenBank: PX506225) as *Bacillus atrophaeus*. Interaction between *B. atrophaeus* 100MTN1 and *Fol* FOLViF has revealed a distinct profile of bioactive metabolites produced specifically during co-cultivation. Transcriptomic profiling of *Fol* FOLViF exposure to 100MTN1 identified 189 differentially expressed genes, with downregulation of genes involved in DNA replication, translation, and membrane transport, and upregulation of those linked to secondary metabolism and oxidative stress. KEGG pathway mapping further supported the possible causes of disruptions within the pathogen. Molecular docking suggested that the *B. atrophaeus* 100MTN1 derived metabolite, 6-Hydroxy-3′-methoxyflavone and exhibits binding affinity for key *Fol* proteins that compares favorably with the commercial fungicides. Greenhouse trials using tomato cv. Kalyan confirmed that treatment with strain 100MTN1 was associated with reduced disease severity and enhanced plant growth. These findings suggest that *B. atrophaeus* 100MTN1 suppresses *Fol* FOLViF through a combination of metabolite-driven inhibition and transcriptional interference, signifying its potential as a biological control agent for managing Fusarium wilt.

## 1. Introduction

The cultivation of tomato (*Solanum lycopersicum* L.) stands as a cornerstone of global horticulture, providing significant economic value and essential dietary nutrients. However, sustainable production is severely constrained by soil-borne fungal pathogens, most notably *Fusarium oxysporum* f. sp. *lycopersici* (*Fol*), the inciting agent of Fusarium wilt [1]. The vascular pathogen invades the root system and colonizes xylem vessels, leading to characteristic symptoms such as leaf chlorosis, progressive wilting, and systemic plant collapse, which culminate in substantial yield losses. The management of *Fol* is difficult due to its long-term persistence in the soil as dormant chlamydospores and due to its high level of physiological variability, which enables the pathogen to overcome resistant host plants. Traditional chemical interventions are scrutinized intensively due to the inconsistent field efficacy and their detrimental impacts on human health and environmental hazards, including the emergence of resistant pathogen populations [2]. Consequently, there is an urgent global shift towards identifying sustainable, bio-based alternatives for disease suppression and management.

At this juncture, the exploitation of microbial communities represents a resource for the discovery of novel biocontrol agents. Bacterial genera including *Burkholderia*, *Bacillus*, *Pseudomonas*, *Brachybacterium*, and *Acinetobacter* have been identified as key players in the production of secondary metabolites, cell wall-degrading enzymes, and volatile organic compounds to survive and compete with other microbial communities [3,4]. Hence, mining the potential of bacterial microbiome serves as a repository for discovering novel biocontrol agents capable of rapid rhizosphere colonization and plant growth promotion (PGP) [5,6].

The disease suppression mediated by the potential antagonistic bacteria associated with rhizosphere and organic soilless media like coco-peat (CP) and sugarcane bagasse (SCB) involves complex interactions with the rhizosphere microbiome. Decades of research with respect to fluorescent pseudomonads and *Bacillus* spp. directly suppress the growth of plant pathogens through the production of 2,4-diacetylphloroglucinol, iturin, surfactin and fengycin [7,8,9,10,11]. A variety of beneficial rhizobacterial genera, including *Bacillus* [12,13], *Pseudomonas* [8,9,10], *Paenibacillus* [14], and *Streptomyces* [11,15], have been recognized for their ability to suppress diseases in soil and soilless cultivation. In the recent past, cultivation of vegetables and ornamentals using soilless media under protected cultivation has gained momentum. In general, as the C:N ratio of SCB is very high (120:1), attempts were made to cure the SCB so as to develop an alternate soilless media to coco-peat. During the process of curing, several bacterial genera were isolated. Among them, the dominance of Firmicutes, especially *Bacillus* spp., was frequently noticed. It was subjected to antifungal activity against *Fol*. Among the different *Bacillus* spp., *B. atrophaeus* inhibited the mycelial growth of *Fol*. A further literature survey also clearly indicated that *B. atrophaeus* has not been explored for the management of soil-borne pathogens of major vegetable crops. Hitherto, the genomic configuration of *B. atrophaeus* from the literature clearly indicated the presence of antimicrobial peptides, lytic enzymes, defense genes and genes for plant growth promotion. Considering the potential of *B. atrophaeus*, the present investigation was made to mine the antifungal efficacy of *B. atrophaeus* 100MTN1.

Additionally, they are also known for the induction of innate immunity against plant pathogens. However, the antifungal potential of *B*. *atrophaeus* has not been explored to combat plant pathogens. Considering this research gap, we investigated the effect of *B. atrophaeus* 100MTN1 in tomato to suppress the activity of *Fol* FOLViF and to quench Fusarium wilt in tomato, employing both transcriptomic and metabolomic approaches.

## 2. Materials and Methods

### 2.1. Isolation of Bacterial Antagonist and Assessment of Antifungal Activity

Bacterial antagonists were isolated from cured sugarcane bagasse (SCB) during the curing process. For the isolation of bacteria, representative samples were collected from a 60-day-old cured SCB. Ten samples comprising 500 g each were collected from 10 different locations of the SCB bay of 5 tons capacity, maintained at 70% moisture and 60 °C. Care was taken to exclude SCB from the top surface, and the samples were collected at 1 feet depth from the surface of the bay. One hundred grams of this mixture was placed in sterile polybags and brought to the laboratory for further analysis. The collected representative samples were homogenized, and from the homogenized sample, 1 g was collected and serially diluted in sterile water till 10^6^ dilution, plated in sterile LB agar medium (10 g trypton (HiMedia, Mumbai, India), 5 g yeast extract (HiMedia), 5 g sodium chloride (HiMedia) and 20 g Agar Agar (HiMedia)) and incubated at room temperature, 28 ± 2 °C [16]. The individual colonies that appeared on the surface of the medium were purified, characterized and maintained in glycerol stock at −40 °C. For further studies, the cultures were retrieved from the glycerol stock and used for assessing the antifungal efficacy against *Fol* FOLViF (Accn. No: PX671077) using a dual confrontation assay [17]. In this assay, a 5 mm mycelial plug was excised from a 7-day-old culture of *Fol* FOLViF and was placed on one side of a sterile Petri plate containing sterile PDA medium (250 g potato, 20 g dextrose (HiMedia) and 20 g Agar Agar (HiMedia)). The bacterial cultures were adjusted to an optical density (OD600) of 0.5 before assessing for the antifungal activity, and then a loop full of each isolate was streaked on the opposite side, about 1 cm from the plate edge, directly facing the fungal plug. Control plates had only the fungal plug with no bacteria. All the plates were incubated at 28 ± 2 °C for 5 days, and fungal growth inhibition was assessed. To measure fungal growth, the radial growth was recorded from the colony center to the margin using a ruler. Percent inhibition was calculated as (C − T)/C × 100, where C is the radial growth of *Fol* FOLViF on control plates, and T is the radial growth of *Fol* FOLViF in the antagonized plates with bacterial antagonists. The isolate expressing the highest mycelial inhibition was selected for further studies.

### 2.2. Identification and Characterization of the Most Potent Bacterial Antagonist

Genomic DNA (gDNA) was extracted from the effective bacterial antagonist (30 mL) grown in nutrient broth, centrifuged at 12,000 rpm for 5 min at 5 °C, and the supernatant was discarded. The bacterial pellet was treated with phenol–chloroform to extract gDNA. The extracted gDNA was amplified by PCR using a universal 16S primer pair consisting of 27F (5′-AGA GTT TGA TCM TGG CTC AG-3′) and 1492R (5′-TAC GGY TAC CTT GTT ACG ACT-3′), targeting the nearly full-length 16S rRNA gene. A 10 µL reaction mixture including 5 µL of PCR master mix (AMPLIQON, DK-5230 Odense M, Denmark), 1 µL of forward primer, 1 µL of reverse primer, 1 µL of sterile distilled water and 2 µL of gDNA was used for molecular confirmation through PCR. The PCR reaction mixture adopted for amplification was initial denaturation for 5 min at 95 °C, 40 cycles of denaturation at 95 °C for 1 min, 30 s of annealing at 56 °C, 1 min of extension at 72 °C, and a final extension step at 72 °C for 10 min. Amplifications were carried out in duplicate for each sample, and the number of expected PCR amplicons was confirmed using agarose gel electrophoresis. The PCR products (35 µL) and forward and reverse primers (20 µL each) were sent for Sanger sequencing. The sequences were then identified using NCBI-BLASTn databases and submitted to NCBI Genbank. CLUSTALW was used for multiple sequence alignment [18], and MEGA 11 software (version 11.0.13) was used for the construction of a phylogram with a bootstrap consensus generated with 1000 replications [19]. The most potent bacteria were double-confirmed with 16S rRNA identification in the EzBioCloud (https://www.ezbiocloud.net) database.

### 2.3. Metabolite Extraction and Identification of Biomolecules from the Zone of Inhibition Produced by B. atrophaeus 100MTN1 (PX506225) Against Fol FOLViF

During this study, we were interested in examining the variably expressed volatile (VOCs) and non-volatile (NVOCs) organic compounds during dual assay between the *B. atrophaeus* 100MTN1 and pathogenic *Fol* FOLViF. Bioactive VOCs/NVOCs diffused into the agar at the zone of inhibition between *Fol* FOLViF and *B. atrophaeus* 100MTN1 were extracted [20]. The extracted biomolecules from the diffused agar were then subjected to metabolic profiling using Gas Chromatography–Mass Spectrometry (GC-MS) to identify the specific compounds present in the zone of inhibition [16]. VOCs/NVOCs were analyzed using the Clarus SQ 8C Gas Chromatography–Mass Spectrometer from Perkin Elmer, Waltham, MA 02451, USA. The instrument was set as follows: injector port temperature set to 220 °C, interface temperature set to 250 °C, source kept at 220 °C. The oven temperature was programmed as available: 75 °C for 2 min, 150 °C @ 10 °C /min, up to 250 °C @ 10 °C/min. Split ratio was set as 1:12, and the injector used was in split-less mode. The DB-5 MS capillary standard non-polar column was used. Its dimension was 0.25 mm OD × 0.25 μm ID × 30 m length (Agilent Co., Santa Clara, CA 95054, USA make). Helium was used as the carrier gas at 1 mL/min. The MS was set to scan from 50 to 550 Da. The source was maintained at 220 °C and 4.5 × 10^−6^ mtorr vacuum pressure. The ionization energy was −70 eV. The MS also had an inbuilt pre-filter, which reduced the neutral particles. The data system has inbuilt NIST libraries for searching and matching the spectrum. NIST MS Search 2.2v contains more than five lakh references. Interpretation of the mass spectrum of GC–MS was done using the database of the National Institute of Standards and Technology (NIST14). The spectrum of the known component was compared with the spectrum of the known stored components in the inbuilt library. A mass spectral match score threshold of >800 (out of 999) or 80% was applied as the cutoff for tentative compound identification; hits with scores below this threshold were excluded from the analysis. Since HPLC-grade methanol was used for the extraction of the metabolites, it was used as a blank control to eliminate any background noise.

### 2.4. RNA Extraction and Quality Assessment

Total RNA was extracted from 100 mg of the mycelium of *Fol* FOLViF away from the zone of inhibition in the Petri plates antagonized by *B. atrophaeus* 100MTN1 after five days of incubation at 28 ± 2 °C, using the total RNA isolation kit (Qiagen, Colonia Loreto C.P. 01090, México) as per the manufacturer’s instructions. Quantitative and qualitative analyses were performed using the Nanodrop method and agarose gel electrophoresis, respectively.

### 2.5. Library Preparation and Sequencing

Three replications for each condition (*Fol* FOLViF control and *Fol* FOLViF antagonized by *B. atrophaeus* 100MTN1) were maintained. Fifty nanograms of total RNA was reverse transcribed, and samples were barcoded with the PCR cDNA Barcoding Kit (SQK-PCB109, Oxford Nanopore Technologies, Oxford, UK). A total of ~50 ng of amplified cDNA was cleaned up with 1× AmPure beads (Beckmann Coulter, Brea, CA 92821, USA). The quantity of the purified cDNA was then determined with the Nanodrop method. The Flowcell priming kit (EXP FLP002, Oxford Nanopore Technologies, Cambridge, MA 02139, USA) was used to prime the Flowcell (FLO-MIN106), and an equal amount of barcoded cDNA was loaded. Sequencing was carried out with a MinION (MN33710, Oxford Nanopore Technologies plc) using the MinKNOW software (v.21.02.1) over a period of 72 h.

### 2.6. Raw Read Processing

Nanopore raw reads (‘fast5’ format) were base-called (‘fastq5’ format) and demultiplexed using Guppy1 v2.3.4. By this, reads with a quality score below 7 were excluded. The adapter and barcode sequences were trimmed using Porechop (Oxford Nanopore Technologies). The average Phred quality score was assessed using Nano-Plot ver. 1.27.0.

### 2.7. Quality Control of Raw Reads

Raw reads were cleaned by adopting the core algorithm from the fastp v0.20.0 software [21], trimmed at both the 5′ and 3′ ends. The poly (G) tail was removed. Low complexity sequences were removed as they can cause artificially high protein hit scores during protein alignment. Sequence adaptors and poly (A) tails were identified and removed. Overlapping read ends were merged into a longer single-end (SE) read. Finally, all reads were converted to FASTA format.

### 2.8. Differentially Expressed Genes (DEGs) and Gene Ontology (GO) Analysis

First, each clean read was translated into amino acid sequences using six reading frames from both directions, which typically resulted in dozens of peptide fragments. At most, the top six longest fragments were used for the translated search. Next, the peptide fragments were aligned to the Seq2Fun database, which consists of protein sequences from KEGG pathway genes that were retrieved using the KEGGREST R package v1.12.2 (https://bioconductor.org/packages/KEGGREST/ accessed on 28 March 2026). The size of the protein database was reduced by removing redundant protein sequences that have >99% similarity across species using CD-HIT v4.8.1. Seq2Fun employs the same core reads alignment algorithm as Kaiju v1.7, without (Greedy mode) a reference genome. The peptide fragments were aligned to a database that contains sequences from the fungal database, and the fragments with the highest BLOSUM62 scores were retained.

### 2.9. Expression Quantification

First, the reads were mapped to multiple protein IDs with the fungal database. The protein homologies from the same or different organisms that share the same KEGG ortholog ID were used. If this is not the case, the KEGG ortholog with the highest frequency was used. After ensuring that each read was matched to a single KEGG ortholog, the final quantification was a summation of all read KEGG ortholog matches.

### 2.10. Protein Model Validation and Molecular Docking

In the exploration of biomolecules produced by *B. atrophaeus* 100MTN1 during its interaction with *Fol* FOLViF, an in silico docking analysis was employed. The biomolecules produced in the inhibition zone during the antagonism of *B. atrophaeus* 100MTN1 with *Fol* FOLViF were used as the ligand to dock with the proteins of downregulated genes from transcriptome results. Trifloxystrobin, tebuconazole and carbendazim were used as a positive control to compare the efficiency of the biomolecules characterized from the zone of interaction. The protein structures intended for docking were obtained from UniProt. The target proteins include *ACL1* (UniProt Id: A0A0J9V2C1), *EIF3S9* (UniProt Id: A0A8H5AJ81), *PLC1* (UniProt Id: A0A0J9UKX7), *PTR2* (UniProt Id: A0A2H3T578), *RAM1* (UniProt Id: W9K7Q2), *RBK1* (UniProt Id: A0A0J9VVJ7), *RPL3* (UniProt Id: A0A2H3T300), *RPL11* (UniProt Id: A0A2H3TJE6), *RRP5* (UniProt Id: W9KU20), *SRD5A3* (UniProt Id: W9KQ10), *TFP1* (UniProt Id: A0A420QGF7), *UBA52* (UniProt Id: W9HZ76), Histone deacetylase (*HDA*) (UniProt Id: A0A0J9U343), *RBM27* (UniProt Id: A0A8M5AD19), *ARF1* (UniProt Id: A0A0J9UC74), *CDC34* (UniProt Id: A0A0J9WNK3), *SI:DKEY-3708.1* (UniProt Id: A0A0J9UJ79), *AN11G11290* (UniProt Id: A0A9P9L8S8), *RFC3* (UniProt Id: A0A0D2XUS8), *DLD1* (UniProt Id: A0A0J9WGR3), *GLN1* (UniProt Id: A0A0D2XMK5), *DDX5* (UniProt Id: F9FE48) and *HTA1* (UniProt Id: A0A0J9VID0). The protein biomolecule binding affinities were analyzed using molecular docking. These predicted proteins were analyzed for residues in permitted and preferred areas. The Ramachandran plot was employed to verify the geometric correctness of the protein models developed using the RamPlot server (https://www.ramplot.in/ accessed on 14 June 2026). The Computed Atlas of Surface Topography of Proteins (CASTp) 3.0 sever was used to identify binding site pockets for the targets. The biomolecules identified through GC-MS data were selected for molecular docking to prepare ligands. Their structure was obtained from PubChem (https://www.ncbi.nlm.nih.gov/pccompound accessed on 23 March 2026) in a Structured Data File (SDF). The structures were subsequently processed and stored in a ligand library. This library serves as a significant resource for further computational and experimental analyses. For this purpose, the AutoDock Vina module within PyRx 0.8 software was employed to assess the ligand-receptor interactions based on binding affinities. After minimizing the ligands, protein and ligand molecules were converted into AutoDock PDBQT format [Protein Data Bank, Partial Charge (Q), and Atom Type (T)]. The Grid size varied with the different permissible binding residues of each protein. With a rigid receptor, ligands with flexible conformations and orientations, and with an exhaustiveness of 8, were considered for further analysis. Biomolecules with a binding affinity of less than −5 kcal/mol and that interacted with a significant number of targets were selected. The docked complex files were visualized using the BIOVIA Discovery Studio Client 2021. Both binding affinity (specifically, complexes with values less than −5 kcal/mol) and hydrogen bonding were considered for identifying effective antifungal compounds.

### 2.11. In Planta Efficacy of B. atrophaeus on Fusarium Wilt, Seed Germination, and Plant Growth Promotion in Pot Culture

To validate the antifungal efficacy of *B. atrophaeus* 100MTN1 under glasshouse conditions, 21-day-old tomato seedlings (cv. Kalyan) were transplanted into 9-inch-diameter and 8-inch-high pots filled with 1250 g of a 1:1 ratio (weight/weight) of SCB:coco-peat mixture. The artificial media with tomato plants in the pots were watered until saturation and were maintained at 25–30 °C, 70–80% RH with a 12 h photoperiod. Conidial suspension of *Fol* FOLViF isolate was drenched into the artificial media mix with tomato plants by drenching the root zone with 50 mL of conidial suspension at a concentration of 1 × 106 conidia mL^−1^ after 15 days of post transplantation. Thirty milliliters (of a 48 h old culture of *B. atrophaeus* 100MTN1 multiplied in Luria-Bertani broth was mixed in 1 L of distilled water. These solutions were applied as root drenches 24 h post pathogen inoculation and repeated twice on 30 (Vegetative phase) and 60 days (Flowering Phase) after transplanting. The treatments included T1—*Fol* FOLViF + *Bacillus velezensis* VB7 (CP047587), T2—*Fol* FOLViF + *Bacillus tequilensis* NB LEAF 6 (MW301641), T3—*Fol* FOLViF + *Bacillus atrophaeus* 100MTN1 (PX506225), T4—*Fol* FOLViF + *Bacillus glycinifermentans* CNEB17 (MZ485782), T5—*Fol* FOLViF + *Bacillus subtilis* IBHB4 (PP805943), T6—*Fol* FOLViF only and T7—Healthy control (un-inoculated control), each with three replicates arranged in a completely randomized design (three pots per replication with two plants per pot). Hence, a total of 18 plants were transplanted per treatment. Disease severity was assessed after ninety days post-inoculation by examining yellowing of leaves, epinasty and vascular discoloration using a longitudinal section of the stem to root. The wilt incidence was determined as percent disease incidence (PDI) using the scale described by Michel and his Co-workers [22].

The PDI was calculated using the following formula:

Disease incidence (%) = (n)/N × 100

where n = number of plants showing wilt symptoms, and N = total number of plants sampled.

### 2.12. Statistical Analysis

The resulting abundance table pertaining to transcriptome analysis was analyzed statistically using the Express Analyst tool (https://www.expressanalyst.ca/ExpressAnalyst/home.xhtml, accessed on 21 February 2026). This analysis included data normalization, calculation of log fold changes, and differential expression analysis. Statistical significance was assessed using standard parameters within the Express Analyst platform to identify biologically relevant expression patterns. All greenhouse experiments were conducted with three biological replicates per treatment in a completely randomized block design. Disease severity data were arcsine transformed and tested for normality (Shapiro–Wilk test) prior to analysis. One-way ANOVA was performed using SPSS v28.0, and results were expressed as means ± standard error with error bars indicating a 95.0% confidence level.

## 3. Results

### 3.1. Isolation, Screening, and Identification of Effective Bacterial Antagonists

**Figure 1:**
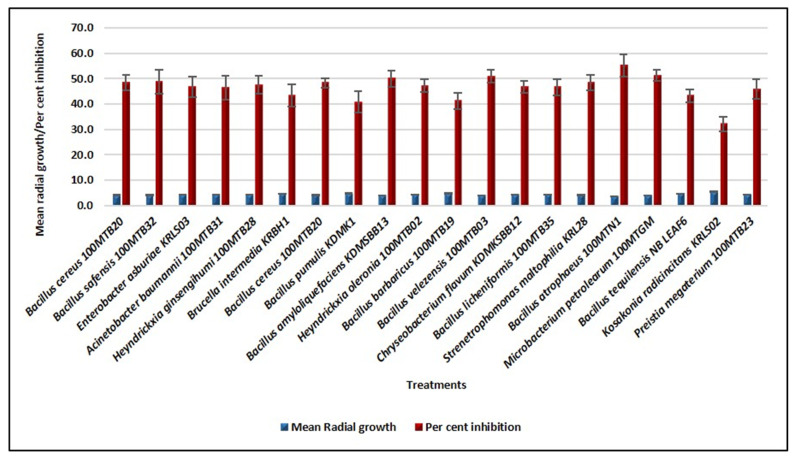
A dual-axis bar graph displaying the *in vitro* antagonistic activity of different SCB bacterial isolates against *Fol* FOLViF. The chart plots and compares the radial growth of the *Fol* FOLViF mycelium and its corresponding growth inhibition percentage.

Twenty bacterial antagonists were successfully isolated during the curing process of SCB. All 20 isolates demonstrated measurable antifungal activity, with percent inhibition ranging from 32.08% to 55.00% (Figure 1 and Figure S1). Among these, the isolate 100MTN1 displayed the highest per cent mycelial inhibition of 55.00%. Other isolates with notable antifungal efficacy included *Microbacterium petrolearum* 100MTGM (51.25%), *B. velezensis* 100MTB03 (50.83%), and *B. amyloliquefaciens* KDMSBB13 (50.00%). The *in vitro* dual culture assay was conducted in three independent trials, each with three replicates per isolate, to ensure reproducibility. *B. atrophaeus* 100MTN1 consistently demonstrated a pronounced inhibition zone, indicating its strong antagonistic effect against *Fol* FOLViF. Given its superior antifungal performance, 100MTN1 was selected for molecular identification. The 16S rRNA gene sequencing of 100MTN1 revealed a high sequence similarity of 99% and thus was identified as *B. atrophaeus* 100MTN1 (PX506225), confirming its taxonomic identity. This identification was verified against NCBI databases to ensure accuracy, leading to the designation of this isolate as *B. atrophaeus* strain 100MTN1. The 16S rRNA gene sequence of 100MTN1 was submitted to GenBank and assigned the accession number PX506225. The identification of the strain 100MTN1 was confirmed to be *B. atrophaeus* through 16S rRNA bacterial sequence identification in EzBioCloud. The sequence showed a similarity index of 98.96% and 100% query coverage with *B. atrophaeus* JCM 9070 (T) (Accession ID: AB021181) (Figure S2). Phylogenetic analysis and phylogram were constructed on the basis of variations in the 16S rRNA region using the neighbor joining method with bootstrap replication of 1000 and a cutoff value of 70% (Figure S3).

### 3.2. In Planta Efficacy of B. atrophaeus 100MTN1 on Fusarium Wilt, Seed Germination, and Plant Growth of Tomato (cv. Kalyan) in Pot Culture

The antifungal efficacy of *B. atrophaeus* 100MTN1 was evaluated under controlled glasshouse conditions using 21-day-old tomato (cv. Kalyan) seedlings challenged with *Fol* FOLViF. Significant differences in plant height and disease severity were recorded in the treatment with *B. atrophaues* 100MTN1 in comparison with the inoculated control. Plants treated with *Fol* FOLViF alone—T6 exhibited severe wilt symptoms with 86.11 ± 0.0% disease incidence (Table 1). These severely affected plants displayed characteristic Fusarium wilt symptoms, including extensive root necrosis, pronounced vascular discoloration, and complete collapse of the internal tissue, consistent with typical *Fol* FOLViF infection.

**Figure 2:**
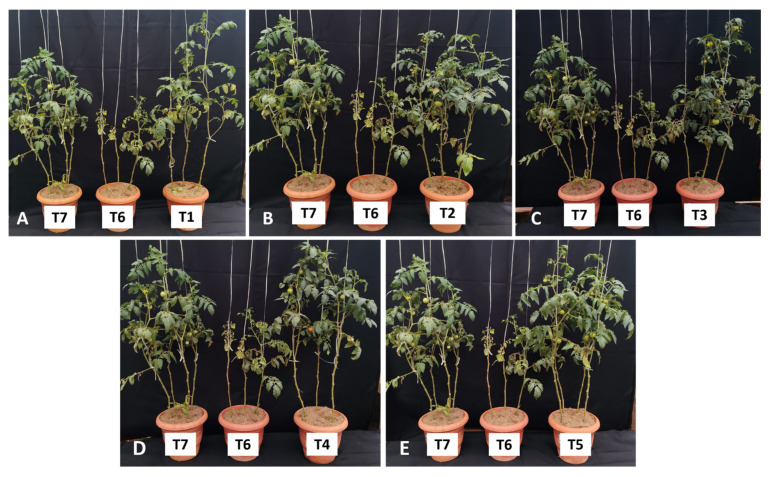
Assessment of antagonistic action by the bacterial antagonists against *Fol* FOLViF in Tomato plants cv. Kalyan. (n = 12 per treatment group) against tomato wilt. T7—un-inoculated control (left pot); T6—inoculated control—*Fol* FOLViF alone (middle pot) in comparison with (**A**) T1—*Fol* FOLViF + *B. velezensis* VB7; (**B**) T2—*Fol* FOLViF + *B. tequelensis* NB LEAF 6; (**C**) T3—*Fol* FOLViF + *B. atrophaeus* 100MTN1; (**D**) T4—*Fol* FOLViF + *B. glycinifermentans* CNEB17; (**E**) T5—*Fol* FOLViF + *B. subtilis* IBHB4.

In contrast, plants bio-augmented with *B. atrophaeus* 100MTN1 followed by the challenge inoculation with *Fol* FOLViF—T3 demonstrated a significant increase in plant height to 95.4 cm at 90 DPI and reduced the disease incidence to 18.88% (Figure 2) as against 64.1 cm of plant height and 86.11% disease incidence with inoculated control—T6 at 90 DPI. It was followed by T1—*Fol* FOLViF + *B. velezensis* VB7, which had a plant height of 93.9 cm and 24.07% of disease incidence at 90 DPI. It was succeeded with T2—*Fol* FOLViF + *B. tequilensis* NB LEAF 6 with a plant height of 91.3 cm and 28.57% disease incidence. The plants treated with *Fol* FOLViF + *B. glycinifermentans* CNEB17 were recorded with a plant height of 90.1 cm and a disease incidence of 33.13%, followed by T5—*Fol* FOLViF + *B. subtilis* IBHB4, with a plant height of 83.2 cm and a disease incidence of 36.11% at 90 DPI. The plant height of the healthy control was observed as 77.8 cm at 90 DPI (Tables S1–S4).

### 3.3. Identification of Biomolecules from the Zone of Inhibition Produced by B. atrophaeus 100MTN1 Against Fol FOLViF

To characterize the bioactive compounds responsible for the observed antagonistic activity under in vitro assays, biomolecules were extracted from the zone of inhibition formed during the dual culture interaction between *B. atrophaeus* 100MTN1 and *Fol* FOLViF and analyzed by GC-MS. The metabolic profile obtained from the dual culture was distinctly different from those of *Fol* FOLViF or *B. atrophaeus* 100MTN1 alone, indicating that the interaction induces the production of a unique set of compounds. Compounds detected from the dual culture of *Fol* FOLViF and *B. atrophaeus* 100MTN1 included 5-Hydroxymethylfurfural, scoring the highest peak area percentage of 12.127% with a retention time of 7.815, followed by dl-Glyceraldehyde dimer with a peak area of 9.537% with the retention time of 3.504. The biomolecules with peak area percentage of 5% and below were pertaining to Propanoic acid, Hexanoic acid, Ethyl 2-nitropropionate, Glycyl-dl-serine, 2-Furancarboxylic acid, Clindamycin, N-Ethylmaleimide, 4H-Pyran-4-one, 2,3-dihydro-3,5-dihydroxy-6-methyl, N-Butyryl-L-homoserine lactone, 1,2,3-Propanetriol, 2′,4′-Dihydroxychalcone, Cyclohexane acetic acid, N-Formyl-l-methionine, N-Nitroso-2,4,4-trimethyloxazolidine, 2-Cyclohexylpiperidine, 3-Heptanol, Pentanoic acid, 2-Decenoic acid, 1,2-Heptanediol, 1,3-Dioxepane, 3,4-O-Isopropylidene-d-galactose, 2-t-Butyl-5-propyl-[1,3]dioxolan-4-one, 6-Tridecanol, d-Glycero-d-ido-heptose, 6-Hydroxy-3′-methoxyflavone, Ethylene glycol diallyl ether, Tetradecanoic acid, Ethylene glycol diallyl ether and n-Hexadecanoic acid. Finally, the compounds produced by *Fol* FOLViF alone are Cycloheptasiloxane, Cefotaxime, Glafenin, Octasiloxane, Hexadecanoic acid, Oxiranedodecanoic acid, Tetradecanamide, Glycidyl palmitate, Octadecenamide, Octadecadienoic acid and Glycidyl oleate (Figure S4). These differences in metabolic profiles suggest that the plausible antagonistic activity of *B. atrophaeus* 100MTN1 is associated with the production of presumptively identified bioactive compounds induced during direct interaction with the pathogen.

### 3.4. Transcriptome Sequencing and Quality Assessment

RNA sequencing of *Fol* FOLViF (PX671077) during the interaction with *B. atrophaeus* 100MTN1 generated high-quality transcriptome data suitable for comprehensive analysis. The sequencing depth ranged approximately from 1,454,733 to 1,674,027 reads per sample across all replicates. This depth of sequencing provided a solid foundation for differential expression analysis. Principal Component Analysis (PCA) of the normalized expression data resulted in a distinct clustering between the *B. atrophaeus* 100MTN1-treated and control groups, indicating a transcriptional shift by the interaction of *B. atrophaeus* 100MTN1 (Figure S5) with *Fol* FOLViF. The raw reads are deposited in NCBI under the bioproject ID: PRJNA1476957. The accession number for the raw reads for each replication of the two conditions was *Fol* FOLViF control R1: SAMN60749114; *Fol* FOLViF control R2: SAMN60749115; *Fol* FOLViF control R3: SAMN60749116; *Fol* FOLViF antagonized by *B. atrophaeus* 100MTN1 R1: SAMN60749117; *Fol* FOLViF antagonized by *B. atrophaeus* 100MTN1 R2: SAMN60749118; *Fol* FOLViF antagonized by *B. atrophaeus* 100MTN1 R3: SAMN60749119.

### 3.5. Analysis of Differentially Expressed Genes (DEGs) During the Interaction of B. atrophaeus 100MTN1 with Fol FOLViF

Transcriptome analysis of *Fol* FOLViF mycelium harvested from the interaction zone between *B. atrophaeus* 100MTN1 and *Fol* FOLViF revealed the transcriptional changes in genes associated with various metabolic processes. A total of 189 differentially expressed genes (DEGs) were identified. Among these, 83 genes were downregulated, 87 genes were upregulated, and 19 genes remained nonsignificant (|log2FC| > 1, FDR < 0.05). To provide a high-resolution view of the most impactful transcriptomic changes, a selected subset of 189 DEGs has been highlighted and labeled in the volcano plot (Figure S6). This subset prioritizing [adj. P. Val 2] served to illustrate the most robust and biologically relevant components of the broader 189 DEG pool alone. The genes altered the most were predominantly associated with cellular and metabolic functions, as well as membrane transport. Genes involved in translation, central carbon metabolism and membrane transportation were downregulated, indicating impaired protein turnover and efflux mechanism. In contrast, genes related to secondary metabolism and oxidative stress response were elevated, indicating a defense response mediated by *B. atrophaeus* 100MTN1.

### 3.6. Functional Annotation and Gene Ontology (GO) Analysis

Gene Ontology (GO) analysis classified the differentially expressed genes into three major categories, viz., biological processes, molecular functions, and cellular components.

#### 3.6.1. GO Analysis of Biological Process

**Figure 3:**
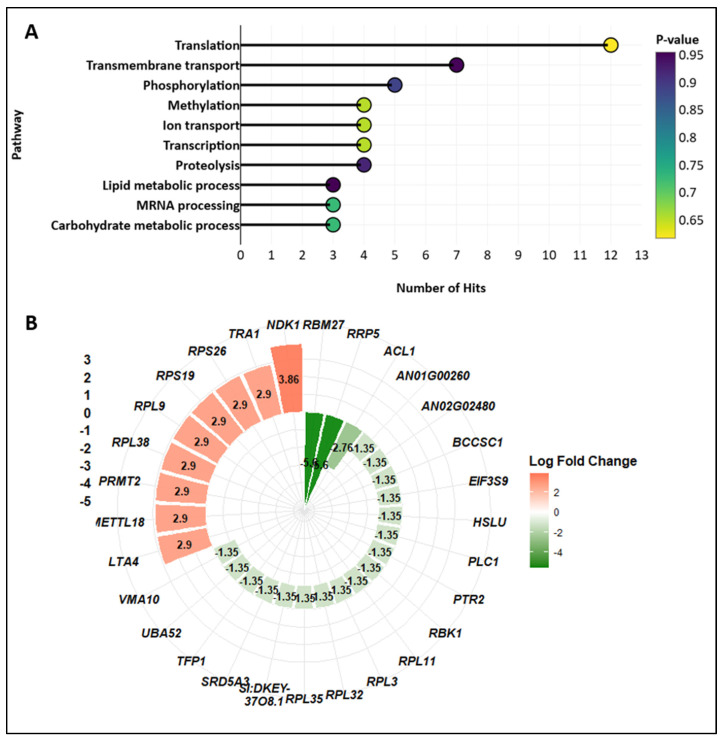
(**A**) Gene Ontology (GO) enrichment analysis of biological processes in the mycelium of *Fol* FOLViF antagonized by *B. atrophaeus* 100MTN1. The bar chart displays the number of genes associated with enriched biological processes following *B. atrophaeus* 100MTN1 treatment compared to untreated controls. The x-axis shows the number of genes involved in each pathway, with the translation process showing the highest enrichment (12 genes), followed by transmembrane transport (7 genes). (**B**) Fold changes of transcripts associated with biological processes in *Fol* FOLViF antagonized by *B. atrophaeus* 100MTN1.

According to the Gene Ontology (GO) analysis, several changes were observed in important biological processes of *Fol* FOLViF upon interaction with *B. atrophaeus* 100MTN1, offering insights into its possible antifungal activity (Table 2, Figure 3). The analysis revealed substantial reorganization of the protein synthesis machinery, with a notable impact on translation-related processes involving several differentially expressed genes. The expression profile of these translation-associated genes suggested a targeted downscaling (*p*-value: 0.616) of specific protein synthesis components, such as *RPL32*, *RPL3*, *RPL35*, *RPL11*, and *UBA52*. Conversely, genes such as *RPS26*, *RPL9*, *RPS19*, and *RPL38* were upregulated. It suggested a selective remodeling of the ribosome during the stress response. Crucial genes involved in mRNA processing, including *RRP5* (ribosomal RNA processing), *RBM27* (mRNA quality control), and *AN02G01220* (mRNA splicing factor), were downregulated (*p*-value: 0.726), consistent with coordinated suppression of transcript maturation. Phosphorylation-related processes revealed a dynamic response (*p*-value: 0.887) with the downregulation of *RBK1* (ribokinase 1), while the genes involved in the energy generation pathway, such as *NDK1* (nucleoside diphosphate kinase 1) and *TRA1* (SAGA: Spt-Ada-Gcn5-acetyltransferase and NuA4 histone acetyltransferase complexes), were upregulated. In the lipid metabolic process, uniform downregulation was observed for *SRD5A3* (steroid 5-alpha reductase 3), *PLC1* (phospholipase C 1), and *ACL1* (ATP citrate lyase), with a *p*-value of 0.726, indicating a potential compromise in membrane lipid biosynthesis. Genes *PTR2* (Proton-dependent oligopeptide transporter) and *SNF3* involved in the transmembrane transport process, and the genes *TFP1* (V-type proton ATPase catalytic subunit A) and *VMA10* (V-type ATPase) associated with the ion transportation process were downregulated, with *p*-values of 0.951 and 0.652 respectively. Collectively, *B. atrophaeus* 100MTN1 treatment resulted in differential expression across multiple biological process categories, with fold changes ranging from −1.45 to 3.86 across the various functional gene groups.

#### 3.6.2. GO Analysis of Molecular Functions

**Figure 4:**
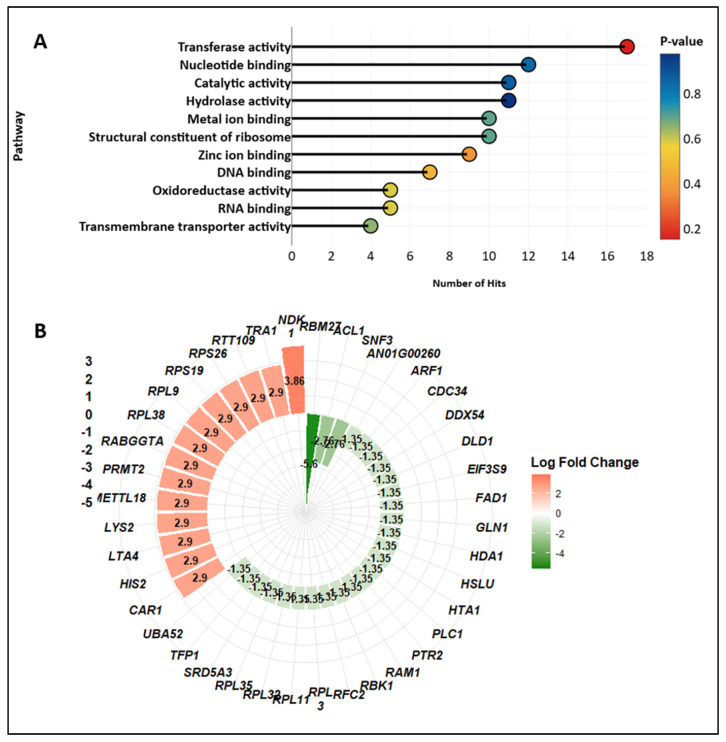
(**A**) The effect of *B. atrophaeus* 100MTN1 towards alterations in the number of genes linked to molecular functions of *Fol* FOLViF is depicted using a bar chart in comparison to the untreated control. The number of genes engaged in each pathway is displayed on the x-axis. The highest enrichment (17 genes) is noticed in transferase activity, followed by hydrolase and catalytic activity (11 genes). (**B**) Fold changes of transcripts associated with molecular functions in *Fol* mycelium antagonized by *B. atrophaues* 100MTN1.

Gene Ontology analysis of molecular functions revealed alterations in diverse enzymatic and binding activities as a consequence of *B. atrophaeus* 100MTN1 treatment, with a rigorous impact on fungal survival (Table 3, Figure 4). Among the various processes under molecular function, the major genes altered were contributing towards transferase activity with 17 differentially expressed genes (*p*-Value: 0.152). Key genes that were downregulated for the transferase activity include *CDC34*, *RBK1*, *RAM1*, *NEUTE1DRAFT_75477*, *RIB4* and *ALG1*. However, genes such as *NDK1*, *PRMT2*, *TRA1* and *METTL18* were upregulated. Genes corresponding to Zinc ion binding and DNA binding functions indicated an extensive modulation with nine and seven genes, which were differentially expressed respectively. Genes pertaining to *RAM1* were downregulated, and *LTA4* was upregulated in the zinc ion binding functions. While in DNA binding functions, the genes *HTA1*, *RFC2*, and *AN01G00260* were downregulated. The antagonistic action imposed by *B. atrophaeus* 100MTN1 against *Fol* FOLViF may have disrupted the ribosomal machinery by irregular expression of its structural subunits. The genes associated with larger ribosomal subunit components, including *RPL32*, *RPL3*, *RPL11*, and *RPL35*, were downregulated. However, the genes with smaller subunits, including *RPS26*, *RPL9*, *RPS19* and RPL38, were upregulated. Three genes, *AN01G00680* (iron-binding cytochrome b5), *SRD5A3* and *DLD1*, with a *p*-Value of 0.585, were downregulated in the oxido reductase activity of *Fol* FOLViF when antagonized by *B. atrophaeus* 100MTN1 and thus may have negatively regulated the ability to break down complex organic matter. Ten genes with a *p*-Value of 0.695 were altered in metal ion binding activity. Among those 10 genes, *AN01G00680*, *RBM27*, *ACL1*, *RAM1*, *RBK1* and *AN01G00260* were downregulated, while *AN11G00810*, *AN04400500*, *LTA4* and *CAR1* were upregulated. Similarly, the genes *LAT4* and *CAR1* were upregulated in the transmembrane transporter activity of *Fol* FOLViF. The RNA binding proteins were affected in four of five genes *AGABI1DRAFT_112564*, *RBM27*, *DDX54*, and *EIF3S9* with a *p*-Value of 0.585.

#### 3.6.3. GO Analysis of Cellular Component

**Figure 5:**
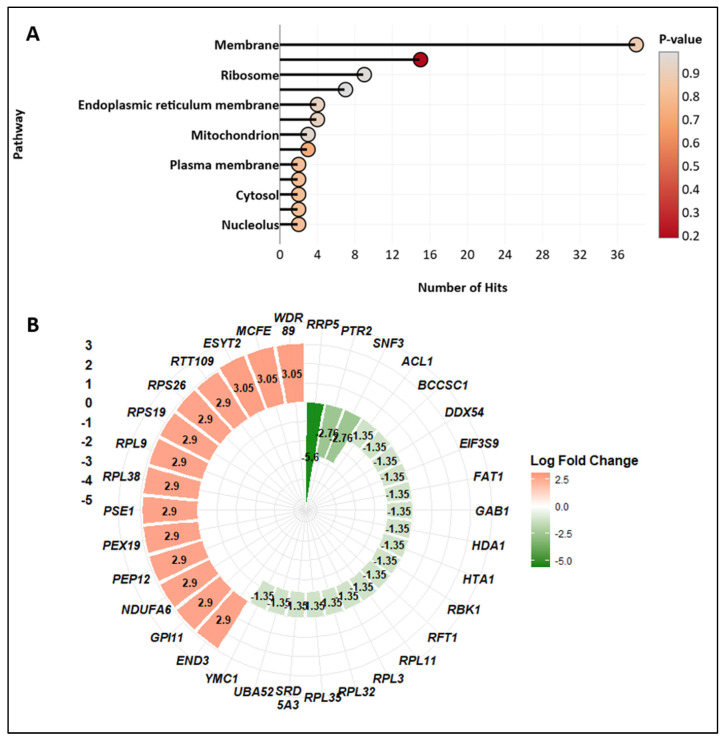
(**A**) The effect of *B. atrophaeus* 100MTN1 towards alterations in the number of genes linked to the cellular component of *Fol* FOLViF is depicted using a bar chart in comparison to the untreated control. The number of genes engaged in each pathway is displayed on the x-axis. The highest enrichment (38 genes) is seen in membrane components, followed by nucleus genes (15 hits). (**B**) Fold changes of transcripts associated with cellular components in *Fol* FOLViF mycelium antagonized by *B. atrophaeus* 100MTN1.

Gene Ontology analysis of cellular components revealed a substantial remodeling of cellular architecture and organelle function in response to interaction with *B. atrophaeus* 100MTN1. The highest number of differentially expressed genes (38 genes, *p* = 0.881) was from the membrane category, pertaining to genes *PTR2*, *END3*, *MCFE*, *PEP12*, *RFT1*, *GAB1*, *BCCSC1*, *AN01G00680*, *SNF3*, and *AN08G03710* exhibiting downregulation (Table 4, Figure 5). While genes such as *YMC1*, *NDUFA6*, *GPI11*, *AN01G02460*, *AN02G11950*, *AN02G02480*, and *ESYT2* were upregulated. Nuclear components comprised 15 genes (*p* = 0.191) among which *HTA1*, *UBA52*, *DDX54*, *HDA1*, and *RRP5* were downregulated, while *PSE1*, *RTT109*, *WDR89*, and *AN05G00610* were upregulated. Within the peroxisome category, *FAT1* showed downregulation while *PEX19* was upregulated (*p* = 0.809). The mitochondrion category showed three differentially expressed genes (*p* = 0.949), where *NDUFA6* and *AN11G11290* were downregulated, but *AGABI1DRAFT_99593* was upregulated. Cytoplasm components were also downregulated (7 genes, *p* = 0.47), with *UBA52*, *ACL1*, *RBK1*, and *EIF3S9* genes being downregulated. However, the genes *END3*, *PSE1*, and *18.M06140* were upregulated. Additionally, the cytosol component (*p* = 0.809) gene *ACL1* was also downregulated, while the gene *WDR89* was upregulated.

### 3.7. KEGG-Based Functional Pathway Assay

The application of KEGG functional pathway analysis provides a comprehensive view of how an organism dynamically responds to environmental stress. In this study, upregulated and downregulated genes were systematically mapped to their corresponding metabolic and signaling pathways to clarify the underlying biological mechanisms. The major group of genes downregulated were under metabolic pathway (map01100) followed by biosynthesis of ribosome (map03010) (Figure S7), biosynthesis of amino acid (map01230) (Figure S8), oxidative phosphorylation (map00190) (Figure S9), endocytosis (map04144) (Figure S10), riboflavin metabolism (map00740) (Figure S11), phagosome (map04145) (Figure S12), carbon metabolism (map01200) (Figure S13), pyruvate metabolism (map00620) (Figure S14), inositol phosphate metabolism (map00562) (Figure S15), pentose phosphate pathway (map00030) (Figure S16), ubiquitin mediated proteolysis (map04120) (Figure S17), terpenoid backbone biosynthesis (map00900) (Figure S18), RNA degradation (map03018) (Figure S19), nucleotide excision repair (map03420) (Figure S20), DNA replication (map03030) (Figure S21), mismatch repair (map03430) (Figure S22), glycolysis/gluconeogenesis (map00010) (Figure S23), nitrogen metabolism (map00910) (Figure S24). On the other hand, peroxisome activity was found to be upregulated (map04146) (Figure S25).

### 3.8. Protein Model Validation and Molecular Docking

The quality of the modeled structures was evaluated using the Ramachandran plot generated through the RamPlot server. The Grid size varied according to the different binding sites of the target protein. Details on the same are given in Table S5 and Figure S26.

**Figure 6:**
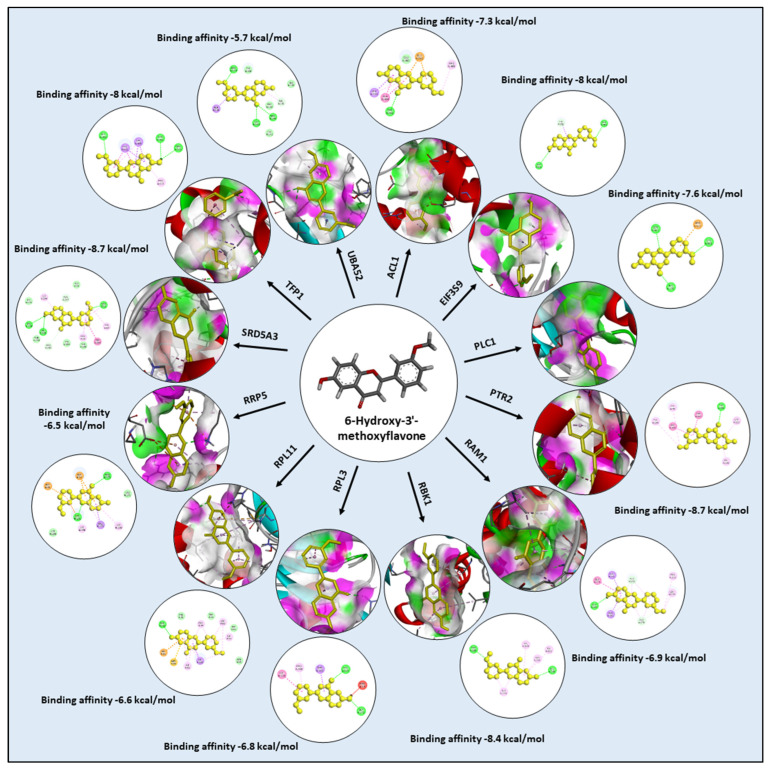
Molecular modeling display illustrating the docking complexes of the biomolecule 6-Hydroxy-3′-methoxyflavone bound to the predicted protein structures of downregulated genes in *Fol* FOLViF antagonized by *B. atrophaeus* 100MTN1. The graphic highlights the spatial orientation, hydrogen bonding, and hydrophobic pockets within the binding domains. [Note: The docking images were generated through BIOVIA Discovery Studio, version: V25.1.0.24284].

The molecular docking study of the biomolecules extracted from the zone of inhibition showed that biomolecules, such as 6-Hydroxy-3′-methoxyflavone from *B. atrophaeus* 100MTN1, have a very strong affinity for the protein target *Fol* FOLViF. These biomolecules exhibited exceptional binding affinity when compared to positive control, carbendazim, tebuconazole and trifloxystrobin (Figure 6).

**Figure 7:**
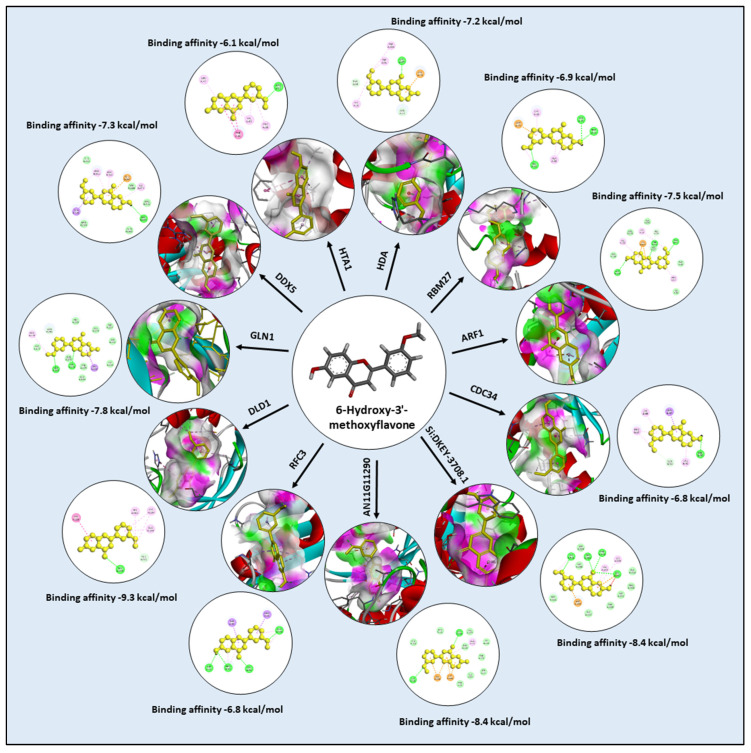
Multi-panel molecular modeling graphic illustrating the binding interactions between the ligand 6-Hydroxy-3′-methoxyflavone and receptor proteins corresponding to downregulated genes in *Fol* FOLViF under *B. atrophaeus* 100MTN1 antagonism. The layout highlights the active site architecture, rendering detailed views of hydrogen bonds and hydrophobic interactions. [Note: The image was generated through BIO VIA discovery studio, version: V25.1.0.24284].

**Figure 8:**
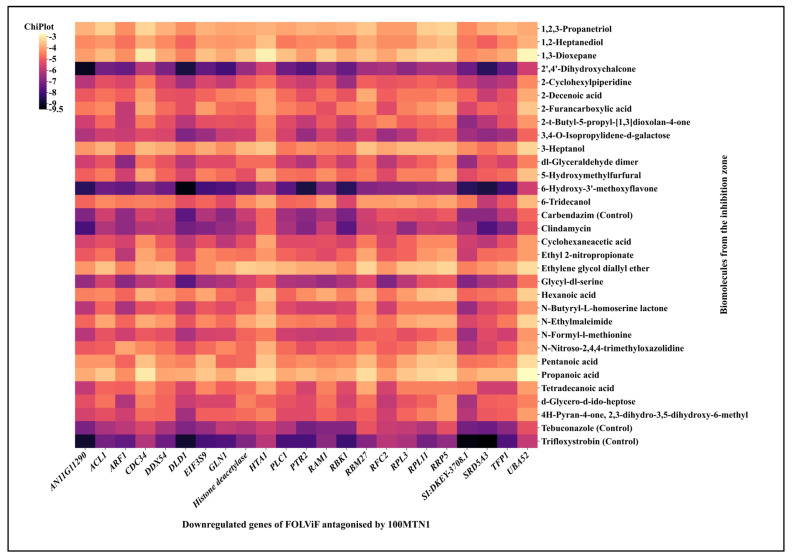
Transcriptomic and molecular docking heatmap explaining the binding affinity matrix between biomolecules extracted from the inhibition zone of a dual assay between *Fol* FOLViF and *B. atrophaeus* 100MTN1 and the receptor proteins associated with the downregulated genes of the *Fol* FOLViF antagonized by *B. atrophaeus* 100MTN1. The color gradients represent varying degrees of binding energy values across the dataset. [Note: The image was generated through https://www.chiplot.online/ accessed on 28 March 2026].

6-Hydroxy-3′-methoxyflavone exhibited a binding affinity of −9.3 kcal/mol with DLD1 (H-bond; GLY 220), which was the highest among all the targets (Figure 7). It had a binding affinity of −8.7 kcal/mol with PTR2 (H-bond; TYR 258) and SRD5A3 (H-bond; ARG 52, LEU 231, LEU 234) respectively. With AN11G11290 (H-bond; LYS 231, ASN 249), RBK1 (H-bond; THR 239, LEU 260) and SI: DKEY-3708.1 (H-bond; LYS 287, SER 288, THR 289, LYS 421), was witnessed with the binding affinity of −8.4 kcal/mol. A binding affinity of −8 kcal/mol was obtained with the interaction of 6-Hydroxy-3′-methoxyflavone with the EIF3S9 (H-bond; GLN 461, ASP 521) and TFP1 (H-bond; GLN 408, GLY 432, GLN 699) proteins. Similarly, the binding affinity of −7.8 kcal/mol, −7.6 kcal/mol and −7.5 kcal/mol was observed for 6-Hydroxy-3′-methoxyflavone while interacting with GLN1 (H-bond; SER 161, VAL 176), PLC1 (H-bond; HIS 178, LYS 401, SER 431) and ARF1 (H-bond; ASN 27, GLY 29, THR 31, THR 32, ASP 129) respectively. The interaction of 6-Hydroxy-3′-methoxyflavone with ACL1 (H-bond; THR 488) and DDX54 (H-bond; ASP 420) was recorded to be −7.3 kcal/mol. The binding affinity of 6-Hydroxy-3′-methoxyflavone of RAM1 (H-bond; GLY 369, ALA 372, ALA 378) and RBM27 (H-bond; TRP 14, SER 78, ILE 89) was found to be −6.9 kcal/mol. A binding affinity for the same molecule was observed as −6.8 kcal/mol with the target genes CDC34 (H-bond; VAL 70, GLN 93), RFC1 (H-bond; LEU 54, ASP 55, ARG 99, ALA 103) and RPL3 (H-bond; HIS 225, GLY 272). The observed binding affinity of 6-Hydroxy-3′-methoxyflavone with HDA1 (Histone deacetylase) (H-bond; LYS 97), RPL11 (H-bond; THR 125), RRP5 (H-bond; ARG 275, LYS 291), HTA1 (H-bond; ARG 42) and UBA52 (H-bond; ARG 119, VAL 131, GLY 143, ARG 144) was −7.2 kcal/mol, −6.6 kcal/mol, −6.5 kcal/mol, −6.1 kcal/mol, −5.7 kcal/mol (Figure 8).

## 4. Discussion

Numerous studies have shown that beneficial microorganisms use direct and indirect mechanisms to boost crop health, nutritional quality, and stress tolerance [23,24,25,26,27]. It is widely established that an array of plant growth-promoting bacteria (PGPB) and *Rhizobium* species enhance plant growth and development through the secretion of phytohormones, including auxins, cytokinins and gibberellins [28,29,30]. Additionally, these microorganisms produce antifungal compounds including phenazines, pyrrolnitrin, DAPG, pyoluteorin, viscosinamide, and tensin. These metabolites help minimize plant diseases by directly inhibiting the pathogens [31]. Moreover, certain beneficial microorganisms secrete lytic enzymes capable of degrading the cell walls of pathogens. This mechanism is exemplified by *Lysobacter*, which synthesizes chitinase, β-1, 3-glucanases, and proteases, as well as *Bacillus* sp. KTMA4, which generates amylase, cellulase, xylanase, and lipase [31,32]. Furthermore, beneficial microorganisms can stimulate induced systemic resistance (ISR), providing plants with comprehensive defense against multiple pathogens [33,34]. *Bacillus* species exhibit strong environmental resilience with their characteristic hardy spores capable of enduring unfavorable conditions for extended periods [35]. *Trichoderma* demonstrates the capacity to flourish in extreme environments and quickly establish itself within the plant rhizosphere [36]. Of the diverse array of plant-beneficial microorganisms, *Bacillus* represents the most thoroughly investigated and commonly utilized taxon, which successfully stimulates plant development and provides protection against a wide range of soil-borne pathogens [35,36,37].

In the present investigation, 20 bacterial isolates derived from cured SCB were evaluated for their ability to inhibit *Fol*, the causal agent of tomato wilt. Among these, *B. atrophaeus* 100MTN1 exhibited the strongest antifungal activity, outperforming all other isolates in both *in vitro* and *in vivo* assays against *Fol* FOLViF. Previous studies revealed that certain bacteria can suppress the *Fol* FOLViF, like *B. subtilis* Bs-06, which inhibited fungal growth by 70% [38]. The radial mycelial growth of *Fol* FOLViF was significantly suppressed by two bacterial antagonists, *B. subtilis* BsTA16 and *Acinetobacter calcoaceticus* AcDB3, with inhibition rates of 44.63% and 27.39%, respectively [39]. However, the potential of *B. atrophaeus* has not been explored widely. Our findings establish that *B. atrophaeus* 100MTN1 is an effective antagonist against tomato (cv. Kalyan) wilt pathogenesis, thereby addressing this gap in the current understanding of biocontrol agents and plant defense mechanisms.

Our transcriptome profiling data of *F. oxysporum* f. sp. *lycopersici* (*Fol*) FOLViF under direct niche influence of *B. atrophaeus* 100MTN1 revealed a sophisticated, multipronged antifungal strategy rather than a single mode of action. The observed gene expression patterns demonstrated that *B. atrophaeus* 100MTN1 simultaneously might have affected multiple critical systems, including energy metabolism, redox homeostasis, membrane integrity, protein translation, and cellular signaling. By modulating these diverse physiological pathways, the bacterium imposes a metabolic barrier that is unlikely to be overcome by adaptive mutations in the pathogen. This broad-spectrum inhibitory approach resembled the efficacy of modern synthetic antifungals that act through multi-target mechanisms, suggesting that *B. atrophaeus* 100MTN1 produces groups of bioactive compounds capable of comprehensive fungal suppression.

Moreover, membrane integrity and transport dysfunction revealed that the most prominent transcriptional changes were associated with membrane structure and transport, with thirty-eight membrane-associated genes being altered. The downregulation of the mitochondrial carrier protein YMC1 and major facilitator superfamily (MFS) transporters (PTR2) points to a profound impairment in both nutrient acquisition and toxin efflux pathways. This disruption of transport systems and the cellular damage observed in *Fol* FOLViF exposed to the establishment of a possible membrane targeting antifungal mechanism. For instance, *F. graminearum* treated with thymol exhibited lipid peroxidation, ergosterol depletion, and eventual cell lysis [40]. In a similar fashion, the suppression of *PTR2* by *B. atrophaeus* 100MTN1 likely induced a metabolic bottleneck by blocking the uptake of small peptides, which are vital alternative sources of nitrogen and carbon [41], thereby indirectly delaying protein synthesis. *PTR2* inhibition in *F. graminearum* showed that mutations in the FgPTR2 gene family severely hindered sporulation and the formation of perithecia, thereby reducing the pathogenic virulence. These metabolic changes were increased by the simultaneous repression of YMC1, which may have compromised the mitochondrial transport and disruption of intra-cellular signaling pathway. Collectively, these data suggested that *B. atrophaeus* 100MTN1 might have employed a dual membrane targeting strategy, where it physically destabilizes the lipid bilayer while concurrently suppressing the expression of essential transporters, induces *Fol* FOLViF into an acute nutrient deprivation stage, which leads to toxic metabolite accumulation.

During protein synthesis, ribosomal imbalance and translational disruption may have caused stress-induced failure in *Fol* FOLViF, characterized by a discordant expression pattern among ribosomal protein genes such as the downregulation of the large subunit components; *RPL32*, *RPL11*, *RPL35* and *RPL3*, alongside the upregulation of *RPS38*, *RPL9*, as well as small subunit components including *RPS19* and *RPS26*. Such irregular expression of proteins in ribosomal subunits might suggest a fundamental failure in ribosomal assembly and stoichiometry, which likely results in a moderated capacity for protein synthesis. The imbalance is linked to *RRP5*, a critical nucleolar scaffold required for the maturation of both the 18S and 5.8S rRNA subunits [20]. These protein deficiencies prevent the synchronized production of ribosomal subunits, thereby delaying the whole translational system [42]. Consequently, the erratic expression of these genes in *Fol* FOLViF likely triggers ribosomal stress, which severely restricts protein production and impairs cellular development. The physiological collapse might be directly linked to the pathogen’s compromised virulence and its inability to survive under bioactive compounds released during bacterial antagonism. Furthermore, the repression of *SRD5A3* and *RFT1* genes central to N-linked protein glycosylation points towards the activation of endoplasmic reticulum (ER) stress, as glycosylation disruption leads to morphological abnormalities and attenuated virulence and a sharp decline in pathogenicity [43]. Consequently, *B. atrophaeus* 100MTN1 interferes with multiple pathways involved in energy production and protein maturation mechanisms, signifying that *B. atrophaeus* 100MTN1 may exploit a multilayered antifungal approach that effectively disrupts both cellular energy and protein maturation.

*B. atrophaeus* 100MTN1 exposure triggers a severe redox imbalance within *Fol* FOLViF by suppressing oxidoreductases through downregulation of enzymes like *DLD1* (D-lactate dehydrogenase 1) and prevents the pathogen from activating the detoxifying reactive metabolites, which lead to impairment in mitochondrial energy generation [44]. The loss in oxidative equilibrium likely resulted in the toxic build-up of reactive oxygen species (ROS), which can create damage to essential proteins, lipids, and nucleic acids. The *DLD1* mutants have already been reported to induce avirulence in species like *Magnaporthe oryzae* [45], suggesting that *B. atrophaeus* 100MTN1 may also directly destabilize these pathways in *Fol* FOLViF. The downregulation of genes like *TFP1* and *VMA10*, essential subunits of the vacuolar H^+^-ATPase (V-ATPase) complex, may have been responsible for the failure in pH regulation and ion homeostasis. Since the V-ATPase generates electrochemical proton gradients required for maintaining cytoplasmic pH, driving secondary transport, and sequestering cations [46], suppression of these *TFP1* subunits suggest that *B. atrophaeus* 100MTN1 may lead to cytoplasmic alkalization and vacuolar dysfunction, with similar defects in mutants of *Candida albicans*, *Saccharomyces cerevisiae*, and *Histoplasma capsulatum*, indicating a possible cause of iron starvation, compromised membrane integrity, stunted growth and reduced virulence [47,48,49].

*PLC1* downregulation, encoding phosphatidylinositol-specific phospholipase C, indicates disruption of phospholipid signaling pathways essential for stress resistance, virulence and growth of *Fol* FOLViF. In *Alternaria alternata*, Huang et al. 2022 [50] showed that ΔAaPLC1 mutants showed a decrease in appressorium formation and invasive hyphae on dewaxed onion epidermis by 14.5 and 65.7% after 8 h incubation respectively. Moreover, they observed that loss of function of *PLC1* displays pleiotropic defects with severe impairing biological functions, including growth retardation, altered environmental stress tolerance, and a marked decline in host pathogenicity. In *F. graminearum*, deletion of FgPLC1 impairs hyphal growth, conidia production, and deoxynivalenol (DON) synthesis, with a marked reduction in wheat spike virulence [51]. Similarly, repression of *ACL1* (ATP citrate lyase) likely depletes cytosolic acetyl-CoA, a critical precursor for ergosterol and fatty acid biosynthesis [21]. These transcriptional shifts indicate that *B. atrophaeus* 100MTN1 may induce sterol imbalance and compromise membrane remodeling capacity, consistent with the effects of known antifungal agents that target ergosterol synthesis.

Further, profound changes were observed in nitrogen metabolism-governed genes, marked by substantial shifts in nitrogen assimilation and biosynthetic pathways. Downregulation of *GLN1* (glutamine synthetase) blocks ammonia assimilation and arginine synthesis, inducing a state of nitrogen starvation [52]. This metabolic blockade could have induced the arginine auxotrophy and a state of nitrogen starvation, triggering the upregulation of *CAR1* as a compensatory stress response. Additionally, the repression of *ENO1* may obstruct RNA degradation processes, potentially resulting in the toxic accumulation of misfolded RNAs and the activation of proteotoxic stress pathways [53]. The high expression of *HIS2*, which encodes a bifunctional enzyme involved in histidine biosynthesis, indicated a potential compensatory response that influences cell wall structure. Enhanced expression of *HIS2* has been associated with increased chitin and glycan accumulation, leading to alterations in cell wall composition [54]. These observations suggest a coordinated reconfiguration of amino acid metabolism that enables the organism to cope with nutrient limitation while simultaneously reinforcing its structural integrity. The structural integrity and vesicular trafficking systems of *Fol* FOLViF may have been compromised during this antagonistic interaction. Specifically, disruption of *ARF2* (ADP-ribosylation factor 2) impairs Golgi function and clathrin-coated vesicle assembly, preventing recycling of cell wall biosynthetic enzymes [55]. Simultaneously, the suppression of RAM1, encoding the β-subunit of farnesyltransferase, stalls prenylation of Ras and Rho GTPases, key regulators of signal transduction and morphology [56]. Furthermore, concurrent downregulation of *CDC34*, *RFC2*, *RBK1*, and *ARF1* indicates simultaneous failure in DNA replication, cellular proliferation, as well as in ubiquitin-mediated proteolysis and DNA replication. *CDC34* inhibition leads to G1 phase arrest [57], while *RFC2* repression impairs mismatch repair and genome duplication [58,59]. Collectively, these transcriptomic shifts indicated that the antagonism of *B. atrophaeus* 100MTN1 might have enforced a genetic blockade on the cell cycle, leading to the total inhibition of growth and the possible onset of apoptosis in *Fol* FOLViF.

The tentatively identified metabolites from the inhibition zone during the co-culturing of *B. atrophaeus* 100MTN1 and *Fol* FOLViF consist of 31 bioactive compounds. The strong antagonism observed *in vitro* resulted from the synergistic interaction of these biomolecules, which may have caused the suppression of radial growth of the pathogen. Propionic acid (10%) has been shown to inhibit the mycelial growth of *Rhizopus nigricans* by 26.57% [60]. While Propionic acid at 0.7% completely inhibited the mycelial growth of *Macrophomina phaseolina*, *Botrytis cinerea*, *Sclerotinia sclerotiorum*, *Fusarium oxysporum* and *Rhizoctonia solani* [61]. Arundathi and Ramesh confirmed the antifungal activity of Hexanoic acid against *Fusarium oxysporum*. Among the different concentrations, the maximum mycelial growth inhibition of 65.7% was recorded in 0.2% [62]. The methanolic peel extract of *Punica granatum* (4H-Pyran-4-one, 2, 3-dihydro-3, 5-dihydroxy-6-methyl being one of them) expressed strong antimicrobial activity against *Aspergillus fumigatus*. A broad spectrum antimicrobial activity of 4H-Pyran-4-one, 2, 3-dihydro-3, 5-dihydroxy-6-methyl was shown against *Proteus mirabilis*, *Pseudomonas aerogenosa*, *Escherichia coli*, *Staphylococcus aureus* and *Klebsiella pneumonia* through agar well diffusion method [63]. In 2007, Jung and his coworkers demonstrated the broad spectrum antifungal effect of 5-Hydroxymethylfurfural against *Alternaria mali*, *Valsa ceratosperma*, *Glomerella cingulate*, *Phomopsis mali* and *Botrytis cinerea*, causing Alternaria leaf spot, Valsa canker, Bitter rot, Die-back and Gray mold respectively [64]. In a research carried out by Wu and his colleagues, 2-Heptanol was recorded to suppress the growth of *B. cinerea* mycelia both in vitro and in vivo by stimulating the activities of antioxidative enzymes, including superoxide dismutase (SOD), peroxidase (POD) and catalase (CAT) in tomatoes. Furthermore, it reduced the spore viability, compromised membrane integrity and resulted in increased levels of extracellular nucleic acid, protein content and membrane lipid peroxidase [65]. Among the bioactive compounds, the presence of n-hexadecanoic acid (palmitic acid), tetradecanoic acid (myristic acid), and 5-hydroxymethylfurfural are known to disrupt membrane integrity, induce oxidative stress, suppress chitin synthase (ChsV), folate uptake block gene (FUBT) and fusaric acid biosynthesis [66,67]. These compounds are potent inhibitors of fungal respiration and spore germination. Detection of Hexanoic acid in fungal mycelia has been shown to suppress spore germination and mycelial expansion in *Botrytis cinerea* [68], suggesting a conserved mechanism across fungal pathogens. Molecular docking analysis revealed that the presumptively identified bioactive compounds via GC-MS may have inhibited various essential fungal target proteins. Among the expressed biomolecules, 6-Hydroxy-3′-methoxyflavone exhibited strong negative binding affinity against virulent proteins essential for *Fol* FOLViF, comparable to that of the commercial fungicides like carbendazim, tebuconazole, and trifloxystrobin, highlighting its possible antifungal activity against the pathogen.

The functional relevance of these transcriptomic and metabolomic findings was confirmed in greenhouse trials. The significant suppression of disease observed in tomato plants (cv. Kalyan) treated with *B. atrophaeus* 100MTN1 + *Fol* FOLViF under greenhouse trials, marked by a 79.79% reduction in disease incidence (17.97%) compared with untreated controls (88.87%). The antagonistic activity by *B. atrophaeus* 100MTN1 on *Fol* FOLViF *in vitro* directly correlates with the observed reduction in its ability to infect the host under agronomic conditions. Collectively, our findings demonstrated that *B. atrophaeus* 100MTN1 might be a promising biological control agent against Fusarium wilt of tomato (cv. Kalyan).

This mechanistic complexity suggests that resistance development by the pathogen is unlikely, placing *B. atrophaeus* 100MTN1 as a durable and environmentally sustainable alternative to chemical fungicides. However, we strongly recommend that future work should focus on mutant-based validation of important genes, formulation optimization, field validation across diverse agronomic settings, and exploration of the novel bioactive compounds identified in this study via GC-MS, like 6-Hydroxy-3′-methoxyflavone and related compounds, as potential templates for synthetic antifungal design.

## 5. Conclusions

This study demonstrates that sugarcane bagasse curing is a viable source of bacterial antagonists for sustainable crop protection. Specifically, the strain *B. atrophaeus* 100MTN1 proved highly effective in suppressing *Fol* FOLViF by employing a dual mechanism involving the secretion of inhibitory bioactive metabolites and transcriptional disruption of essential pathogen pathways. By integrating metabolomic, transcriptomic, and molecular docking analyses, we provided a comprehensive and robust explanation of the mode of action of 100MTN1. Furthermore, *B. atrophaeus* 100MTN1 showed significant success in glasshouse conditions, where it not only lowered the severity of Fusarium wilt but also enhanced the overall growth of tomato plants (cv. Kalyan). These findings indicate that the strain is a prime candidate for bio-based crop protection and could eventually serve as an environmentally sustainable alternative to chemical fungicides. To bring this technology to market, future research should focus on optimizing the product formulation, validating its performance under field conditions, and conducting a biosafety assessment to support commercial application.

## Figures and Tables

**Table 1:** Efficacy of *B. atrophaeus* 100MTN1 on plant growth promotion and wilt incidence of Fusarium wilt in tomato (cv. Kalyan) under glasshouse conditions. * Values are the mean of three replications. The values in parentheses are arc sine converted values, and the means in a column that are preceded by a similar letter do not differ substantially at the 5% level of DMRT.

Treatments	Treatment Details	Plant Height			% Wilt Incidence *	% Reduction over Control
		30 DPI	60 DPI	90 DPI		
T1	*Fol* FOLViF + *B. velezensis* VB7	34.8 ^b^	64.5 ^b^	93.9 ^b^	24.07 ^b^ (29.38)	72.04
T2	*Fol* FOLViF + *B. tequilensis* NB LEAF 6	32.2 ^c^	63.2 ^c^	91.3 ^c^	28.57 ^c^ (32.31)	66.82
T3	*Fol* FOLViF + *B. atrophaeus* 100MTN1	37.4 ^a^	68.4 ^a^	95.4 ^a^	18.88 ^a^ (25.75)	78.07
T4	*Fol* FOLViF + *B. glycinifermentans* CNEB17	30.2 ^d^	62.0 ^d^	90.1 ^d^	33.13 ^d^ (35.14)	61.52
T5	*Fol* FOLViF + *B. subtilis* IBHB4	29.8 ^e^	60.6 ^e^	83.2 ^e^	36.11 ^d^ (36.94)	58.07
T6	Inoculated control (*Fol* FOLViF)	26.7 ^f^	50.8 ^g^	64.1 ^g^	86.11 ^e^ (68.12)	0.00
T7	Healthy control	28.1 ^e^	56.0 ^f^	77.8 ^g^	0	100.00
SE (d)		0.475	0.456	0.437	1.329	
CD (0.05%)		1.029	0.947	0.907	2.993	

**Table 2:** A functional enrichment chart plotting the transcriptomic changes associated with the biological processes of *Fol* FOLViF mycelium during interaction with *B. atrophaeus* 100MTN1. The data contrast the specific counts of upregulated and downregulated genes against the untreated control group to reveal shifting biological pathways.

Biological Process	GO Accession	Hits	*p*-Value	AdjP	Representative Genes	Fold Changes
Translation	GO:0006412	12	0.616	0.954	*RPS26*, *RPL9*, *RPL38*, *RPS19*, *RPL32*, *UBA52*, *RPL3*, *RPL11*, *RPL35*, *SI:DKEY-37O8.1*, *18.M06140*, *EIF3S9*	−1.35 to 2.90
Transmembrane transport	GO:0055085	7	0.951	0.954	*PTR2*, *s2f_0000821012*, *AGABI1DRAFT_108367*, *AN02G02480*, *AGABI1DRAFT_127160*, *SNF3*, *AN04G07510*	−2.76 to 2.90
Proteolysis	GO:0006508	4	0.92	1	*LTA4*, *AGABI1DRAFT_37296*, *AGABI1DRAFT_36466*, *HSLU*	−1.35 to 2.90
Phosphorylation	GO:0016310	5	0.887	1	*NDK1*, *TRA1*, *AN02G13590*, *RBK1*, *s2f_0002860013*	−1.35 to 3.86
Transcription	GO:0006351	4	0.652	0.954	*AN11G00810*, *AN04G00500*, *AN16G08130*, *AN01G00260*	2.90
Ion transport	GO:0006811	4	0.652	0.954	*BCCSC1*, *TFP1*, *AGABI1DRAFT_127160*, *VMA10*	−1.35
Methylation	GO:0032259	4	0.652	0.954	*PRMT2*, *METTL18*, *AGABI1DRAFT_112491*, *NEUTE1DRAFT_75477*	−1.35 to 2.90
Carbohydrate metabolic process	GO:0005975	3	0.726	0.954	*AGABI1DRAFT_69432*, *RBK1*, *AN11G03120*	−1.35 to 2.90
MRNA processing	GO:0006397	3	0.726	0.954	*RRP5*, *RBM27*, *AN02G01220*	−5.60 to 2.90
Lipid metabolic process	GO:0006629	3	0.726	0.954	*PLC1*, *ACL1*, *SRD5A3*	−1.35

**Table 3:** A transcriptomic data table labeled comparing the profiles of DEGs in the mycelium of *Fol* FOLViF. The dataset contrasts the untreated control group against the treatment group antagonized by *B. atrophaeus* 100MTN1 to highlight significant changes in gene regulation.

Molecular Function	GO Accession	Hits	*p*-Value	AdjP	Representative Genes	Fold Changes
Transferase activity	GO:0016740	17	0.152	0.974	*NDK1*, *PRMT2*, *AN01G06530*, *AGABI1DRAFT_69432*, *TRA1*, *METTL18*, *RABGGTA*, *AGABI1DRAFT_112491*, *RTT109*, *ACL1*, *AGABI1DRAFT_126370*, *CDC34*, *RAM1*, *RBK1*, *NEUTE1DRAFT_75477*, *RIB4*, *ALG1*	−2.76 to 3.86
Zinc ion binding	GO:0008270	9	0.377	0.974	*LTA4*, *AN11G00810*, *AN04G00500*, *AN05G00610*, *AN16G08130*, *s2f_0030177001*, *AGABI1DRAFT_125365*, *RAM1*, *AN01G00260*	−2.76 to 2.90
DNA binding	GO:0003677	7	0.47	0.974	*AN11G00810*, *AN04G00500*, *AN16G08130*, *HTA1*, *AGABI1DRAFT_36466*, *RFC2*, *AN01G00260*	−2.76 to 2.90
RNA binding	GO:0003723	5	0.585	0.974	*AGABI1DRAFT_112564*, *RPS19*, *RBM27*, *DDX54*, *EIF3S9*	−5.60 to 3.86
Oxidoreductase activity	GO:0016491	5	0.585	0.974	*AGABI1DRAFT_114383*, *12.T00050*, *AN01G00680*, *SRD5A3*, *DLD1*	−5.60 to 3.86
Transmembrane transporter activity	GO:0022857	4	0.652	0.974	*PTR2*, *AN02G02480*, *SNF3*, *AN04G07510*	−2.76 to 2.90
Hydrolase activity	GO:0016787	11	0.655	0.974	*LTA4*, *CAR1*, *16.T00021*, *HIS2*, *DDX54*, *PLC1*, *HDA1*, *AGABI1DRAFT_37296*, *AGABI1DRAFT_36466*, *AN11G03120*, *SIT4*	−2.74 to 2.90
Structural constituent of ribosome	GO:0003735	10	0.695	0.974	*RPS26*, *RPL9*, *RPL38*, *RPS19*, *MRPL11*, *RPL32*, *UBA52*, *RPL3*, *RPL11*, *RPL35*	−1.35 to 2.90
Metal ion binding	GO:0046872	10	0.695	0.73	*LTA4*, *CAR1*, *AN11G00810*, *AN04G00500*, *AN01G00680*, *RBM27*, *ACL1*, *RAM1*, *RBK1*, *AN01G00260*	−5.60 to 2.90
Nucleotide binding	GO:0000166	12	0.847	0.73	*NDK1*, *TRA1*, *DDX54*, *ACL1*, *ARF1*, *SI:DKEY-37O8.1*, *CDC34*, *RFC2*, *RBK1*, *GLN1*, *HSLU*, *TFP1*	−1.35 to 3.86
Catalytic activity	GO:0003824	11	0.871	1	*ECH1*, *AGABI1DRAFT_107237*, *LYS2*, *AN01G06530*, *AN04G08900*, *HIS2*, *ACL1*, *FAD1*, *RAM1*, *GLN1*, *DLD1*	−1.35 to 2.90

**Table 4:** Gene regulation within the cellular component category for *Fol* FOLViF mycelium antagonized by *B. atrophaeus* 100MTN1. The graphic uses distinct categories to map upregulated and downregulated gene counts for various cellular structural targets compared to an untreated control.

Cellular Component	GO Accession	Hits	*p*-Value	AdjP	Representative Genes	Fold Changes
Nucleus	GO:0005634	15	0.191	0.99	*PSE1*, *16.T00021*, *AN11G00810*, *AN04G00500*, *RTT109*, *AN05G00610*, *RRP5*, *s2f_0030177001*, *UBA52*, *DDX54*, *HDA1*, *WDR89*, *HTA1*, *RBK1*, *AN01G00260*	−5.60 to 2.90
Cytoplasm	GO:0005737	7	0.47	0.99	*END3*, *PSE1*, *UBA52*, *ACL1*, *RBK1*, *18.M06140*, *EIF3S9*	−2.76 to 2.90
Mitochondrial inner membrane	GO:0005743	3	0.726	0.99	*NDUFA6*, *AGABI1DRAFT_99593*, *AN11G11290*	−1.35 to 2.90
Nucleolus	GO:0005730	2	0.809	0.99	*RRP5*, *DDX54*	−5.60 to −1.35
Peroxisome	GO:0005777	2	0.809	0.99	*PEX19*, *FAT1*	−1.35 to 2.90
Cytosol	GO:0005829	2	0.809	0.99	*ACL1*, *WDR89*	−1.35
Eukaryotic translation initiation factor 3 complex	GO:0005852	2	0.809	0.99	*18.M06140*, *EIF3S9*	−2.76 to 2.90
Plasma membrane	GO:0005886	2	0.809	0.99	*END3*, *s2f_0025820001*	2.90
Membrane	GO:0016020	38	0.881	0.99	*NEUTE1DRAFT_80497*, *PTR2*, *YMC1*, *END3*, *MCFE*, *s2f_0000821012*, *AGABI1DRAFT_108367*, *PEP12*, *NDUFA6*, *GPI11*, *AGABI1DRAFT_128372*, *NEUTE1DRAFT_99472*, *AGABI1DRAFT_113592*, *AGABI1DRAFT_99593*, *AGABI1DRAFT_108085*, *AN01G02460*, *AN02G11950*, *NEUTE1DRAFT_95859*, *s2f_0021425001*, *s2f_0048074001*, *s2f_0025820001*, *AN01G00680*, *BCCSC1*, *GAB1*, *AGABI1DRAFT_77080*, *ESYT2*, *AN02G02480*, *SRD5A3*, *RFT1*, *SNOG_04586*, *AGABI1DRAFT_127160*, *AN11G11290*, *AN08G03710*, *AN09G05600*, *NEUTE1DRAFT_149317*, *AN08G07460*, *SNF3*, *AN04G07510*	−2.76 to 2.90
Endoplasmic reticulum	GO:0005783	4	0.92	0.944	*AGABI1DRAFT_108085*, *FAT1*, *GAB1*, *SRD5A3*	−1.35
Endoplasmic reticulum membrane	GO:0005789	4	0.92	0.944	*GPI11*, *AGABI1DRAFT_108085*, *GAB1*, *SRD5A3*	−1.35 to 2.90
Mitochondrion	GO:0005739	3	0.949	0.99	*NDUFA6*, *AGABI1DRAFT_99593*, *AN11G11290*	−1.35 to 2.90
Ribosome	GO:0005840	9	0.978	1	*RPS26*, *RPL9*, *RPL38*, *RPS19*, *RPL32*, *UBA52*, *RPL3*, *RPL11*, *RPL35*	−1.35 to 2.90

## Data Availability

The data presented in this study are available within the article and the Supplementary Materials. The transcriptomic sequencing data have been deposited in the NCBI BioProject repository under accession number PRJNA1476957. The BioSample accessions associated with this project are SAMN60749111 (A1_R1), SAMN60749112 (A1_R2), SAMN60749113 (A1_R3), SAMN60749114 (C_R1), SAMN60749115 (C_R2), SAMN60749116 (C_R3), SAMN60749117 (H1_R1), SAMN60749118 (H1_R2), and SAMN60749119 (H1_R3). The datasets are currently under embargo in the NCBI database and will be released for public access upon publication of the article. Reviewer access or additional information can be obtained from the corresponding author upon reasonable request.

## Supplementary Materials

The following supporting information can be downloaded at: https://www.mdpi.com/article/10.3390/microorganisms14071488/s1.

## Abbreviations

The following abbreviations are used in this manuscript:

**Abbreviation**	**Full Form**
DEG	Differentially Expressed Gene
*Fol*	*Fusarium oxysporum* f. sp. *lycopersici*
GC–MS	Gas Chromatography–Mass Spectrometry
