# Supplementary material for: Interactions Between Bacillus atrophaeus 100 MTN1 and Fusarium oxysporum f. sp. lycopersici Reprogram the Transcriptomic and Metabolomic Profile to Combat Tomato (cv. Kalyan) Wilt
Source: Microorganisms. 2026 Jul 7;14(7):1488. doi: 10.3390/microorganisms14071488 (PMC13413564; doi:10.3390/microorganisms14071488)
Supplement: Supplementary file 1 [file microorganisms-14-01488-s001.zip › microorganisms-4362257-supplementary.pdf]

Figure S1. Dual confrontation assay between antagonistic bacteria isolated from SCB against *Fol* FOLViF. Note: The assay between the antagonistic bacteria and *Fol* FOLViF was evaluated by co-culturing in PDA media, in the absence of bacterial antibiotics. The picture was taken 8 days post initiation of the experiment.

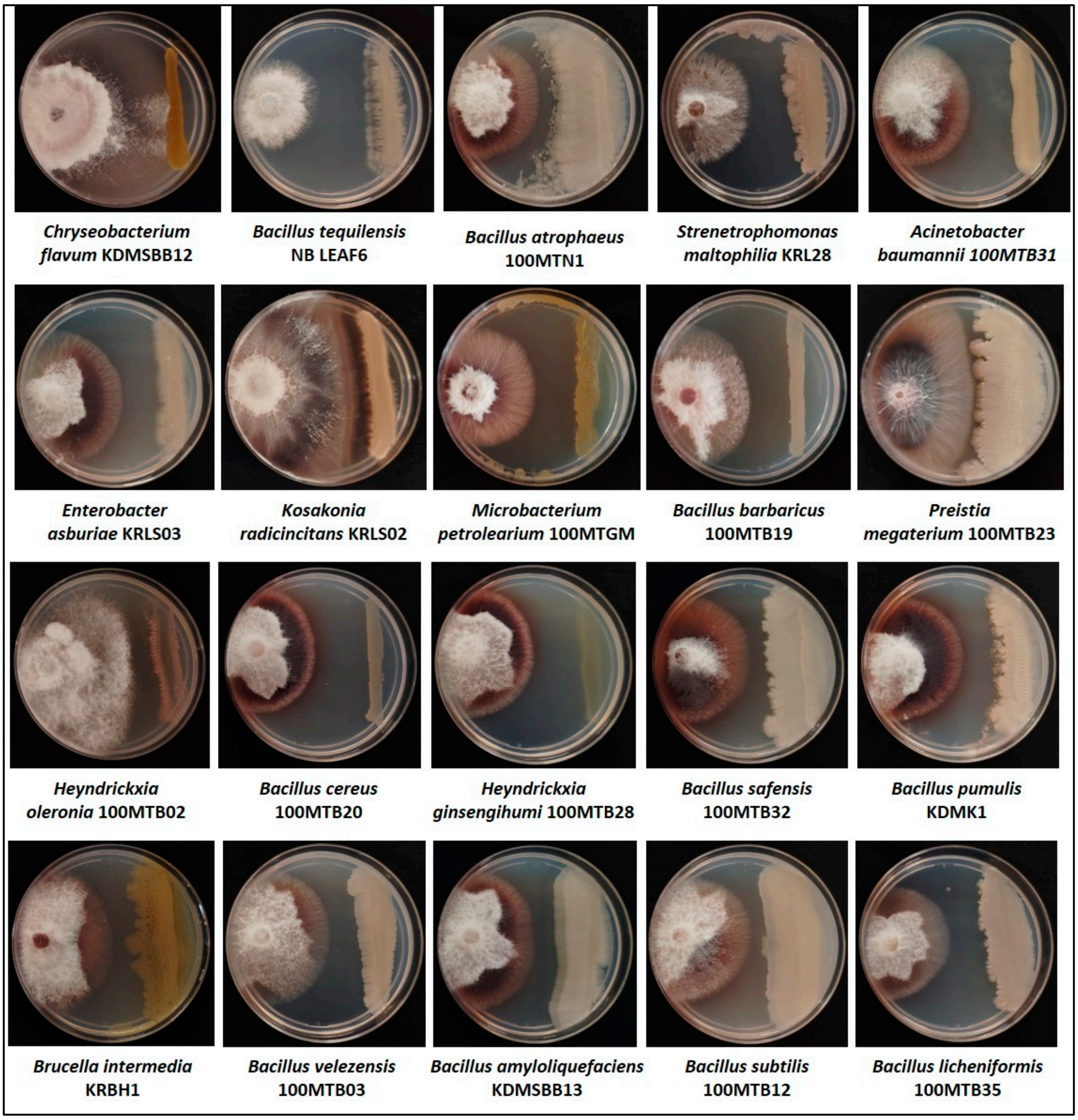

Figure S2. EzBioCloud 16S rRNA based identification of the bacterial strain 100MTN1. The image displays pairwise sequence alignment results, listing the closest published type strains ranked by sequence similarity percentage, along with completeness scores and taxonomic classifications.

List of hits from EzBioCloud 16S database

Select hits by database — All | Valid names only | Excel | FASTA | EzEditor2

Tasks	Hit taxon name	Hit strain name	Accession	Similarity	Variation ratio	Hit taxonomy	Completeness (%)
⇄ O	Bacillus mexicanus	FSQ1(T)	JAHAWP010000006	99.06	12/1276	Bacteria;Bacillota;Bacilli;Bacillales;Bacillaceae;Bacillus	88.2
⇄ O	Bacillus atrophaeus	JCM 9070(T)	AB021181	98.96	15/1441	Bacteria;Bacillota;Bacilli;Bacillales;Bacillaceae;Bacillus	100.0
⇄ O	Bacillus velezensis	CR-502(T)	AY603658	98.63	19/1386	Bacteria;Bacillota;Bacilli;Bacillales;Bacillaceae;Bacillus	95.4
⇄ O	Bacillus nakamurai	NRRL B-41091(T)	LSAZ01000028	98.54	21/1441	Bacteria;Bacillota;Bacilli;Bacillales;Bacillaceae;Bacillus	100.0
⇄ O	Bacillus spizizenii	NRRL B-23049(T)	CP002905	98.54	21/1441	Bacteria;Bacillota;Bacilli;Bacillales;Bacillaceae;Bacillus	100.0

Figure S3. Phylogenetic analysis of the 20 bacteria isolated from SCB and construction of phylogenetic tree generated using neighbour joining method with 1000 boot strap replication pertaining to the cut off value of 70%.

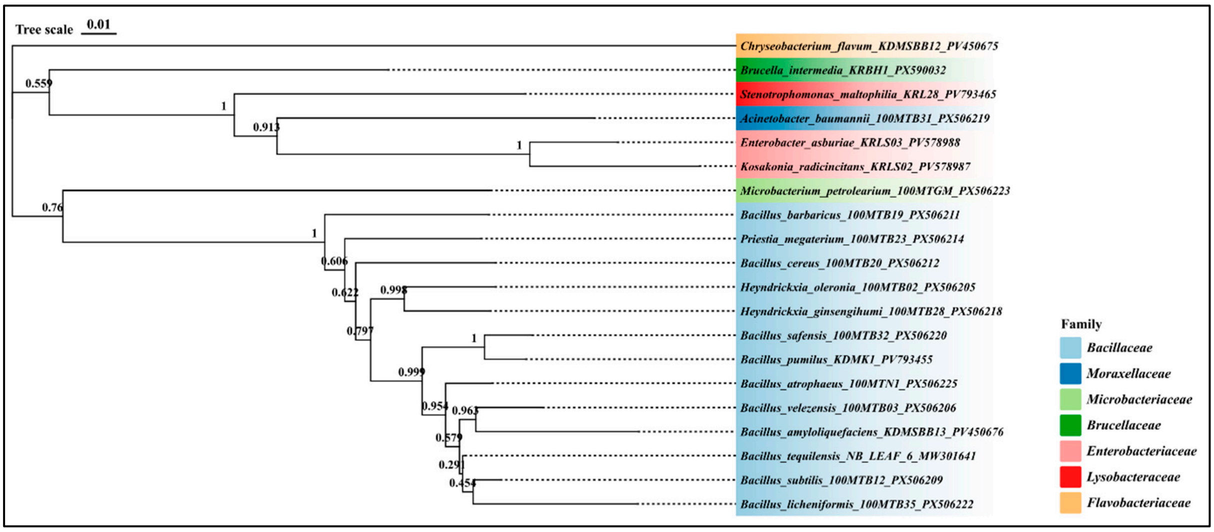

Table S1. An ANOVA table analysing the data of plant height of tomato recorded at 30 DPI. The table displays a standard statistical columns including sources of variation, degrees of freedom, and sum of squares, mean squares, F-values, and P-values, showing statistically significant differences across the evaluated treatments.

Source of variation	Degrees of freedom	Sum of squares	Mean sum of squares	F cal	F prob
Treatments	6	255.611	42.602	126.720	0.000
Error	14	4.707	0.336	-	-
Total	20	-	-	-	-

Coefficient of Variation = 1.854
Treatments found Significant at 1% and 5% level of significance
CD (0.01) = 1.409 CD (0.05) = 1.015

Table S2. An ANOVA table analysing the data of plant height of tomato recorded at 60 DPI. The table displays a standard statistical columns including sources of variation, degrees of freedom, and sum of squares, mean squares, F-values, and P-values, showing statistically significant differences across the evaluated treatments.

Source of variation	Degrees of freedom	Sum of squares	Mean sum of squares	F cal	F prob
Treatments	6	604.571	100.762	348.600	0.000
Error	14	4.047	0.289	-	-
Total	20	-	-	-	-

Coefficient of Variation = 0.883
Treatments found Significant at 1% and 5% level of significance
CD (0.01) = 1.307 CD (0.05) = 0.942

Table S3. An ANOVA table analysing the data of plant height of tomato recorded at 90 DPI. The table displays a standard statistical columns including sources of variation, degrees of freedom, and sum of squares, mean squares, F-values, and P-values, showing statistically significant differences across the evaluated treatments.

Source of variation	Degrees of freedom	Sum of squares	Mean sum of squares	F cal	F prob
Treatments	6	2233.543	372.257	1386.064	0.000
Error	14	3.760	0.269	-	-
Total	20	-	-	-	-

Coefficient of Variation = 0.609
Treatments found Significant at 1% and 5% level of significance
CD (0.01) = 1.260 CD (0.05) = 0.908

Table S4. An ANOVA table analysing the data of fusarium wilt incidence recorded at 90 DPI. The table displays a standard statistical columns including sources of variation, degrees of freedom, and sum of squares, mean squares, F-values, and P-values, showing statistically significant differences across the evaluated treatments.

Source of variation	Degrees of freedom	Sum of squares	Mean sum of squares	F cal	F prob
Treatments	6	12628.310	2104.718	720.911	0.000
Error	14	40.873	2.920	-	-
Total	20	-	-	-	-

Coefficient of Variation = 5.269
Treatments found Significant at 1% and 5% level of significance
CD (0.01) = 4.153 CD (0.05) = 2.993

Figure S4. GC-MS Chromatogram depicting the profile of VOCs/NVOCs extracted from the zone of inhibition of dual confrontation assay between *B. atrophaeus* 100MTN1 and *Fol* FOLViF. The x-axis represents retention time in minutes and the y-axis represents peak intensity, with multiple distinct peaks labelled to indicate individual volatile and non-volatile organic compounds.

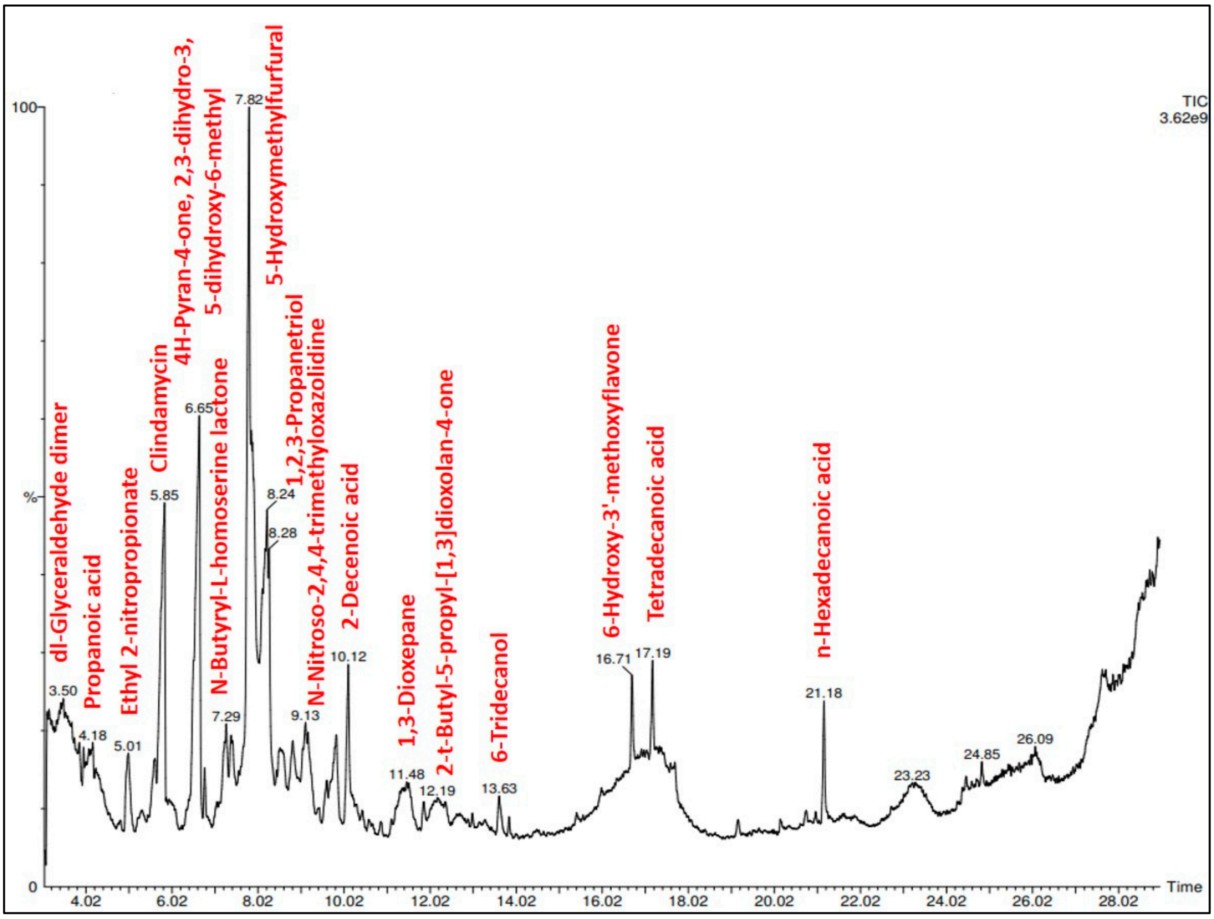

Figure S5. Principal Component Analysis of normalized transcriptome profiles for *Fol* FOLViF mycelium. The plot shows distinct clustering that separates the control *Fol* FOLViF from the *Fol* FOLViF antagonized by *B. atrophaeus* 100MTN1, illustrating a clear shift in the transcriptome profile due to bacterial antagonism.

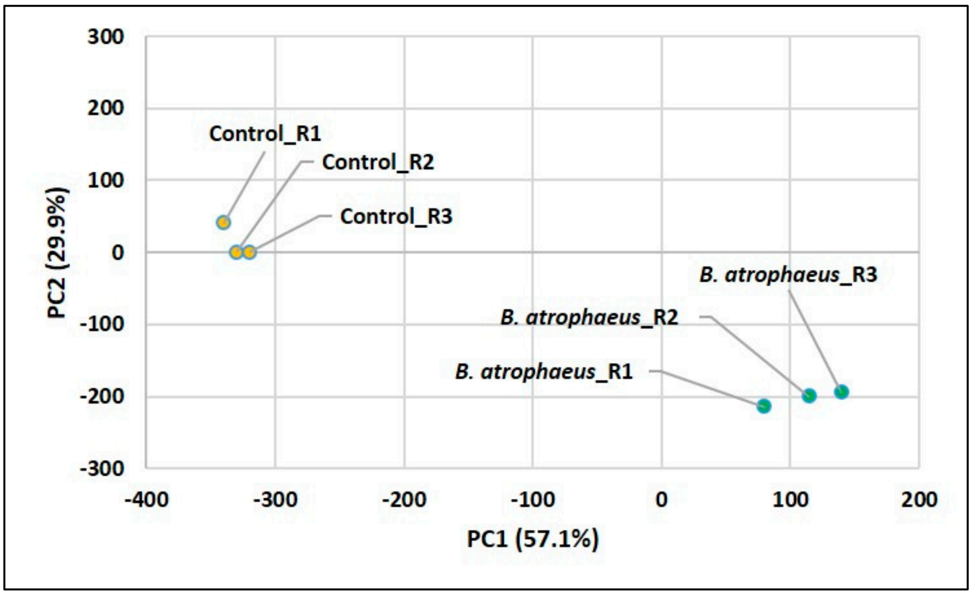

Figure S6. Volcano plot depicting differentially expressed genes (DEGs) in the mycelium of *Fol* FOLViF antagonized by *B. atrophaeus* 100MTN1 compared to untreated *Fol* FOLViF mycelium. The plot displays a subset of 17 genes selected based on the adjust P-value of 2, highlighting distinct clusters of upregulated and downregulated genes resulting from the bacterial antagonism.

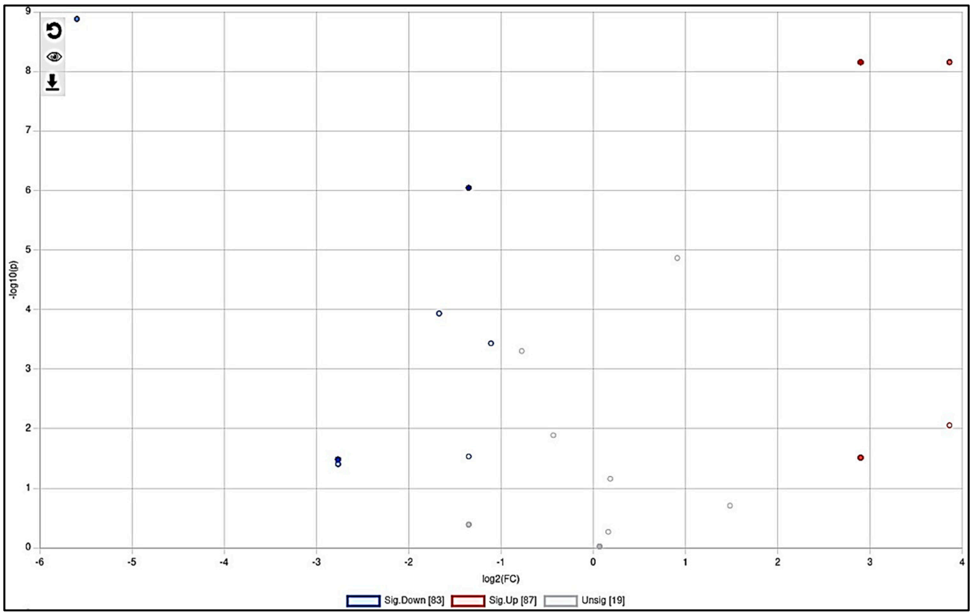

Figure S7. Downregulation of *RPL11*, *RPL32*, *RPL35* and *RPL3* (log_2FC: -1.35) genes, and upregulation of *RPS26*, *RPL9*, *RPL38* (log_2FC: 2.90) genes involved in large and small subunit of the ribosome in the mycelium of *Fol* FOLViF antagonized *by B. atrophaeus* 100MTN1.

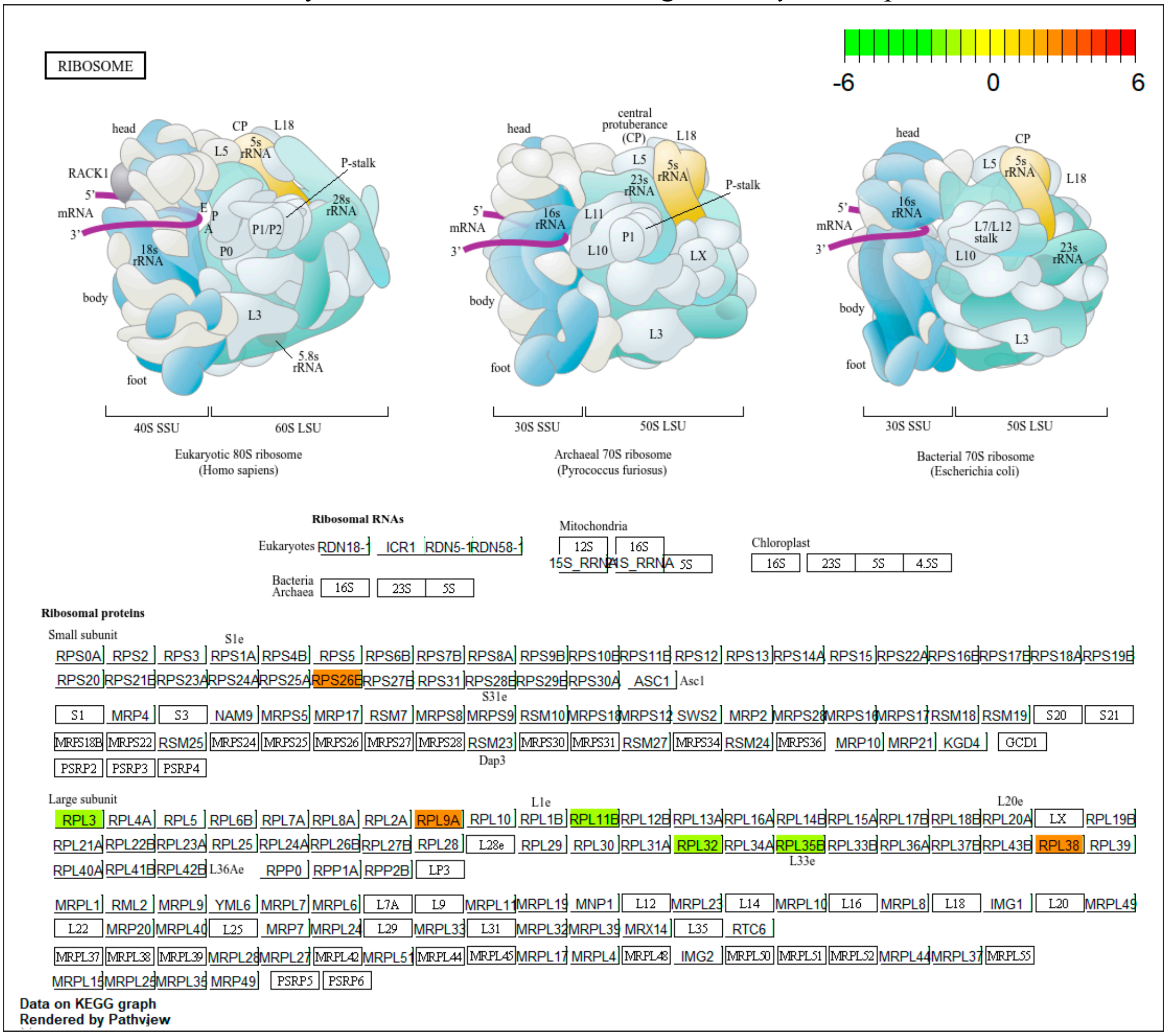

Figure S8. A biochemical pathway map illustrating the biosynthesis of amino acid pathway in the mycelium of *Fol* FOLViF during antagonism by *B. atrophaeus* 100MTN1. The visual highlights the downregulation of genes *ENO1* and *GLN1* with a $\log_2$FC of -1.35, contrasted against the upregulation of genes *LYS2*, *CAR1*, and *HIS2* with a $\log_2$FC of 2.90.

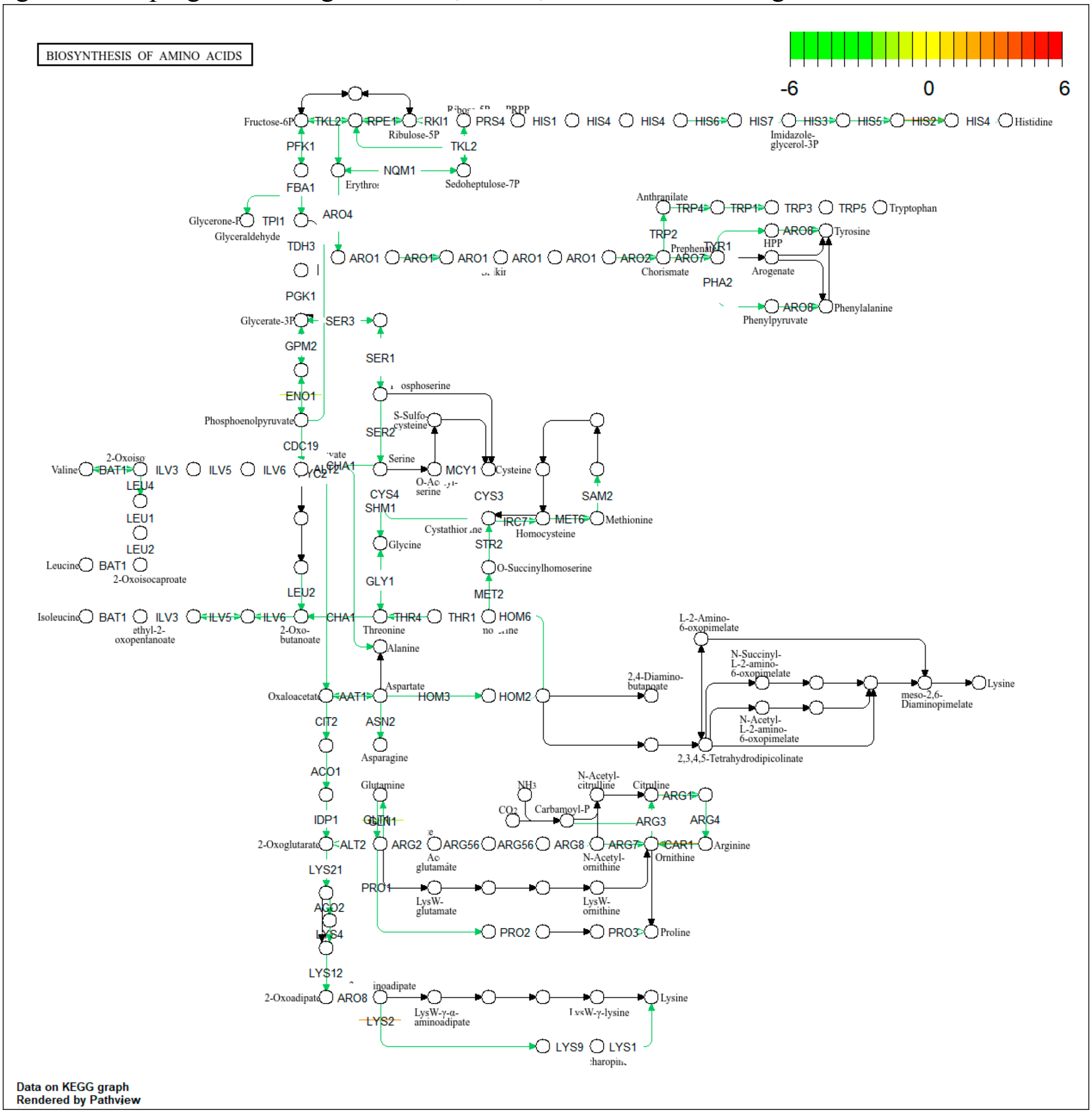

Figure S9. Diagram showing the downregulation of oxidative phosphorylation pathway genes *VMA10* and *TFP1* (log_2FC: -1.35) in *Fol* FOLViF mycelium antagonized by *B. atrophaeus* 100MTN1 compared to untreated *Fol* FOLViF mycelium alone.

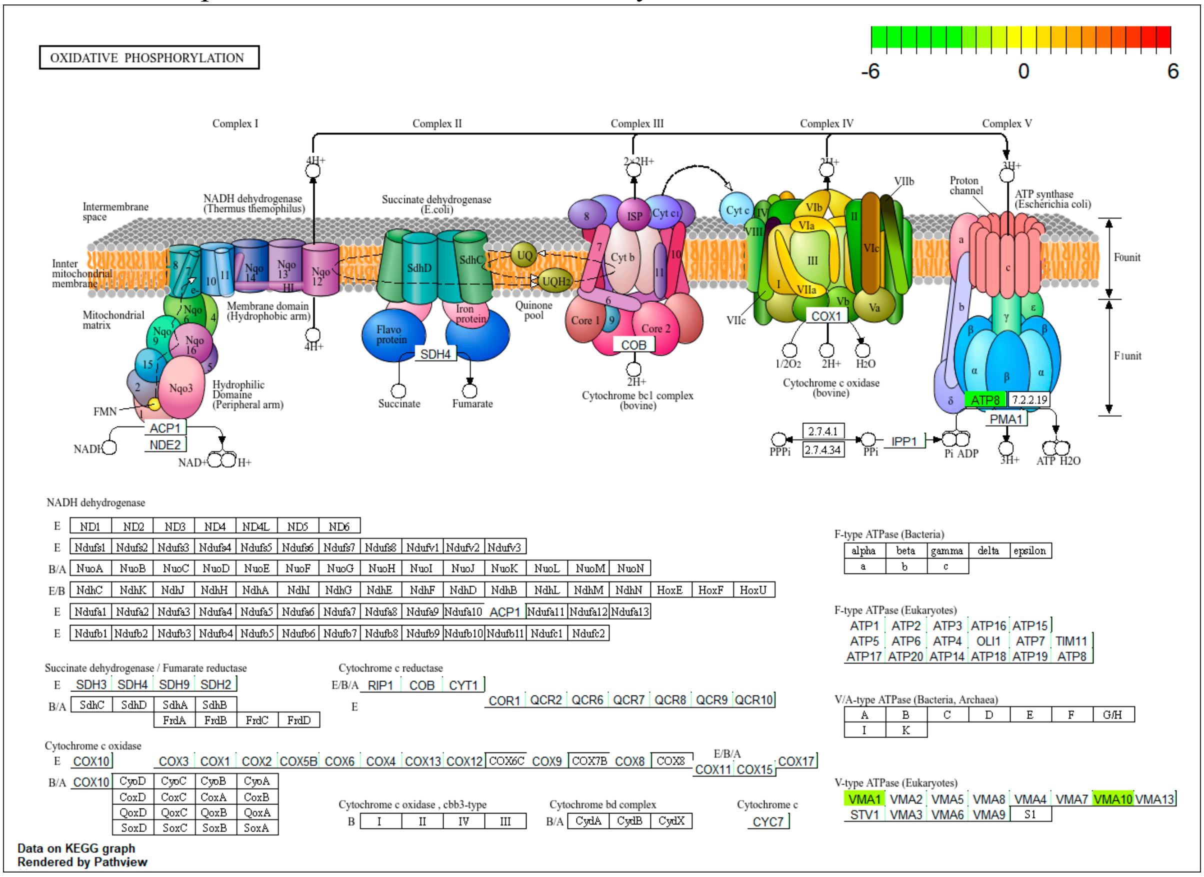

Figure S10. A cellular pathway diagram illustrating endocytosis activity in the mycelium of *Fol* FOLViF antagonised by *B. atrophaeus* 100MTN1 depicting the specific transcriptional changes of key regulatory genes, highlighting the downregulation of *ARF1* with a $\log_2$FC of -1.35 alongside the concurrent upregulation of the *END3* gene with a $\log_2$FC of 2.90.

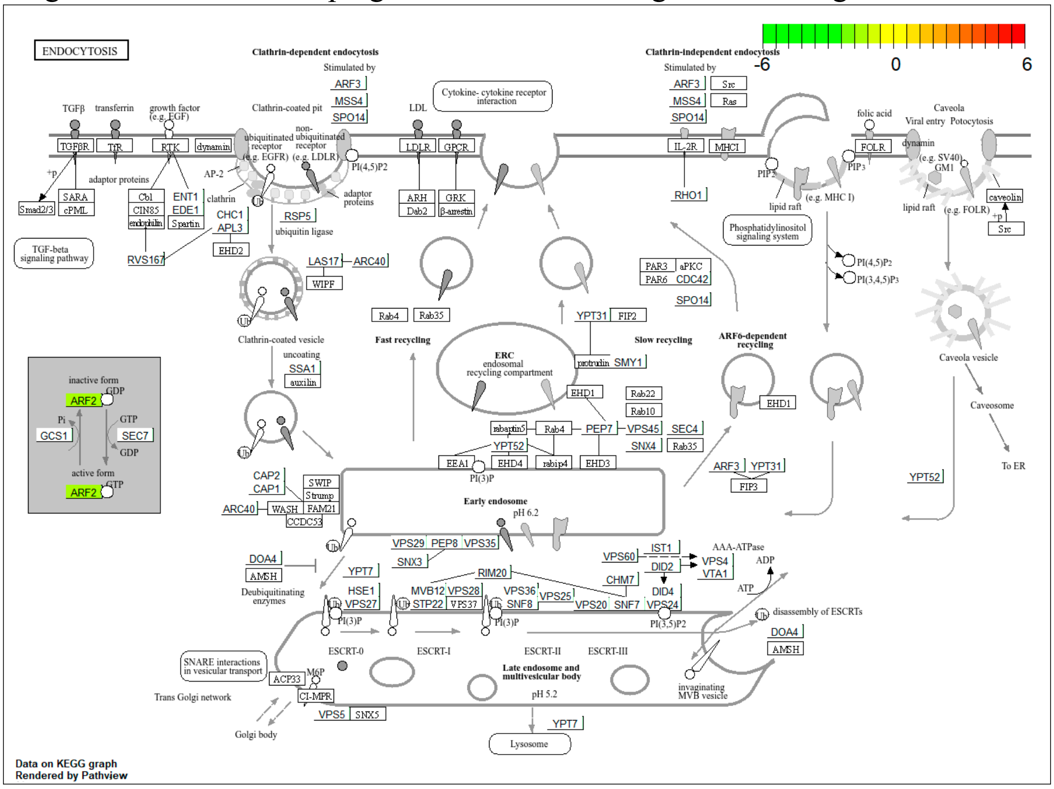

Figure S11. Illustration of the riboflavin metabolism pathway in *Fol* FOLViF mycelium antagonized by *B. atrophaeus* 100MTN1, marking the repression of *RIB4* and *FAD1* genes with a $\log_2$FC value of -1.35.

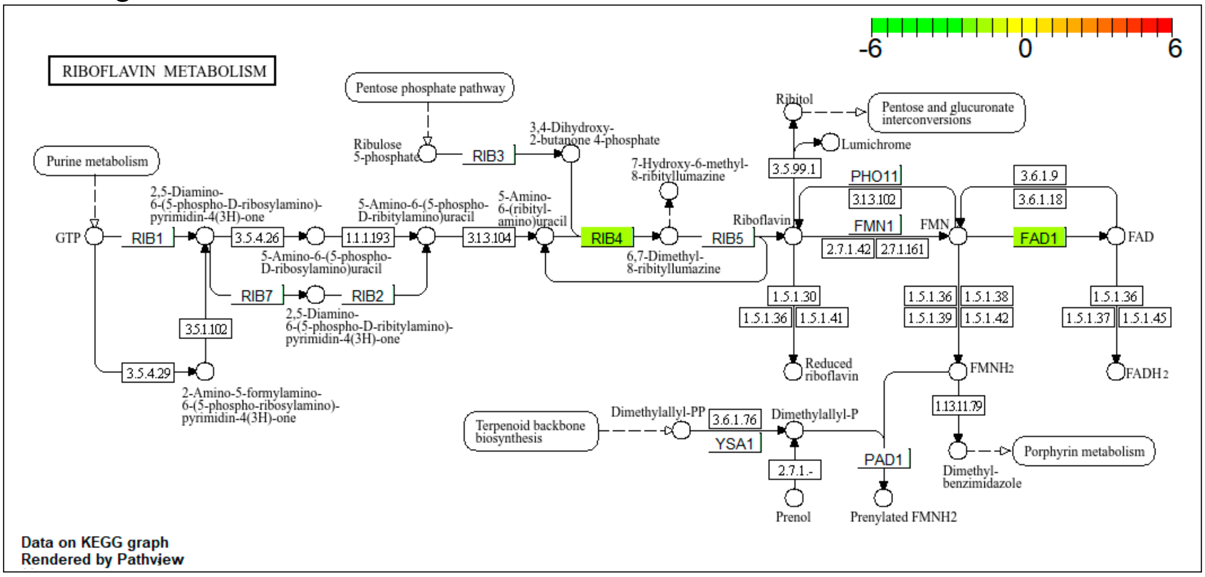

Figure S12. Illustration of cellular mechanism explaining the downregulation of specific target genes, including *VMA10* and *TFP1* with a $\log_2$FC of -1.35, actively represses phagosome pathway activity within the mycelium of *Fol* FOLViF antagonised by *B. atrophaeus* 100MTN1.

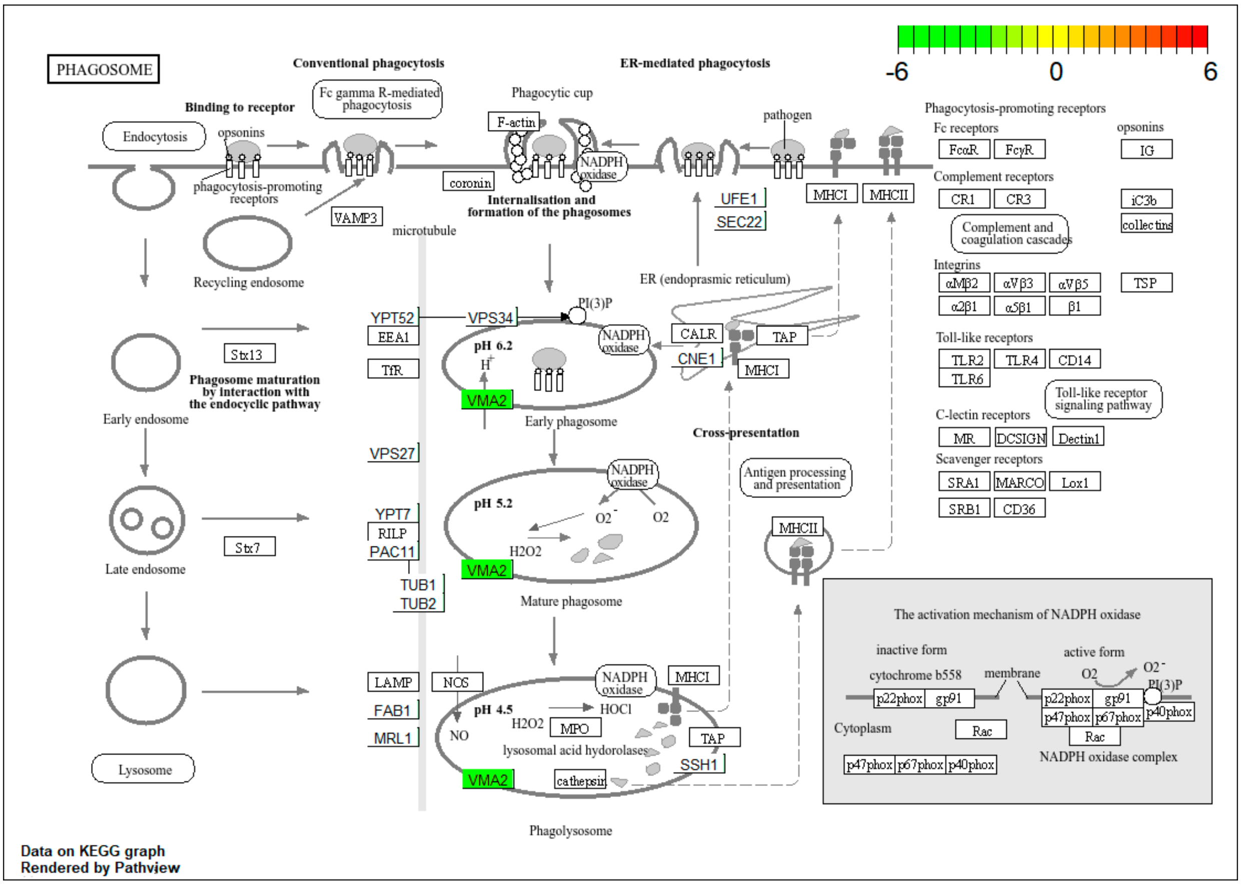

Figure S13. Metabolic pathway map illustrating the carbon metabolism pathway in the mycelium of *Fol* FOLViF during antagonism by *B. atrophaeus* 100MTN1. The diagram visually highlights the transcriptional upregulation of the *ENO1* gene, explicitly displaying its calculated $\log_2$FC value of 2.90.

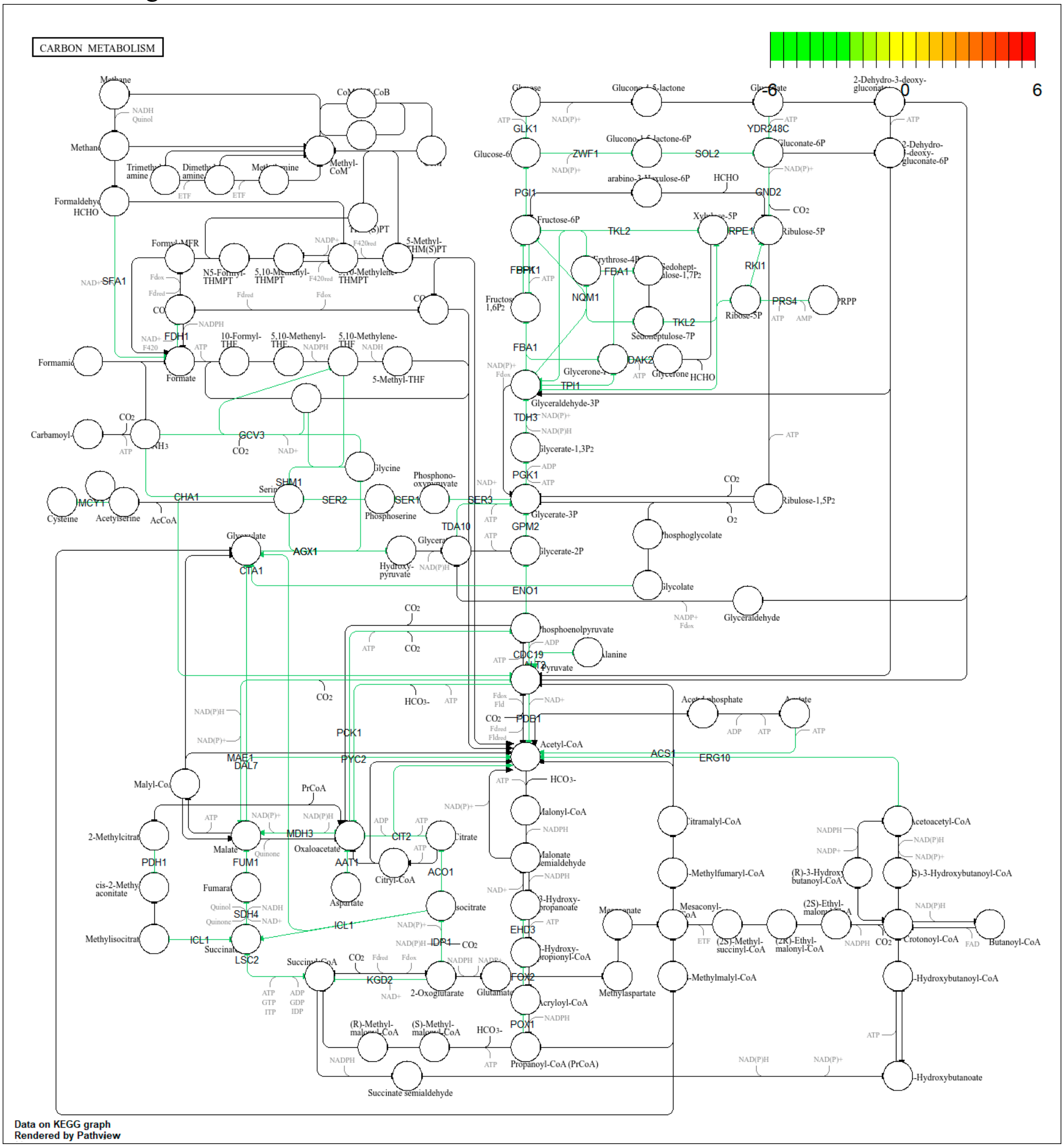

Figure S14. Illustration of the pyruvate metabolism pathway in *Fol* FOLViF mycelium antagonized by *B. atrophaeus* 100MTN1, depicting the downregulation of the *DLD1* gene with a $\log_2$FC value of -1.35.

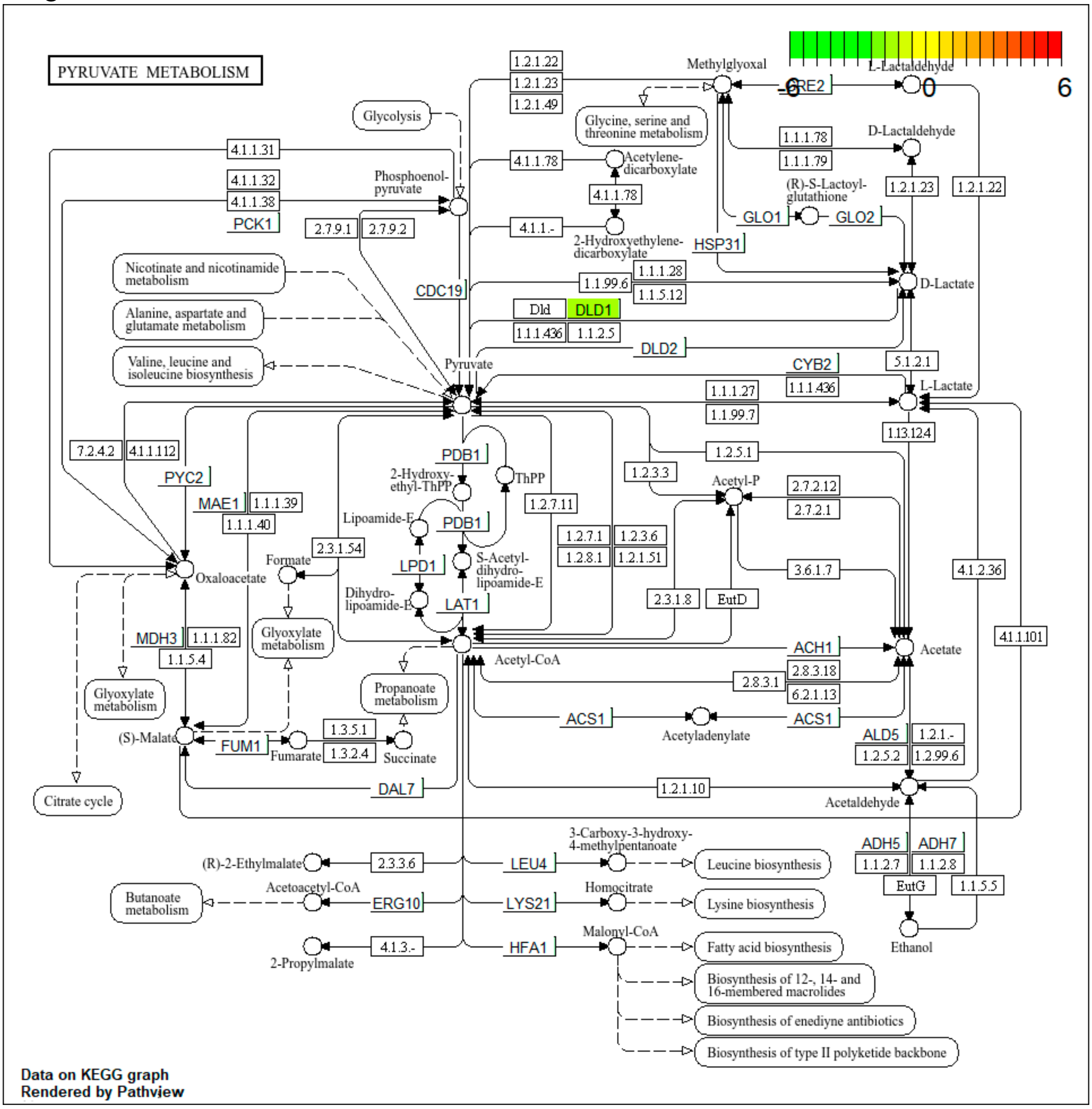

Figure S15. Illustration of the Inositol phosphate metabolism pathway in *Fol* FOLViF mycelium antagonized by *B. atrophaeus* 100MTN1, showing the repression of the *PLC1* gene with a $\log_2$FC value of -1.35.

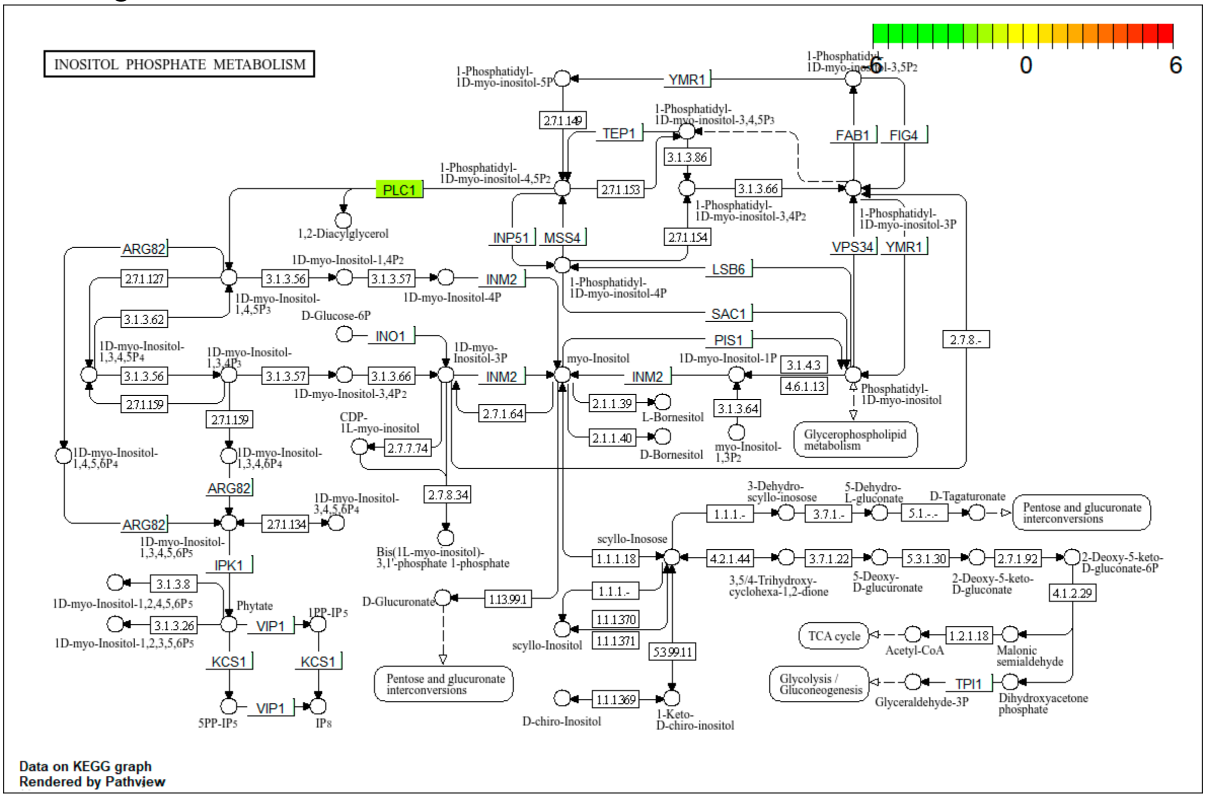

Figure S16. Metabolic pathway map illustrating the Pentose phosphate pathway in the mycelium of *Fol* FOLViF during antagonism by *B. atrophaeus* 100MTN1, highlighting the transcriptional downregulation of the *RBK1* gene, explicitly displaying its calculated log2FC value of -1.35.

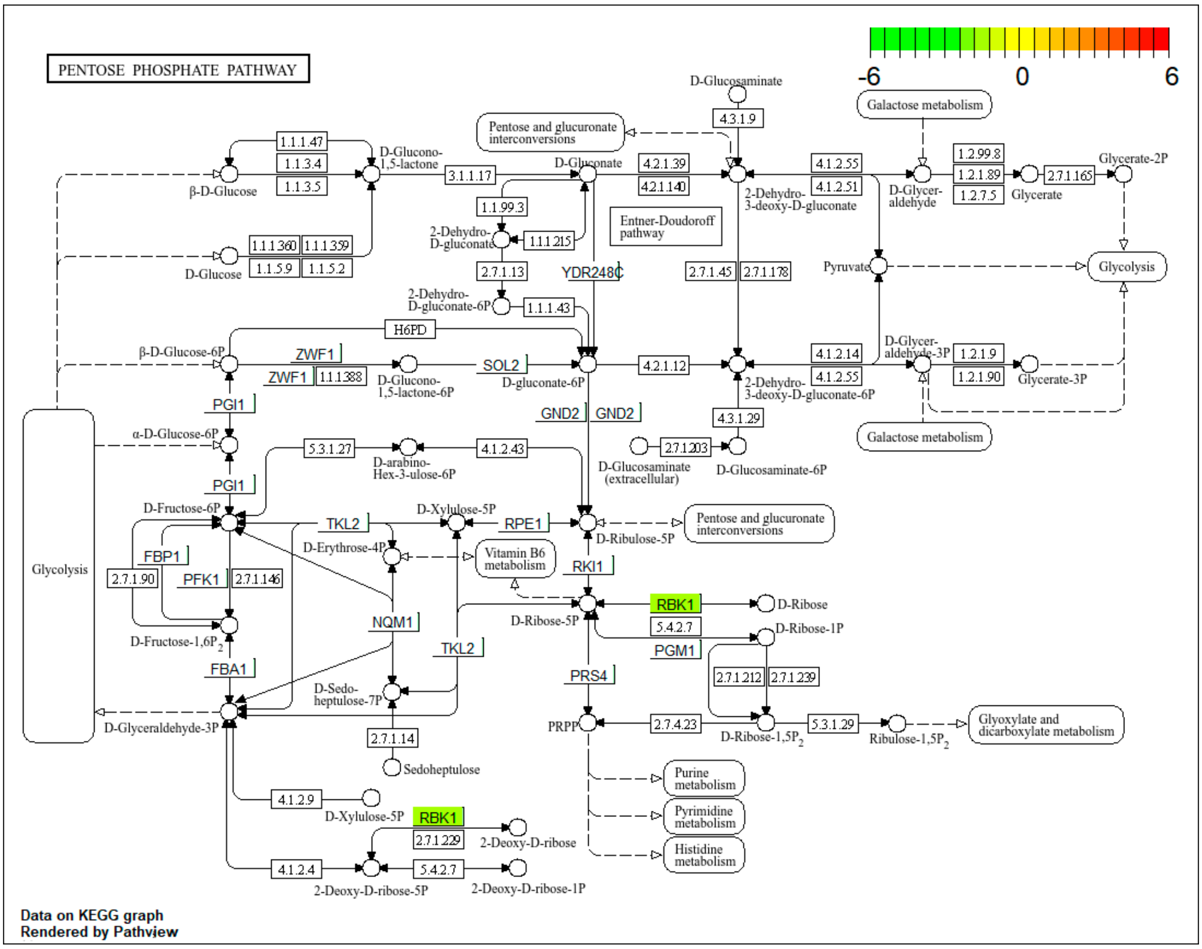

Figure S17. Pathway diagram showing the downregulation of the *CDC34* gene (log_2FC: -1.35) within the ubiquitin-mediated proteolysis system of *Fol* FOLViF mycelium antagonised by *B. atrophaeus* 100MTN1.

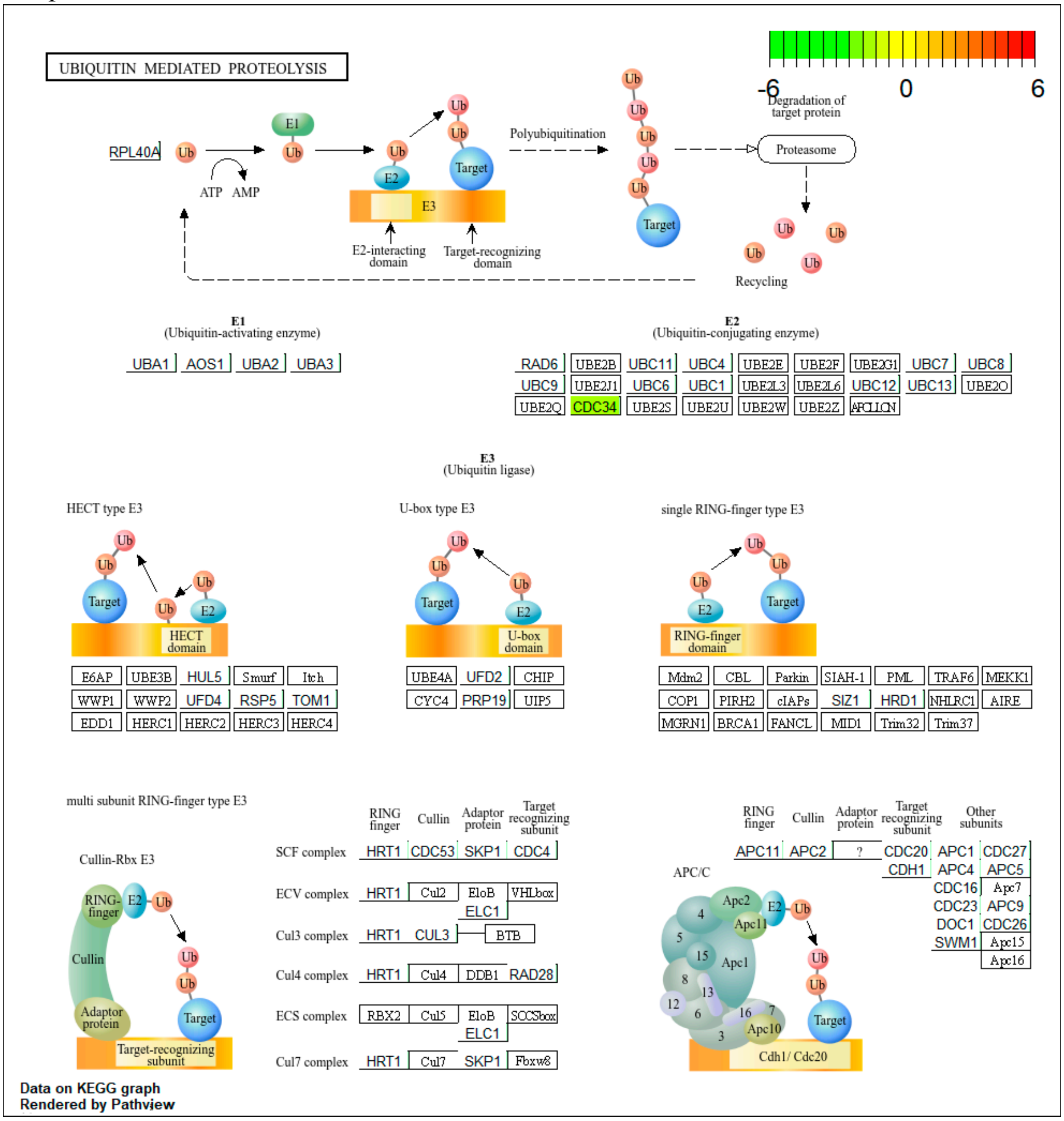

Figure S18. Illustration of the terpenoid backbone biosynthesis pathway in *Fol* FOLViF mycelium antagonized by *B. atrophaeus* 100MTN1, showing the downregulation of the *RAM1* gene with a $\log_2$FC value of -1.35.

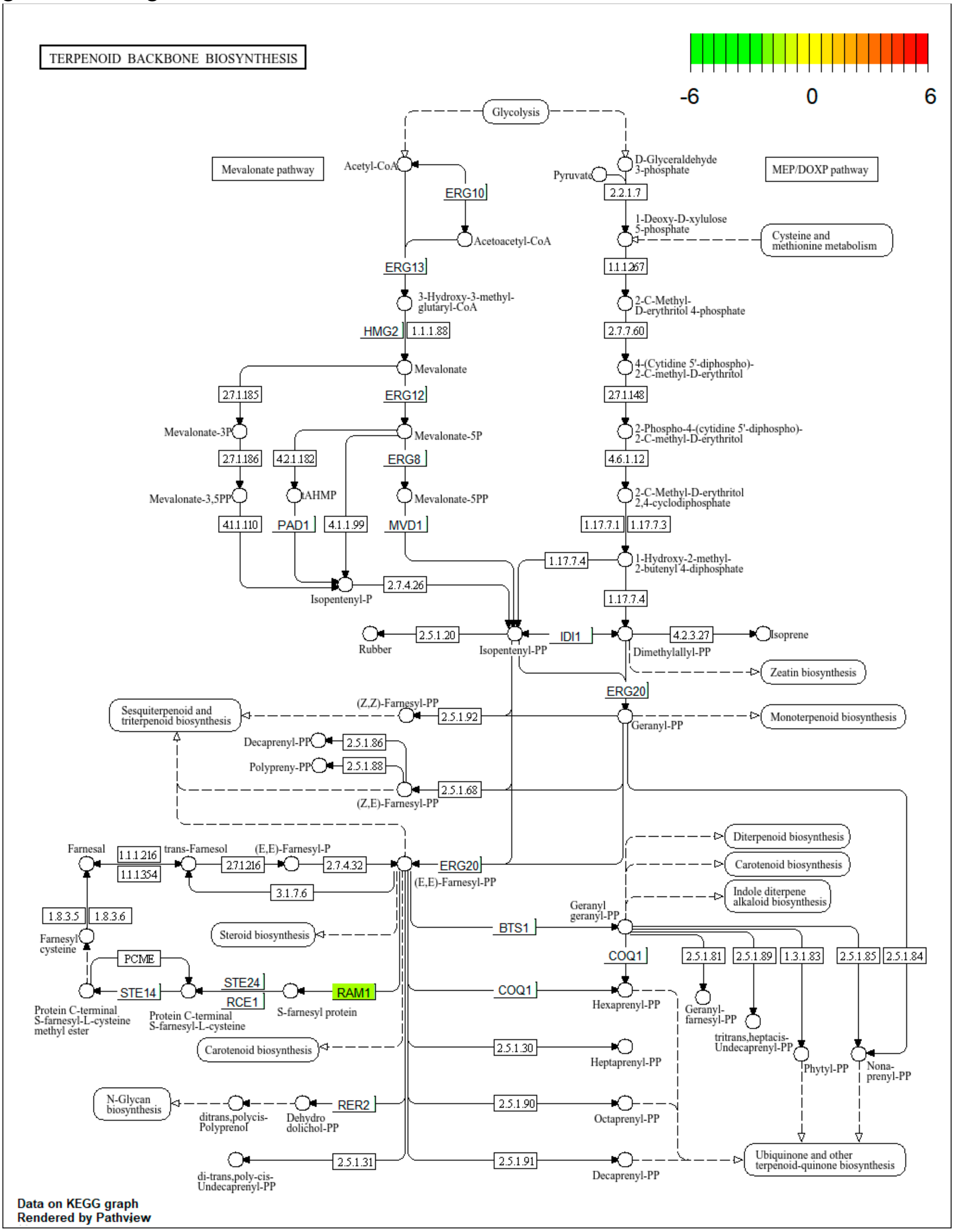

Figure S19. Pathway diagram showing the downregulation of the *ENO1* gene (log_2FC: -1.35) within the RNA degradation machinery of *Fol* FOLViF mycelium under *B. atrophaeus* 100MTN1 antagonism.

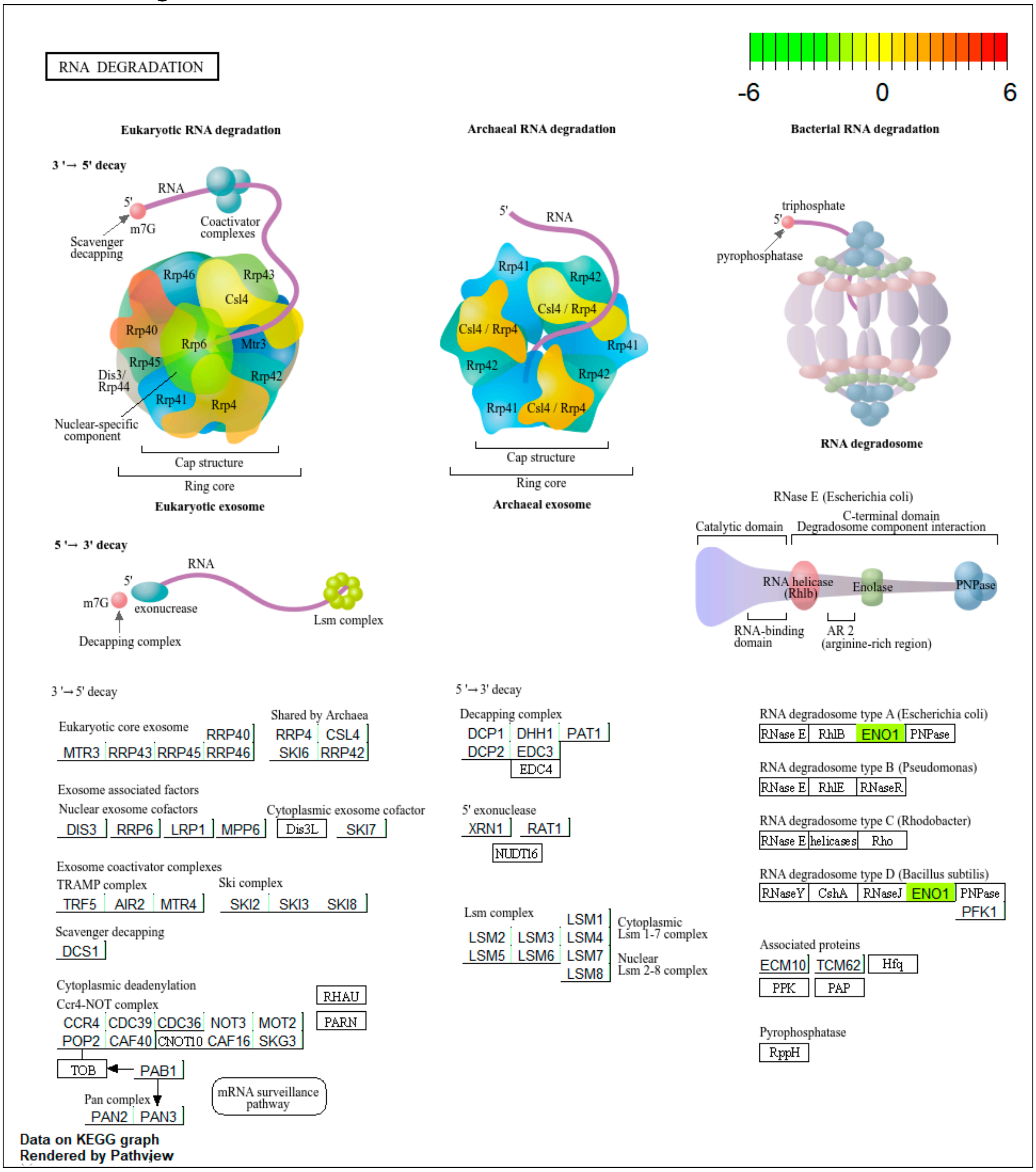

Figure S20. DNA repair pathway diagram illustrating the nucleotide excision repair mechanism in the mycelium of *Fol* FOLViF during antagonism by *B. atrophaeus* 100MTN1, highlighting the transcriptional downregulation of the *RFC2* gene, with $\log_2$FC value of -1.35.

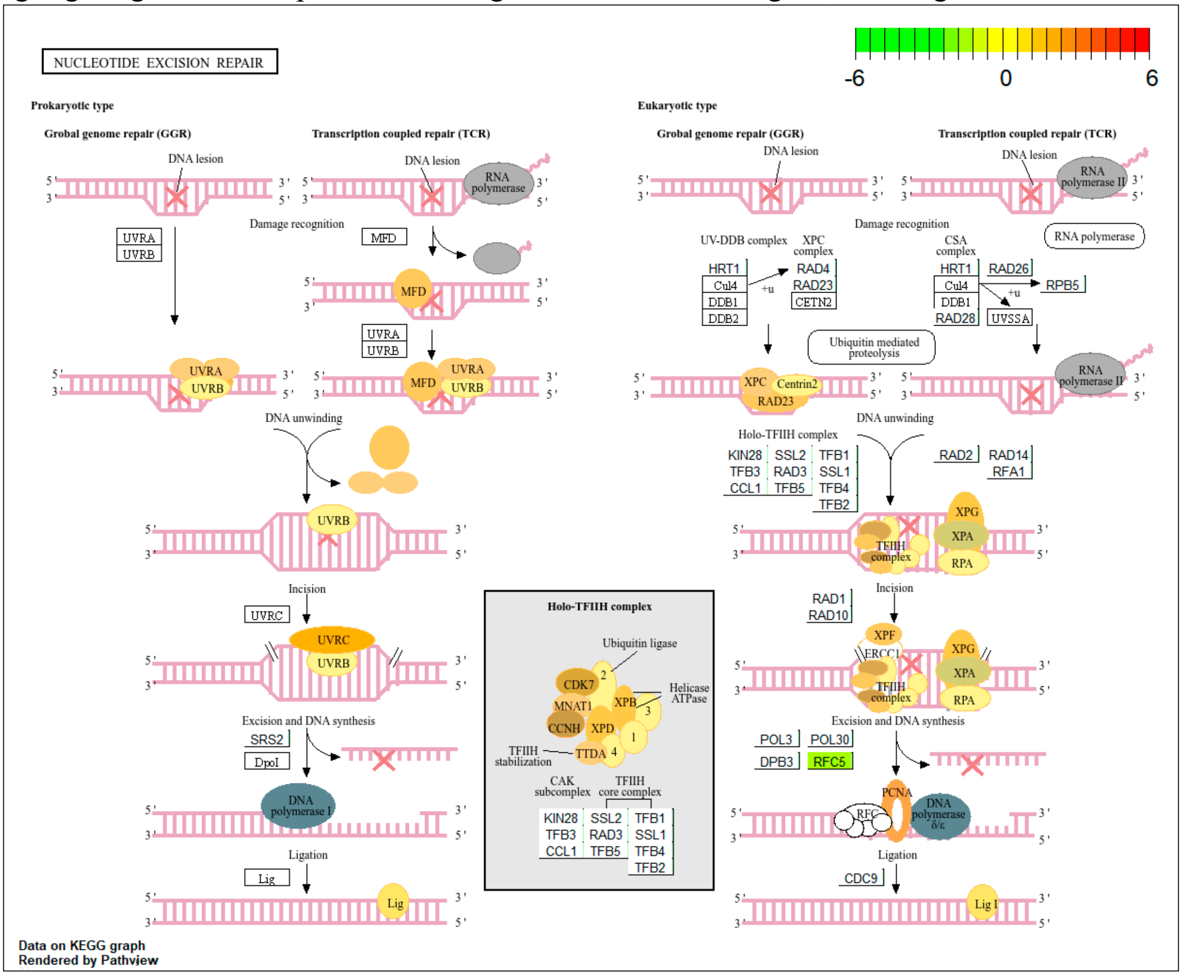

Figure S21. Molecular pathway diagram of the DNA replication machinery in the mycelium of *Fol* FOLViF antagonised by *B. atrophaeus* 100MTN1, depicting the targeted transcriptional downregulation of the replication factor C subunit gene, *RFC2*, with an explicit $\log_2FC$ value of -1.35.

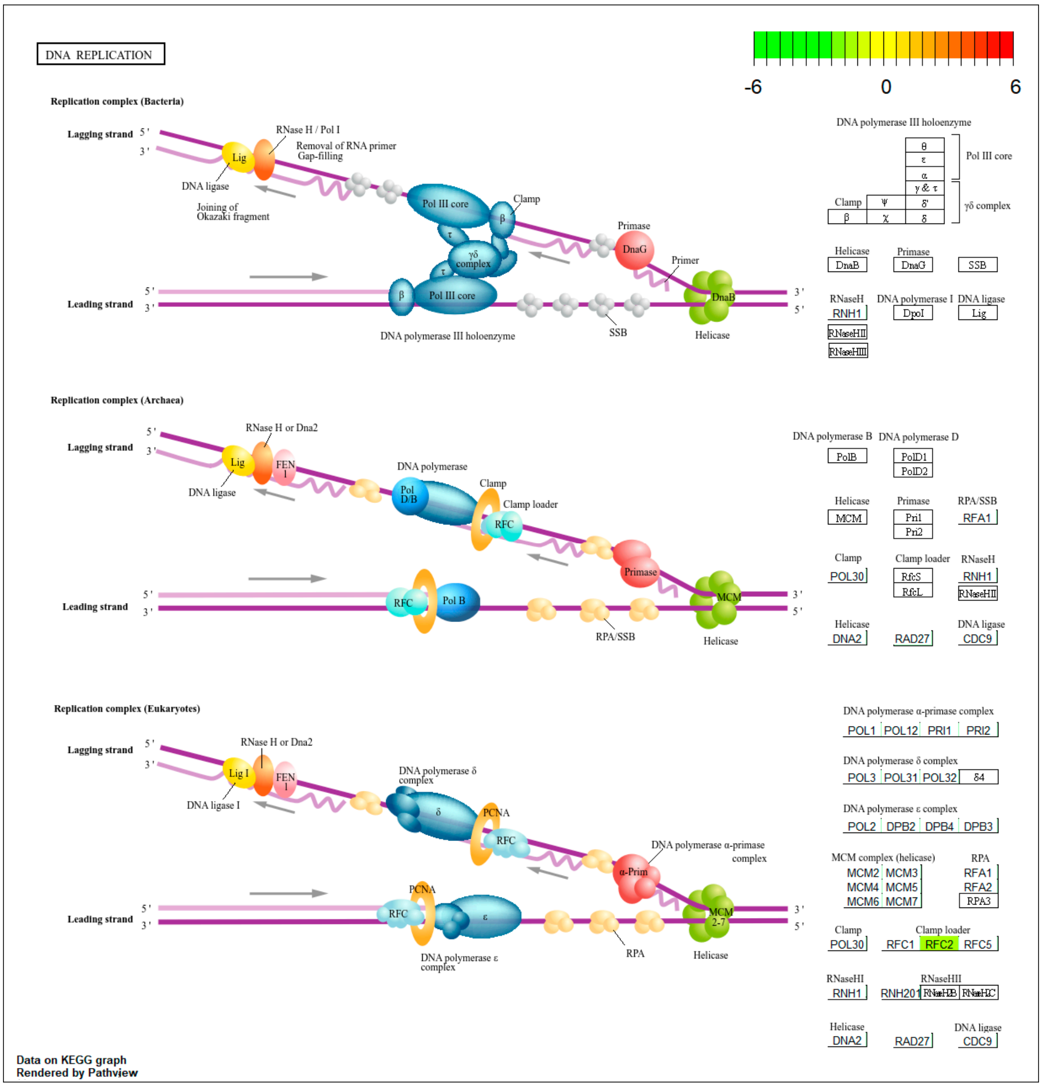

Figure S22. A pathway scheme showing cellular components of mismatch repair in *Fol* FOLViF mycelium antagonised *B. atrophaeus* 100MTN1. The *RFC2* gene node is highlighted with color-coding indicating a downregulated log2FC value of -1.35.

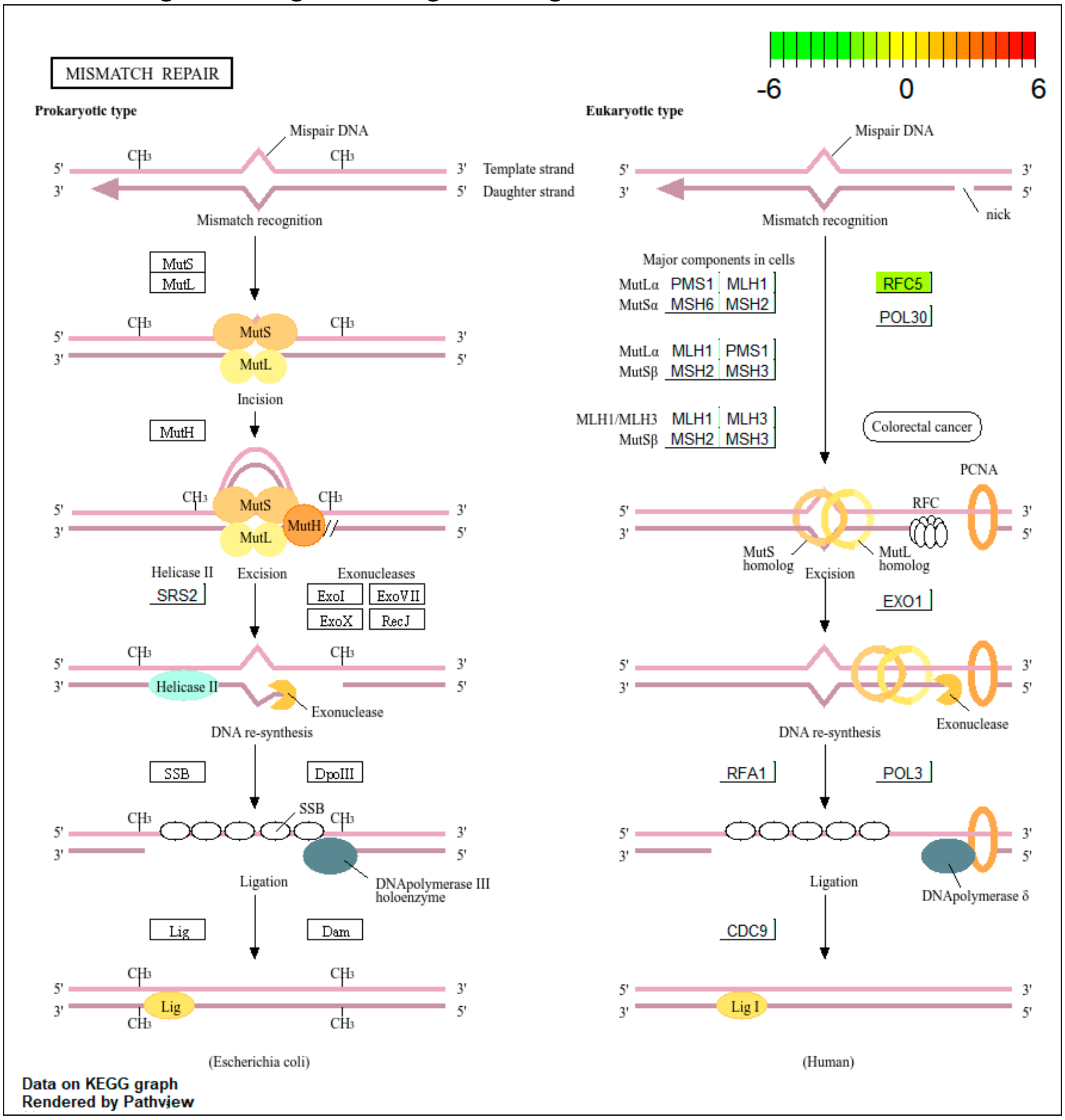

Figure S23. Gene expression profile within the central carbon metabolism (glycolysis/gluconeogenesis) of *Fol* FOLViF mycelium antagonized by *B. atrophaeus* 100MTN1, mapping the downregulation of *ENO1* at log_2FC -1.35.

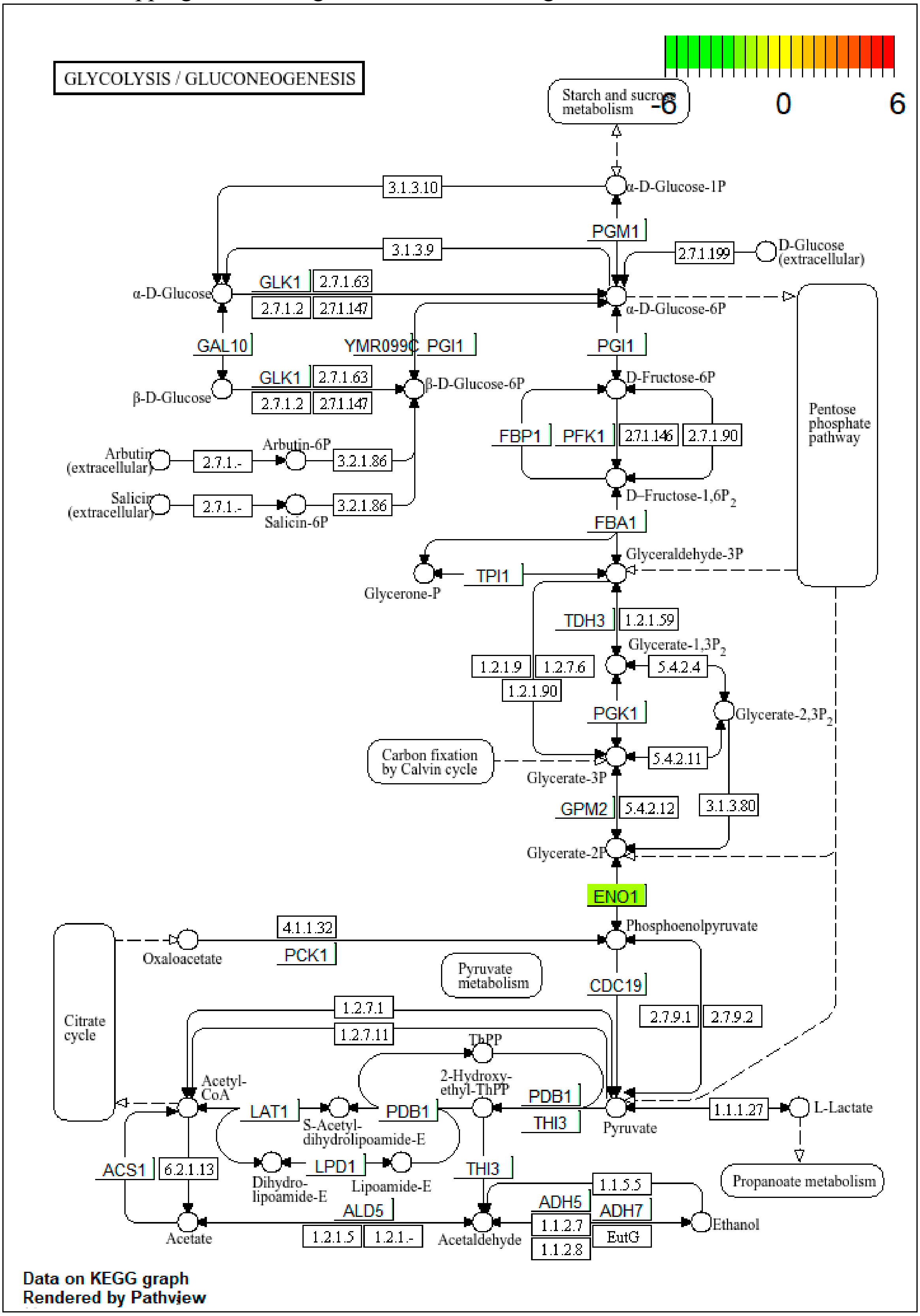

Figure S24. A transcriptomic data diagram plotting the nitrogen metabolism pathway of *Fol* FOLViF mycelium antagonised *B. atrophaeus* 100MTN1, emphasizing a negative log2FC value of -1.35 for the *GLN1* gene, indicating significant downregulation.

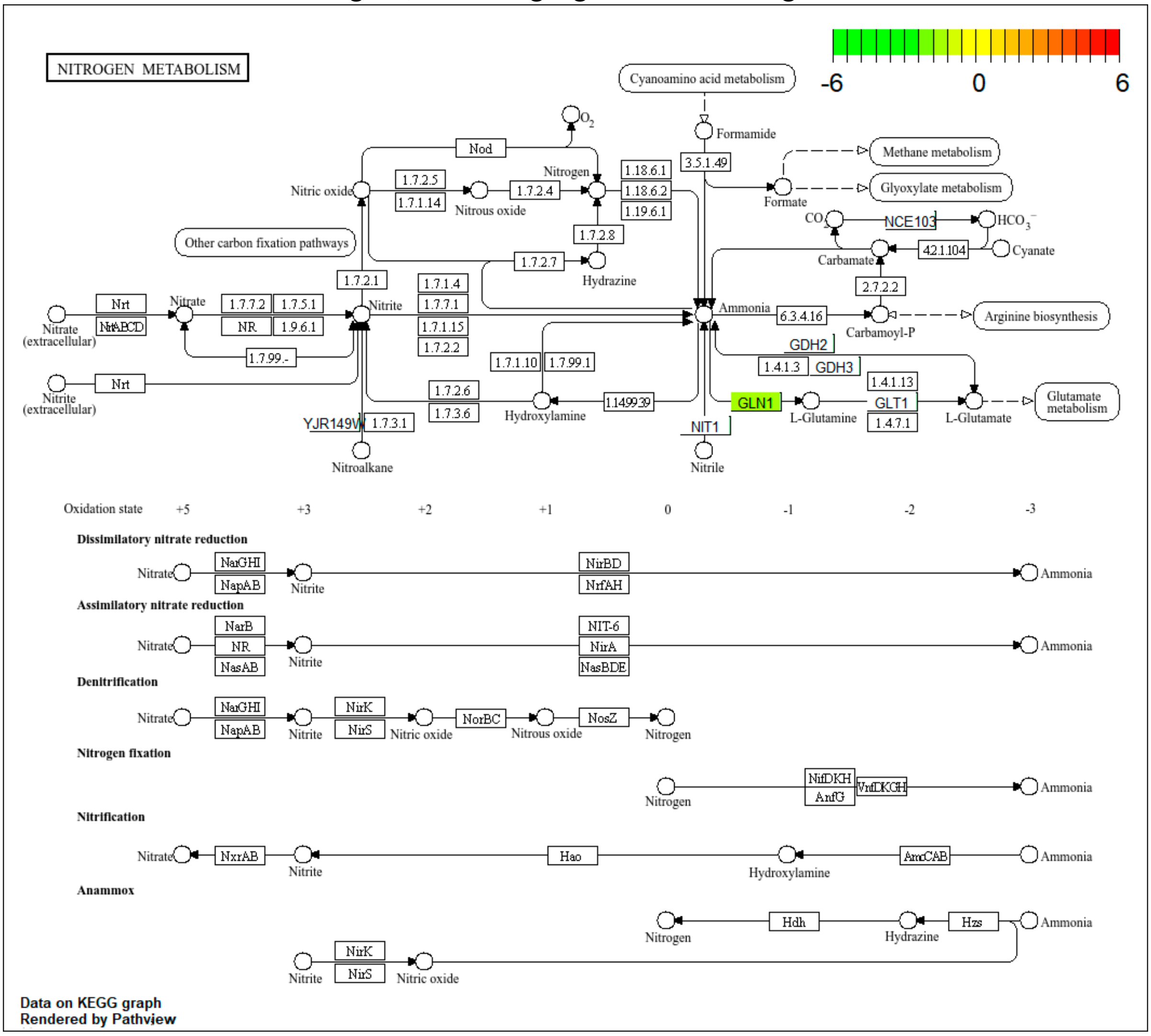

Figure S25. Gene expression profile within the peroxisome pathway of *Fol* FOLViF mycelium antagonized by *B. atrophaeus* 100MTN1, mapping the upregulation of *PEX19* at log_2FC 2.90.

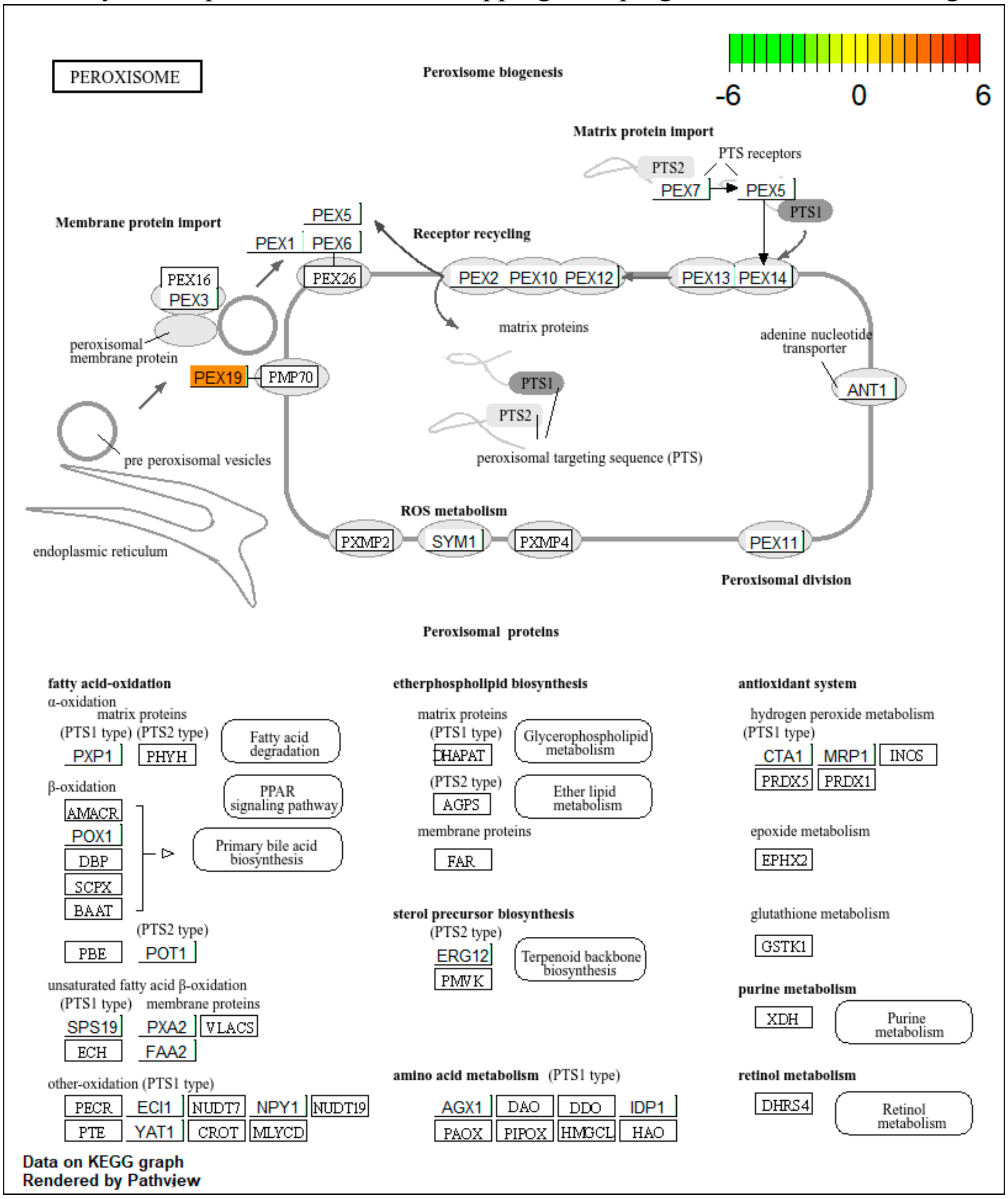

Table S5. The amino acid residues of the target proteins obtained in different regions of the Ramachandran plot and the coordinates of their binding sites.

Target protein	Residues in core region (%)	Grid size of the binding sites in protein model
ACL1	98.16	X: 1.7816, Y: -1.7287, Z: -3.4028
PLC1	88.75	X: -6.6569, Y: -1.7905, Z: 23.461
PTR2	95.78	X: -7.8423, Y: -6.4839, Z: 7.6809
EIF3S9	91.17	X: 0.9086, Y: -5.5196, Z: -22.7827
RAM1	97.78	X: -5.5454, Y: 7.4105, Z: -13.5400
RBK1	98.77	X: 10.1733, Y: -0.7202, Z: -0.4418
RPL3	98.45	X: -9.9156, Y: 0.0582, Z: -21.6286
RPL11	97.36	X: -5.7504, Y: -4.8464, Z: -2.5915
RRP5	86.21	X: 4.3144, Y: -4.7569, Z: 7.0162
SRD5A3	97.79	X: -4.6529, Y: 1.2385, Z: 9.9093
TFP1	89.94	X: 8.8451, Y: 2.1878, Z: 5.1129
UBA52	94.07	X: 22.5989, Y: -0.2741, Z: -34.7517
Histone deacetylase (HDA)	95.37	X: 5.7806, Y: -8.6280, Z: 16.6492
RBM27	84.61	X: -22.1962, Y: 4.9262, Z: -22.1209
ARF1	98.31	X: -3.8943, Y: 0.7314, Z: 0.3956
CDC34	96.64	X: 10.7945, Y: 2.7640, Z: -1.2221
SI:DKEY-3708.1	85.63	X: -1.9372, Y: -2.4601, Z: -2.0343
AN11G11290	94.83	X: 0.8358, Y: 31.3995, Z: 9.7576
RFC3	95.38	X: 11.1439, Y: -6.3035, Z: -9.6715
DLD1	95.61	X: 3.1416, Y: -6.5324, Z: -1.8641
GLN1	96.48	X: 3.4013, Y: 2.8662, Z: -8.1125
DDX54	91.62	X: -1.1345, Y: 0.1990, Z: -7.0999
HTA1	91.48	X: 1.1481, Y: 2.1162, Z: -1.3957

Figure S26. Ramachandran plot displaying the polypeptide backbone conformation (phi and psi torsion angles) for the protein targets associated with downregulated genes of *Fol* FOLViF antagonized by *B. atrophaeus* 100MTN1. The scatter plot maps the amino acid residues across sterically allowed, additionally allowed, and disallowed regions to evaluate structural validity.

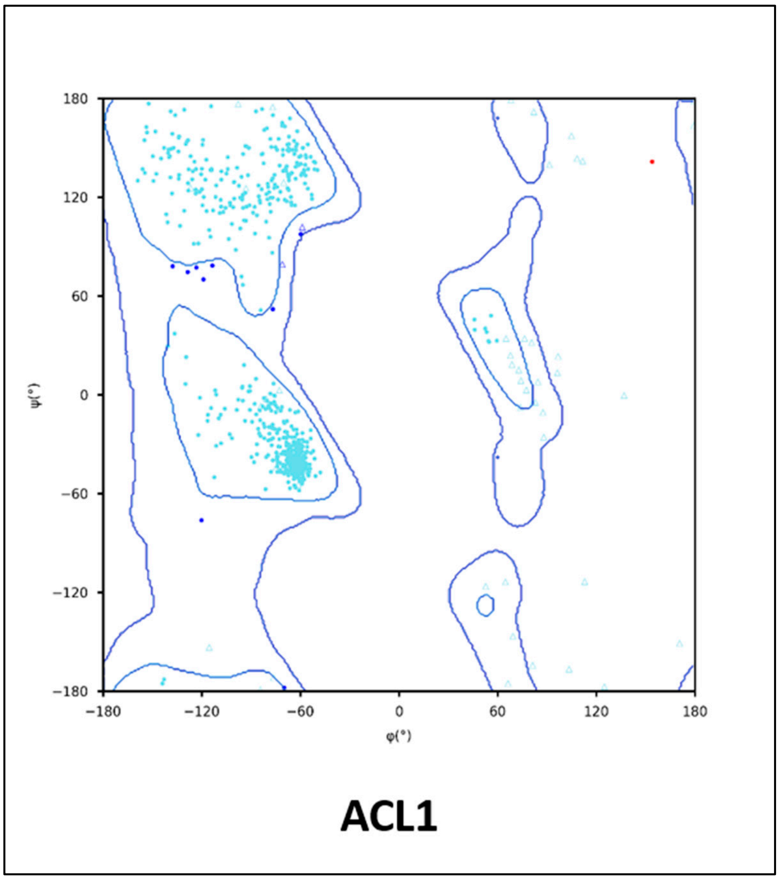

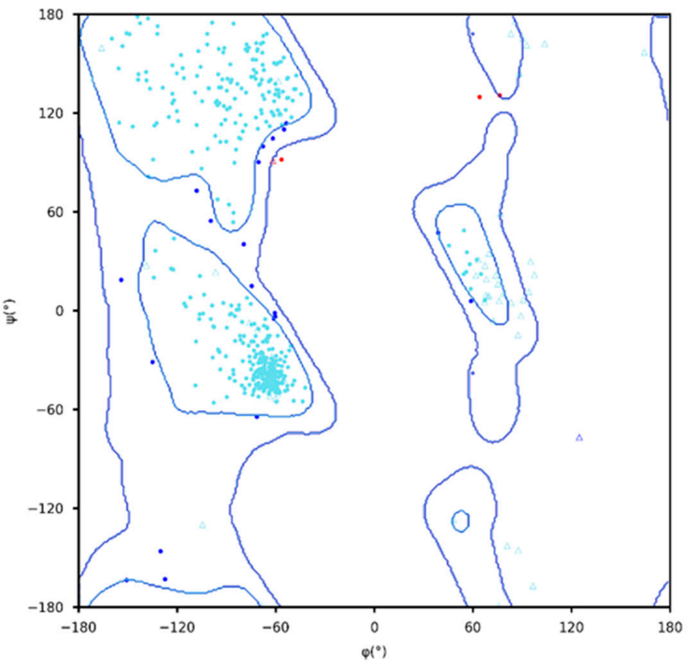

AN11G11290

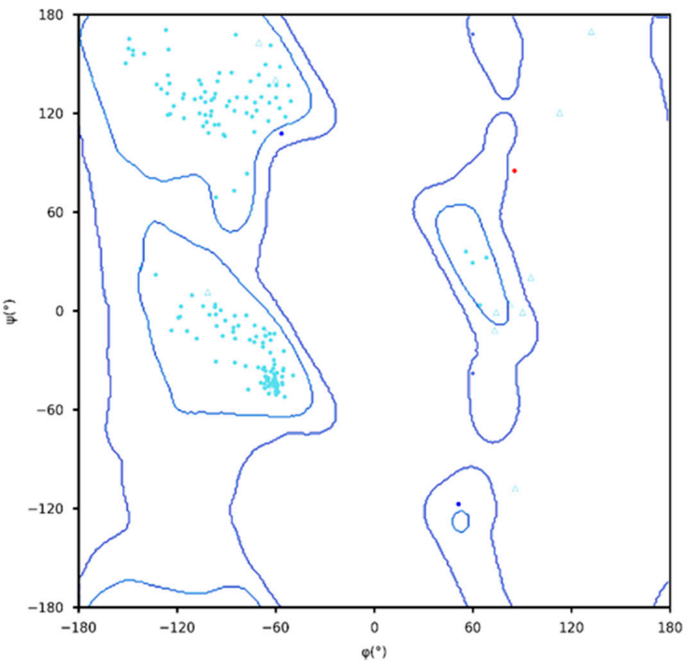

ARF1

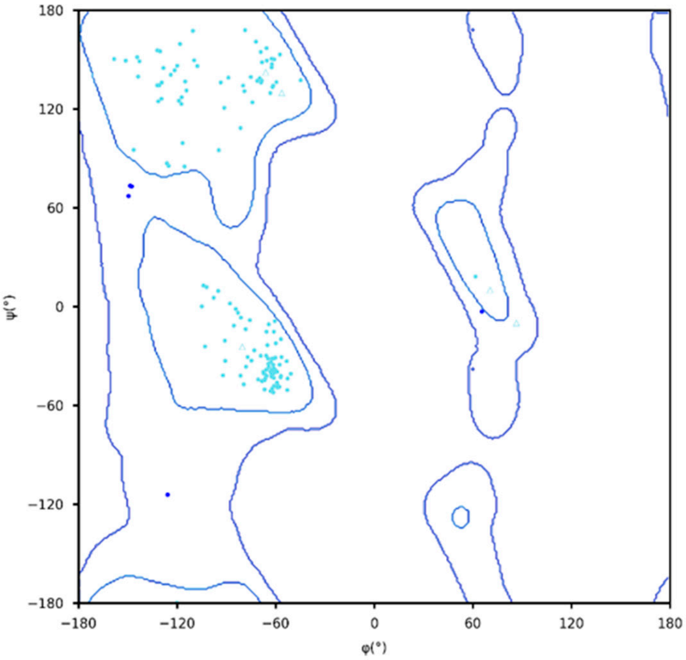
ψ(°)
φ(°)
180
120
60
0
−60
−120
−180
−180
−120
−60
0
60
120
180

CDC34

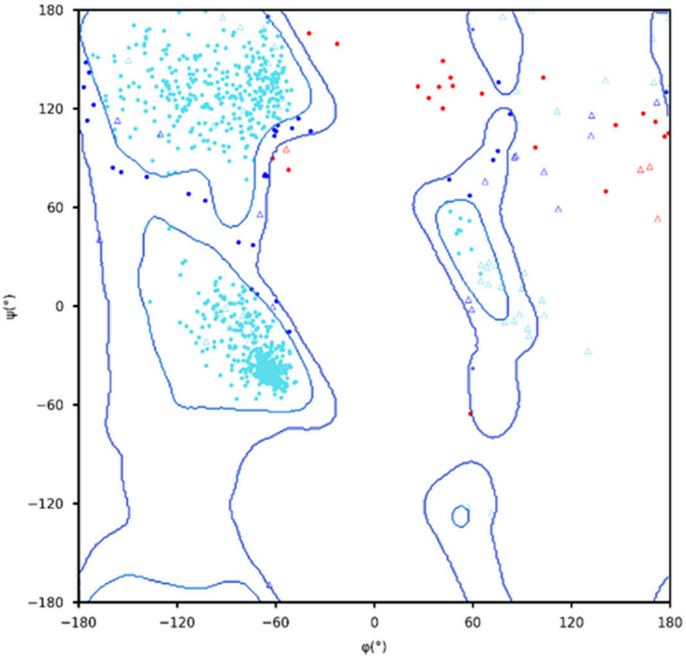
ψ(°)
φ(°)
180
120
60
0
−60
−120
−180
−180
−120
−60
0
60
120
180

DDX5

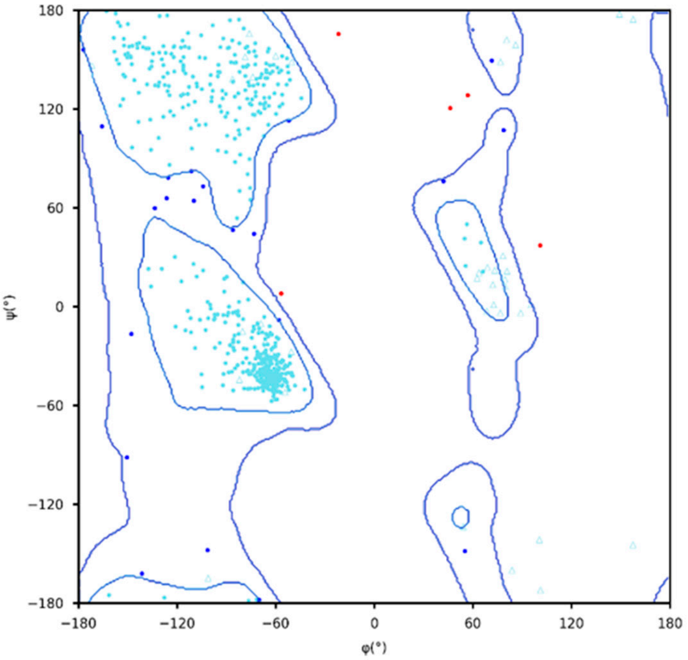

180
120
60
0
−60
−120
−180
ψ(°)
−180
−120
−60
0
60
120
180
φ(°)

DLD1

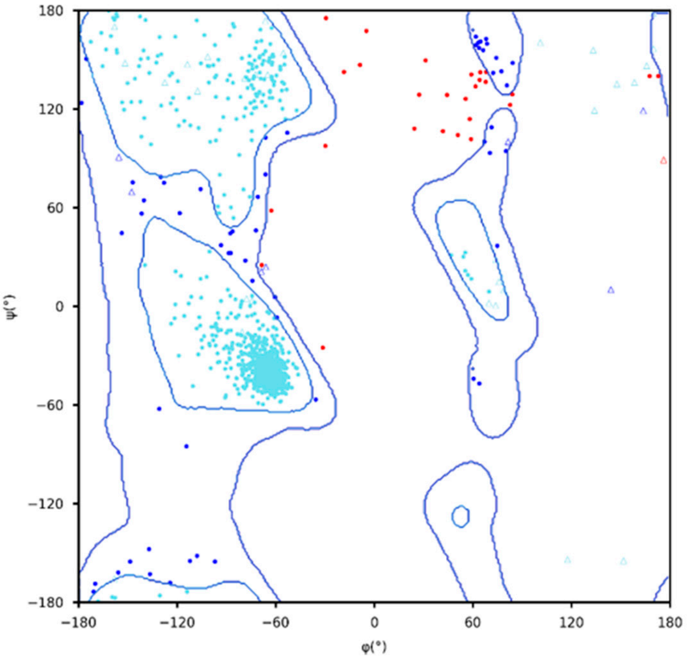

180
120
60
0
−60
−120
−180
ψ(°)
−180
−120
−60
0
60
120
180
φ(°)

EIF3S9

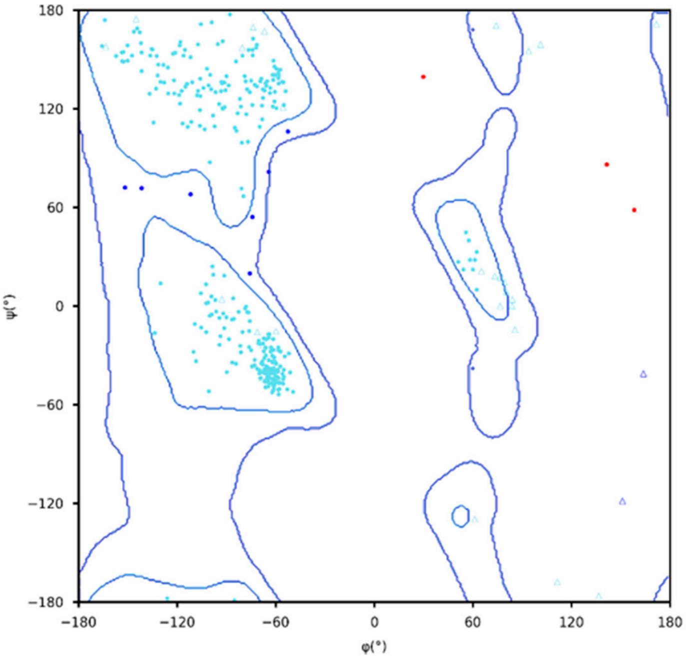

GLN1

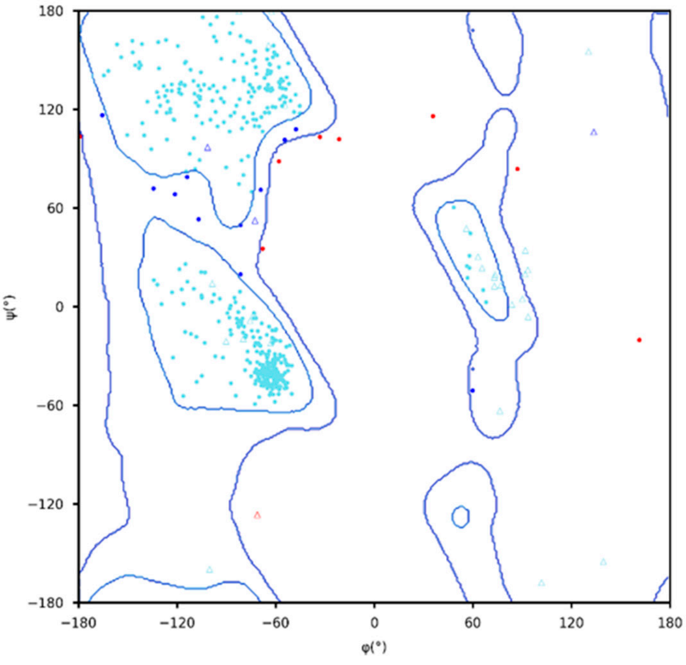

Histone Deacetylase (HDA)

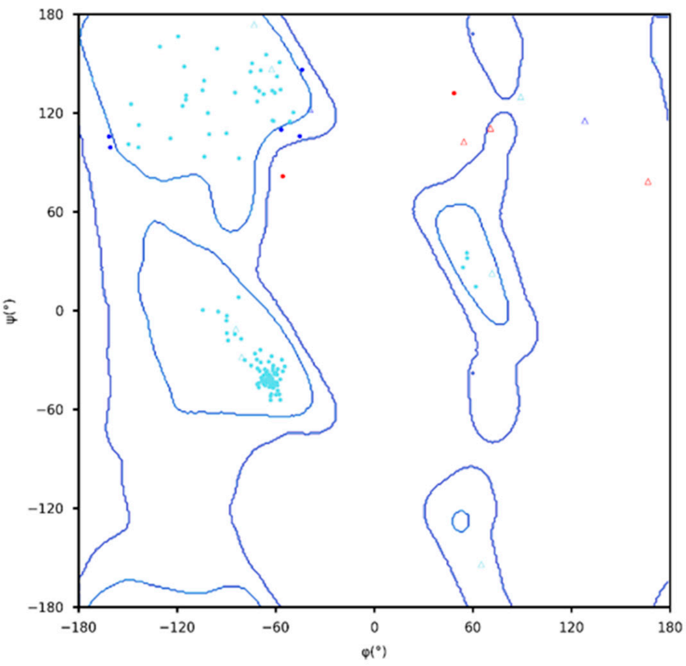
180
120
60
0
−60
−120
−180
−180
−120
−60
0
60
120
180
ψ(°)
φ(°)

HTA1

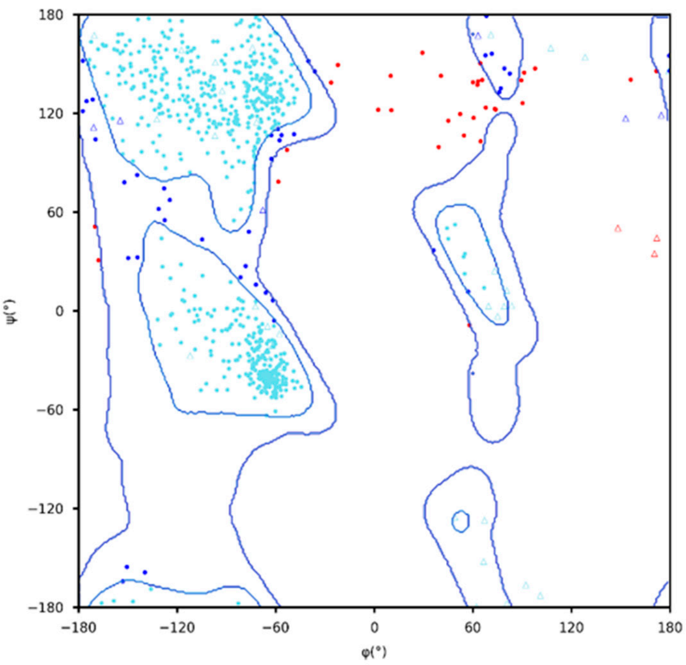
180
120
60
0
−60
−120
−180
−180
−120
−60
0
60
120
180
ψ(°)
φ(°)

PLC1

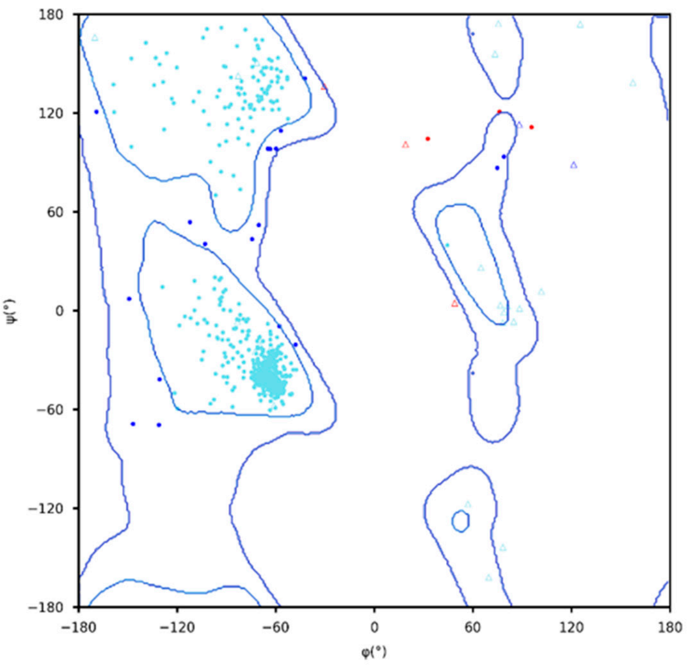
180
120
60
0
−60
−120
−180
ψ(°)
−180
−120
−60
0
60
120
180
φ(°)

PTR2

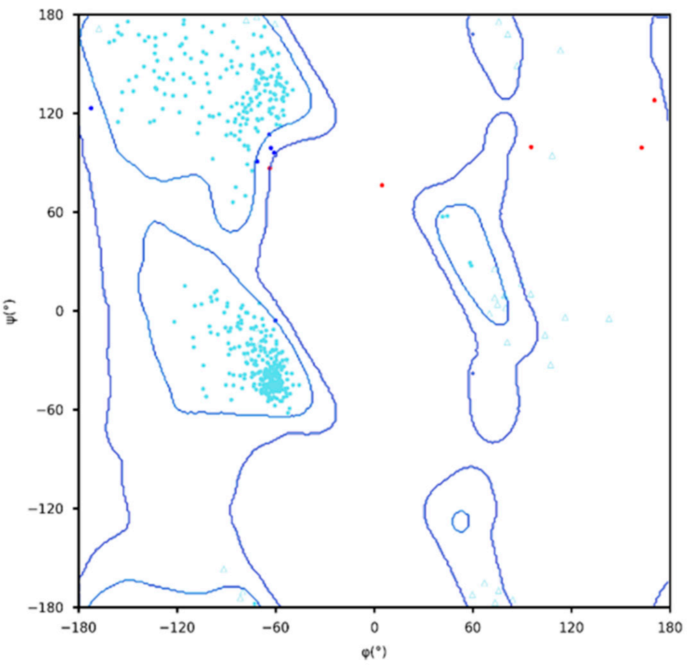
180
120
60
0
−60
−120
−180
ψ(°)
−180
−120
−60
0
60
120
180
φ(°)

RAM1

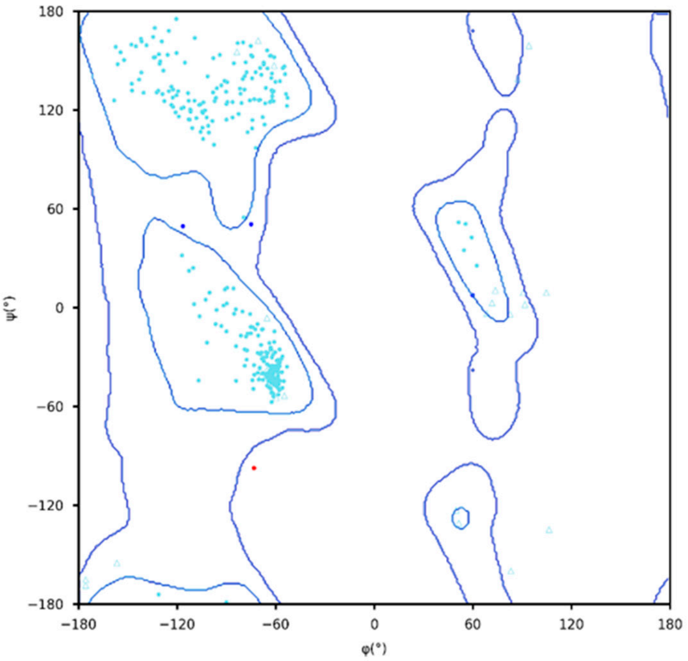
180
120
60
0
−60
−120
−180
ψ(°)
−180
−120
−60
0
60
120
180
φ(°)

RBK1

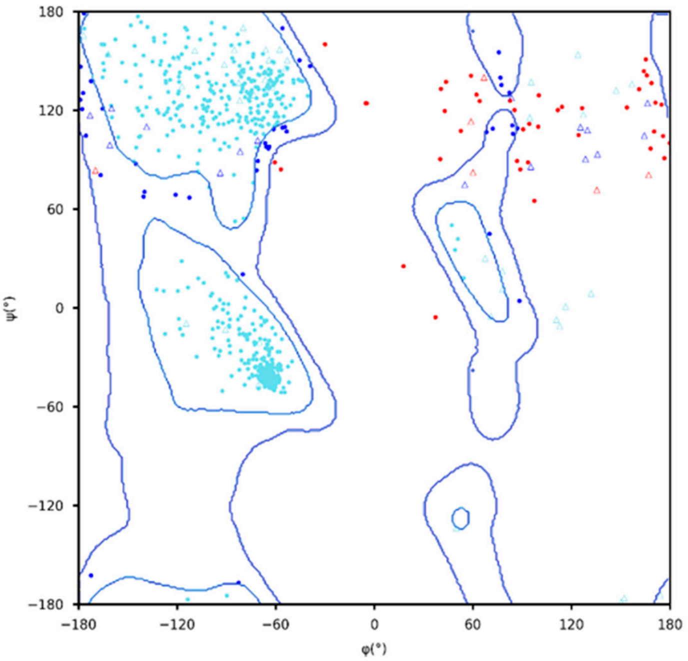
180
120
60
0
−60
−120
−180
ψ(°)
−180
−120
−60
0
60
120
180
φ(°)

RBM27

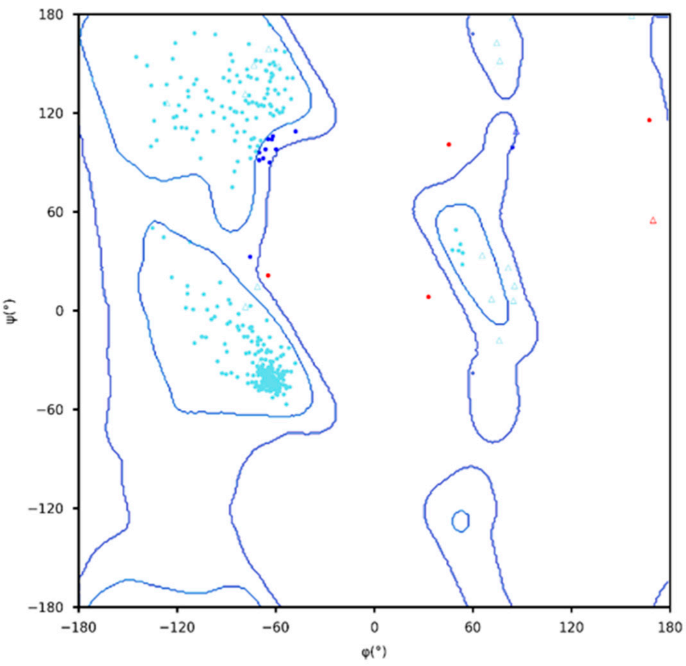

RFC3

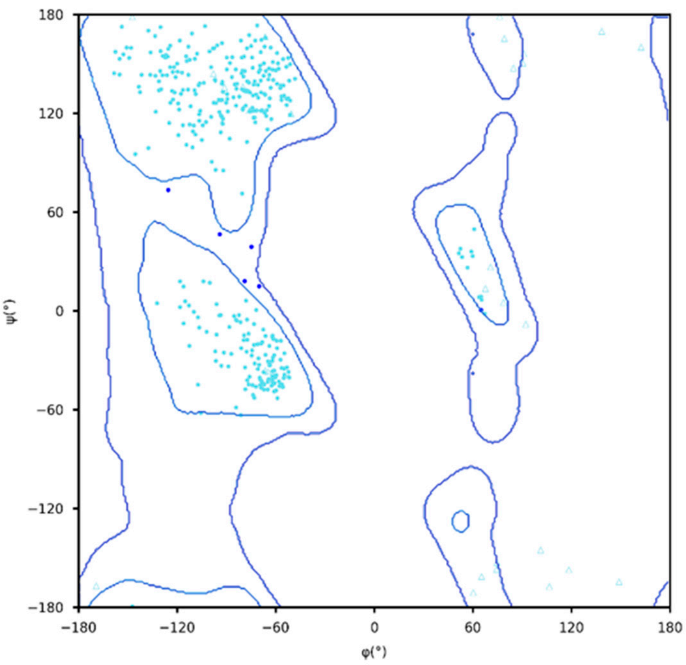

RPL3

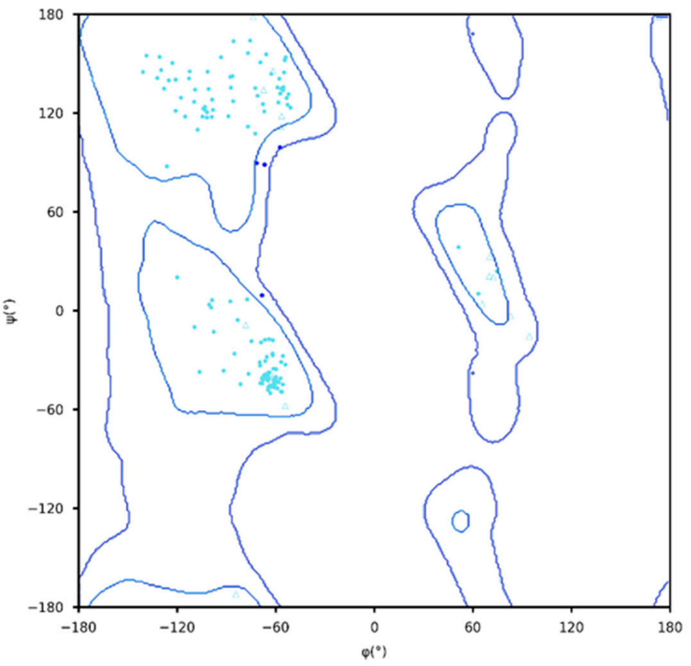
180
120
60
0
−60
−120
−180
ψ(°)
−180
−120
−60
0
60
120
180
φ(°)

RPL11

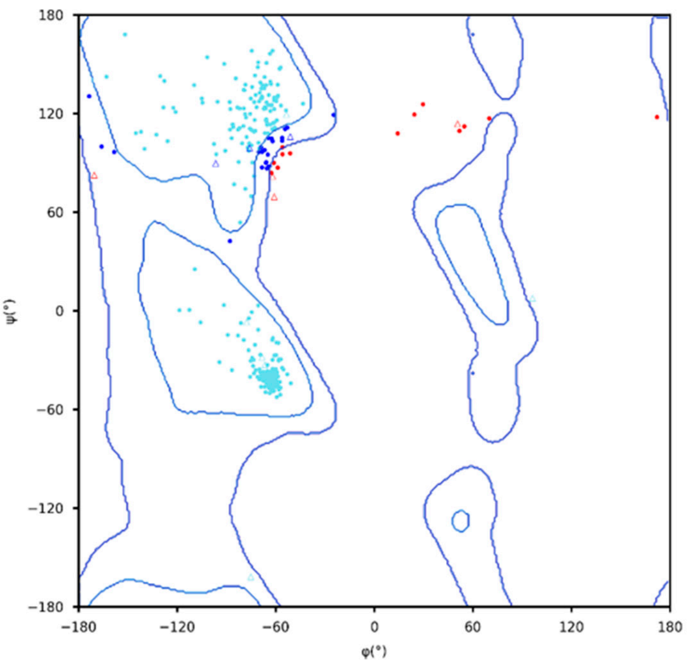
180
120
60
0
−60
−120
−180
ψ(°)
−180
−120
−60
0
60
120
180
φ(°)

RRP5

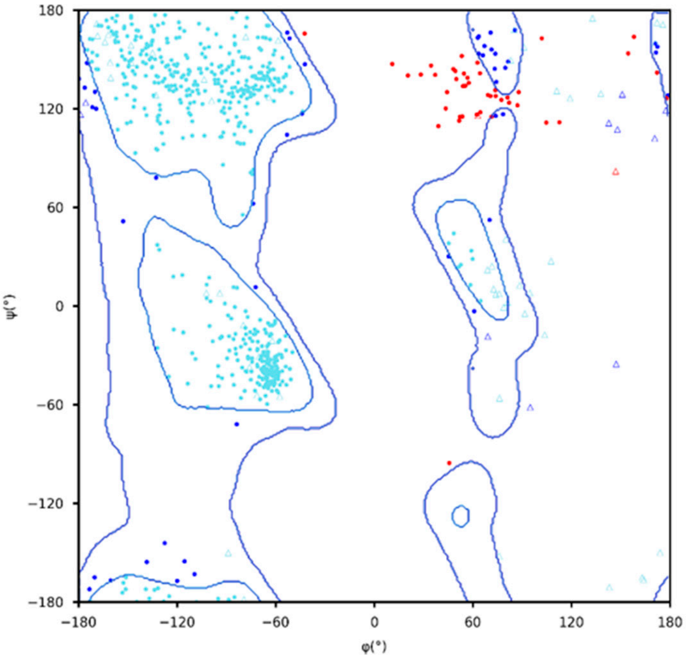

SI:DKEY-3708.1

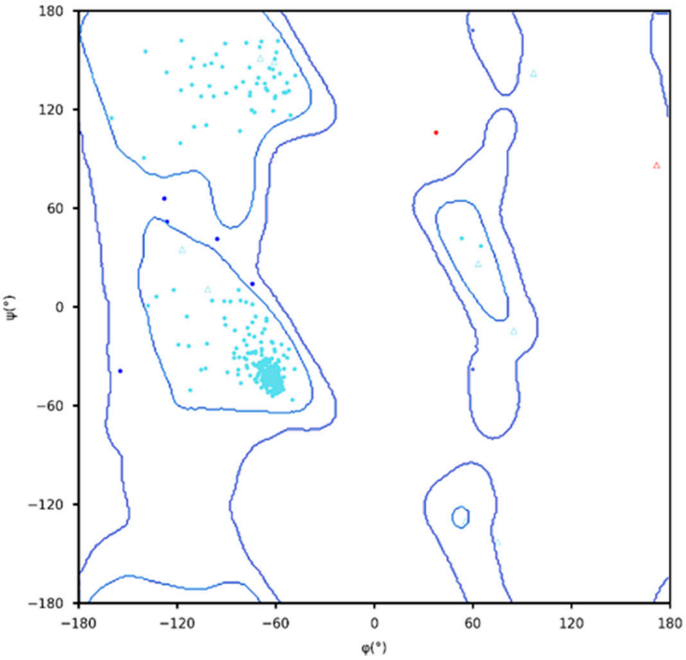

SRD5A3

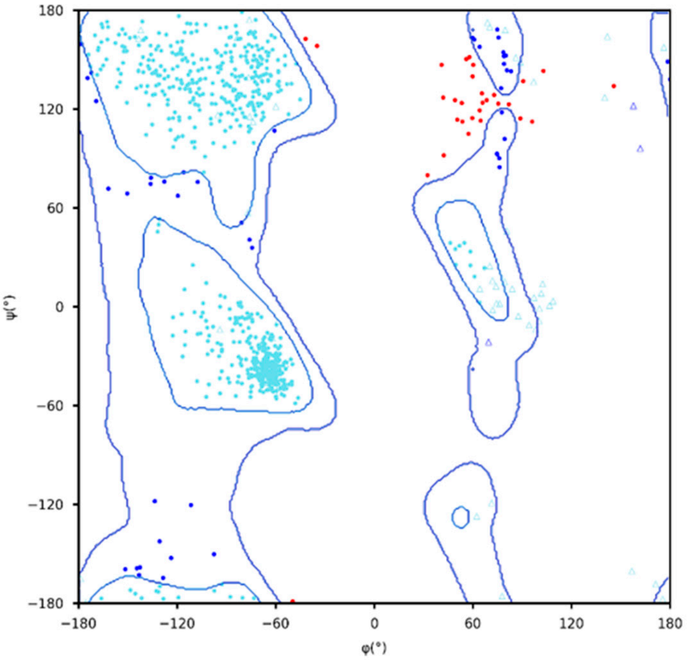
180
120
60
0
−60
−120
−180
ψ(°)
−180
−120
−60
0
60
120
180
φ(°)

TFP1

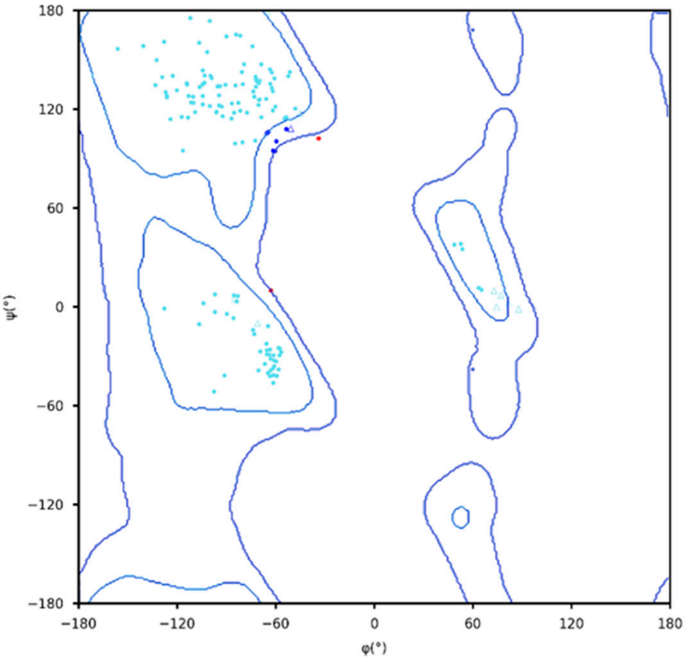
180
120
60
0
−60
−120
−180
ψ(°)
−180
−120
−60
0
60
120
180
φ(°)

UBA52
